# Genomic Architecture Predicts Tree Topology, Population Structuring, and Demographic History in Amazonian Birds

**DOI:** 10.1093/gbe/evae002

**Published:** 2024-01-17

**Authors:** Gregory Thom, Lucas Rocha Moreira, Romina Batista, Marcelo Gehara, Alexandre Aleixo, Brian Tilston Smith

**Affiliations:** Department of Ornithology, American Museum of Natural History, New York, NY, USA; Museum of Natural Science, Louisiana State University, Baton Rouge, LA, USA; Department of Biological Sciences, Louisiana State University, Baton Rouge, LA, USA; Program in Bioinformatics and Integrative Biology, University of Massachusetts Chan Medical School, Worcester, MA, USA; Department of Vertebrate Genomics, Broad Institute of MIT and Harvard, Cambridge, MA, USA; Programa de Coleções Biológicas, Instituto Nacional de Pesquisas da Amazônia, Manaus, Brazil; School of Science, Engineering and Environment, University of Salford, Manchester, UK; Department of Earth and Environmental Sciences, Rutgers University, Newark, NJ, USA; Finnish Museum of Natural History, University of Helsinki, Helsinki, Finland; Department of Environmental Genomics, Instituto Tecnológico Vale, Belém, Brazil; Department of Ornithology, American Museum of Natural History, New York, NY, USA

**Keywords:** phylogenomics, recombination rate, gene flow, natural selection, chromosomes

## Abstract

Geographic barriers are frequently invoked to explain genetic structuring across the landscape. However, inferences on the spatial and temporal origins of population variation have been largely limited to evolutionary neutral models, ignoring the potential role of natural selection and intrinsic genomic processes known as genomic architecture in producing heterogeneity in differentiation across the genome. To test how variation in genomic characteristics (e.g. recombination rate) impacts our ability to reconstruct general patterns of differentiation between species that cooccur across geographic barriers, we sequenced the whole genomes of multiple bird populations that are distributed across rivers in southeastern Amazonia. We found that phylogenetic relationships within species and demographic parameters varied across the genome in predictable ways. Genetic diversity was positively associated with recombination rate and negatively associated with species tree support. Gene flow was less pervasive in genomic regions of low recombination, making these windows more likely to retain patterns of population structuring that matched the species tree. We further found that approximately a third of the genome showed evidence of selective sweeps and linked selection, skewing genome-wide estimates of effective population sizes and gene flow between populations toward lower values. In sum, we showed that the effects of intrinsic genomic characteristics and selection can be disentangled from neutral processes to elucidate spatial patterns of population differentiation.

**Significance**

Modeling the spatial history of populations has traditionally relied on neutrally evolving genomic regions, while the effects of intrinsic and extrinsic genomic processes, such as recombination and selection, are rarely accounted for. These processes are known to impact levels of diversity and the degree of differentiation between populations and play an important role in shaping phylogenetic signals across the genome. Here, we show the impact of recombination and selection on inferring patterns in Amazonian birds.

## Introduction

Genetic patterns inferred from neutrally evolving portions of the genome have played a fundamental role in the development of hypotheses explaining how species form across geographic barriers (Aleixo 2004; Ribas et al. 2012; Silva et al. 2019). For example, in the Amazon, the most biodiverse region on the planet, genetic structuring across large rivers has been central to studying how diversity originates in tropical forests (Haffer 1969, 2008; Ribas et al. 2012; Smith et al. 2014; Silva et al. 2019). A collective body of work shows that species isolated by biogeographic barriers such as Amazonian large rivers have highly variable relationships that span millions of years with limited congruence in evolutionary histories across the landscape (Smith et al. 2014; Silva et al. 2019). Recent studies using genome-wide markers have further highlighted additional complexities in inferring population history by identifying that gene flow across barriers is a common process (Weir et al. 2015; Barrera-Guzmán et al. 2018; Ferreira et al. 2018; Berv et al. 2021; Del-Rio et al. 2021; Luna et al. 2021; Musher et al. 2021). In addition to gene flow, intrinsic (e.g. recombination rate) and extrinsic (e.g. selection) processes that influence the landscape of genomic diversity and differentiation, herein referred to as genomic architecture, may further obfuscate biogeographic inferences by affecting the estimation of phylogenetic and demographic parameters (Li et al. 2019; Martin et al. 2019). Elucidating the relationships between genomic architecture and the neutral and adaptive processes driving genomic evolution may yield more accurate inferences on the geographic and temporal histories of species, providing increased resolution into species origins.

A heterogeneous landscape of genetic diversity is a commonly recovered pattern across taxonomic groups, indicating that evolutionary parameters vary across the genome (Delmore et al. 2018; Li et al. 2019; Martin et al. 2019; Johri et al. 2021; Manthey et al. 2021). Components of genomic architecture, such as chromosomal inheritance, meiotic recombination, the density of targets of selection, biased gene conversion, and mutation rate, operate simultaneously and heterogeneously across the genome, resulting in highly variable levels of genetic diversity and differentiation at both intra- and interspecific scales (Meunier and Duret 2004; Garrigan et al. 2012; Cruickshank and Hahn 2014; Roux et al. 2014; Seehausen et al. 2014; Fontaine et al. 2015; Wolf and Ellegren 2017; Smith et al. 2018; Edelman et al. 2019; Martin et al. 2019; Johri et al. 2021). For instance, recent evidence indicates that phylogenetic signal (e.g. the support for a particular topology) is associated with chromosome size and recombination rate, with larger chromosomes having a slower recombination rate and higher support for the species trees (Martin et al. 2019). However, most tree-building methods do not account for the multiple processes that shape the genomic landscape, which may confound the estimation of evolutionary histories (Ewing and Jensen 2016; Schrider et al. 2016; Li et al. 2019). Understanding how genomic architecture may affect inferences on population differentiation will provide a clearer picture of the relative roles of intrinsic genomic characteristics, natural selection, and neutral processes in speciation.

Linked selection can have a large impact on genome-wide variation, but its effects on phylogenetic signal and inferring the demographic history of species are only starting to be explored (Li et al. 2019; Martin et al. 2019). The indirect influence of selection on linked neutral sites can reduce genetic diversity around target regions, decrease local effective population size (*N*_e_), and lead to faster fixation of alleles (Charlesworth 1998; Cruickshank and Hahn 2014; Burri et al. 2015). The intensity of linked selection on neutral sites is predicted by the interplay between the local density of targets under selection and the recombination rate, with more pronounced reductions in genetic diversity occurring in genomic regions with stronger selection and lower recombination (Smith and Haigh 1974; Charlesworth et al. 1993; Hudson and Kaplan 1995; Gillespie 2000; Zeng 2013). Areas of low recombination should also be more resistant to the confounding effects of gene flow and function as hotspots of phylogenetic signal (Martin et al. 2019; Chase et al. 2021). In these regions, linkage is maintained between introgressed variants, and large genomic blocks may be removed from a population if deleterious alleles are present (Brandvain et al. 2014; Schumer et al. 2017; Mořkovský et al. 2018). The reduced impact of gene flow in regions of low recombination indicates that the phylogenetic signal is more likely to follow a bifurcating tree model, fitting the assumptions of most phylogenetic methods (Li et al. 2019; Martin et al. 2019). However, linked selection in low recombination areas violates neutral models of evolution and affects genome-wide estimations of demographic parameters (Schrider et al. 2016; Johri et al. 2020).

Although recent studies show that linked selection impacts a larger proportion of the genome than previously thought (Kern and Hahn 2018; Pouyet et al. 2018), the degree of this impact varies between species (Jensen et al. 2019; Tigano et al. 2022). For instance, the divergence between populations with high rates of gene flow might be restricted to small areas of the genome, maintained by strong divergent selection, whereas the vast majority of the genome might show reduced differentiation due to widespread introgression (Ellegren et al. 2012). In contrast, genomic differentiation between populations with reduced levels of gene flow tends to be more widespread, given the higher contribution of genetic drift in isolated populations. This latter scenario should produce a stronger association between genomic architecture and levels of genetic differentiation across the genome.

In this study, we model the impact of recombination and selection on patterns of genetic diversity and population differentiation in 3 bird species with distinct ecologies that cooccur across riverine barriers in the southeastern Amazonia. These taxa have different propensities to move across space, which might result in landscapes of genomic differentiation impacted by distinct levels of gene flow. We hypothesize that if linked selection led to congruent patterns of genetic diversity across the genome of distinct species, then metrics associated with population differentiation and genetic diversity should be correlated with recombination rate, the density of targets under selection, and chromosome size. Alternatively, species could have idiosyncratic patterns of association with genomic architecture, driven by other factors such as historical demography and the level of differentiation across rivers. We expect that areas of the genome more likely to be affected by introgression (e.g. smaller chromosomes and regions with a higher recombination rate) will support topologies that differ from the species trees due to the stronger signature of gene flow. We also predict that areas more likely to be impacted by linked selection, such as regions with lower recombination, despite having a better fit to bifurcating phylogenetic methods, will violate neutral models of evolution and lead to biased demographic parameters. Support for these expectations will provide further strategies for analyzing whole-genome data in a geographic context and indicate that distinct regions of the genome should be targeted to answer questions on distinct temporal scales (e.g. phylogenetic vs. population genetics scales). We demonstrate that the interplay between recombination, selection, and gene flow leads to a highly variable landscape of genetic diversity and differentiation within and between species and impacts evolutionary inferences under different population histories.

## Results

### Sampling and Bioinformatics

**Fig. 1.**
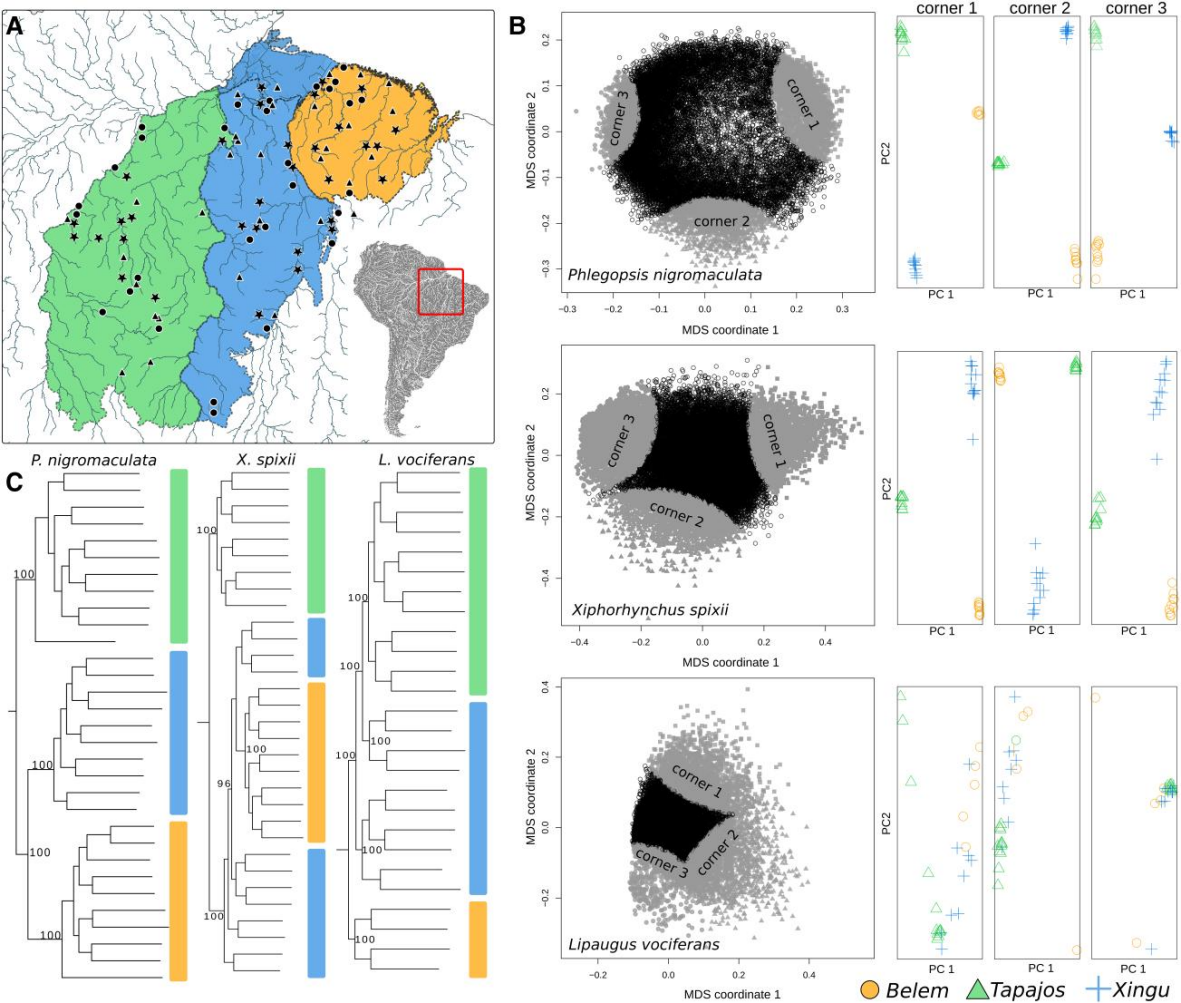
Contrasting patterns of genomic differentiation and relationships between populations of 3 species of birds occurring in southeastern Amazonia. Geographic distribution of genomic samples for each species a). Triangles, stars, and circles are sampled localities for *P. nigromaculata*, *X. spixii*, and *L. vociferans*, respectively. Each colored polygon in the map represents a major Amazonian interfluve (area of endemism): Tapajos (left), Xingu (center), and Belem (right). b) Patterns of genetic structure across the genome were obtained with local PCAs based on 1,000 Single Nucleotide Polymorphism (SNP) windows. Left: plots for the first and second multidimensional coordinates, where each point represents a genomic window. Corners 1–3 represent clusters with 10% of the windows closer to the 3 further points in the graph. Right: PCA plots for the first and second principal components, combining the windows of each corner. c) Supermatrix phylogenetic estimations based on concatenated SNPs. Numbers on the nodes represent bootstrap support for major nodes in the tree. Color bars next to terminals represent geographic location following the map a).

We resequenced 96 whole genomes for 3 species of birds, *Phlegopsis nigromaculata* (*n* = 33; mean coverage = 10.5× with SD = 1.95; mean missing proportion = 0.17 with SD = 0.07), *Xiphorhynchus spixii* (*n* = 33; mean coverage = 11.0× with SD = 1.72; mean missing proportion = 0.16 with SD = 0.07), and *Lipaugus vociferans* (*n* = 30; mean coverage = 9.40× with SD = 1.73; mean missing proportion = 0.19 with SD = 0.09), that are codistributed across 3 Amazonian areas of endemism, the Tapajos, Xingu, and Belem (Fig. 1; supplementary table S1, Supplementary Material online). Our samples consisted of tissues preserved in ethanol and toe pads from recently collected museum specimens (supplementary table S1, Supplementary Material online). For bioinformatic processing, we used reference genomes from closely related species. For *P. nigromaculata*, we used as a reference the genome of *Rhegmatorhina melanosticta* (Coelho et al. 2019). For *X. spixii*, we used the genome of *Xiphorhynchus elegans*, and for *L. vociferans*, we used the genome of *Cephalopterus ornatus*. Scaffolds from our reference genomes were assigned to pseudochromosomes by ordering and orienting their scaffolds to the chromosomes of the zebra finch (*Taeniopygia guttata*; version taeGut3.2.4) and other bird species (i.e. *Falco peregrinus*, *Strigops habroptilus*, *Passer domesticus*, *T. guttata*, and *Parus major*; Coelho et al. 2019). After an initial bioinformatic assessment, we excluded samples with low coverage (mean coverage <5×) and misidentified individuals, producing a final data set with 31 samples of *P. nigromaculata*, 30 samples of *X. spixii*, and 26 samples of *L. vociferans* (supplementary table S1, Supplementary Material online). On average, 88% of the pseudochromosome reference genomes were recovered with coverage above 5× per individual (supplementary table S1, Supplementary Material online). Benchmarking Universal Single-Copy Orthologs analyses performed in BUSCO v2.0.1 (Waterhouse et al. 2018) identified a high proportion of targeted genes in the references used for *P. nigromaculata* (89.3%), *X. spixii* (89.1%), and *L. vociferans* (93.4%; supplementary table S2, Supplementary Material online). Additional summary statistics and comparisons between tissue types are available in supplementary results, Supplementary Material online.

### Population Genetics Summary Statistics and Genomic Features Vary between Species and across the Genome

**Fig. 2.**
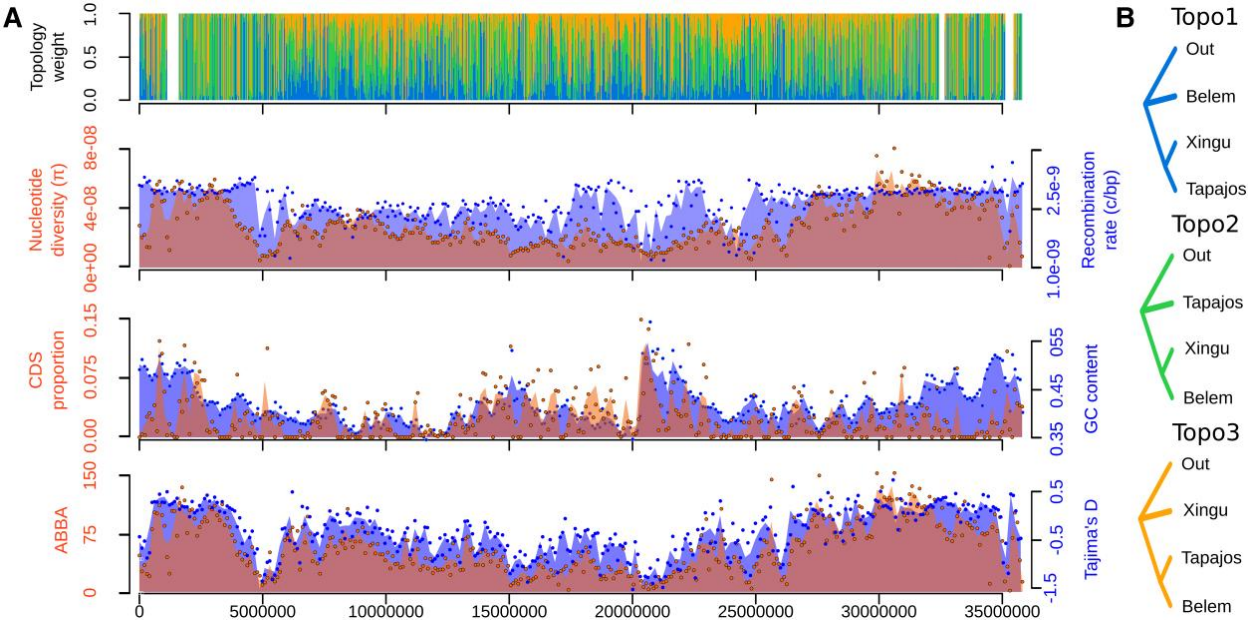
The phylogenetic signal for the species tree was higher on the central portions of chromosomes and was associated with genomic architecture. a) An example of how phylogenetic signals and summary statistics are distributed across a chromosome. Shown is pseudochromosome 6 of *P. nigromaculata*. On the top graph, colored bars represent the weight for the 3 alternative topologies shown in b) for the relationship between Tapajos, Xingu, and Belem areas of endemism. The 3 bottom graphs show summary statistics for the same genomic region. Estimates of nucleotide diversity, recombination rate, and Tajima's *D* were based on the Tapajos population. CDS represents the proportion of coding sequences within a given window; ABBA represents the number of sites supporting topology 2 assuming topology 1 as the species tree.

**Fig. 3.**
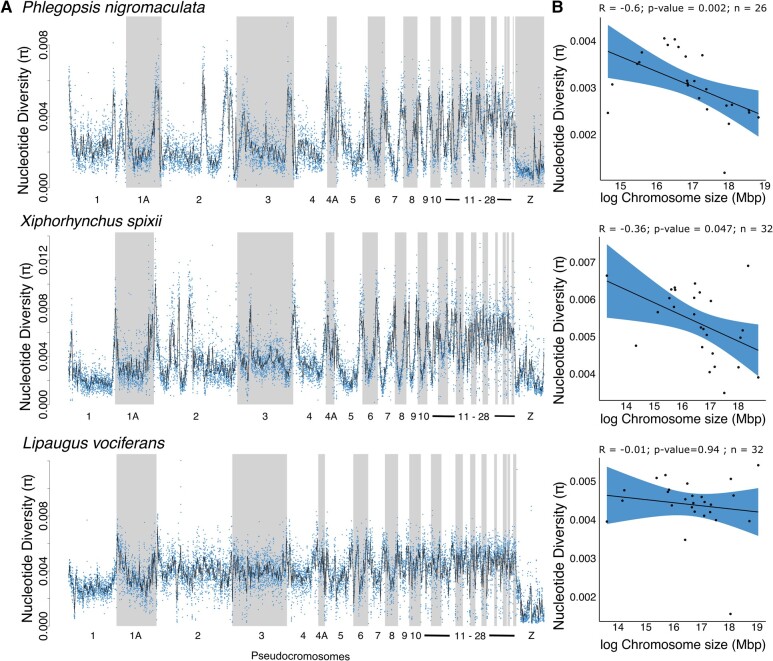
Nucleotide diversity varied within and between pseudochromosomes and across species. a) Distribution of nucleotide diversity (*π*) across chromosomes for the 3 studied species. b) Scatterplot and regression line with 95% confidence interval models with average nucleotide diversity as a function of chromosome size.

Levels of genetic diversity varied substantially between species and within and between chromosomes. To explore how genetic diversity varied across the genome, we calculated summary statistics for 100 kb nonoverlapping sliding windows and mean values per chromosome (Figs. 2 and 3). Populations from species with higher putative dispersal abilities, *L. vociferans* (*π* = 0.0018; SD = 0.0003) and *X. spixii* (*π* = 0.0013; SD = 0.0004), had greater nucleotide diversity than *P. nigromaculata* (*π* = 0.0010; SD = 0.0003; supplementary tables S3 to S5, Supplementary Material online). We observed higher nucleotide diversity on smaller chromosomes in *P. nigromaculata* (Pearson's correlation *R* = −0.6; *P* = 0.002; *n* = 26) and *X. spixii* (Pearson's correlation *R* = −0.36; *P* = 0.047; *n* = 32) but not in *L. vociferans* (Pearson's correlation *R* = −0.01; *P* = 0.94; *n* = 32; supplementary figs. S1 to S6 and tables S6 to S11, Supplementary Material online). We also found similar associations with *D*_XY_, the number of segregation sites, Patterson's *D* statistic (ABBA/BABA), *f*_dm_, and Tajima's *D* (supplementary figs. S1 to S6 and tables S6 to S11, Supplementary Material online). These results support a highly heterogeneous landscape of genetic diversity across genomes.

We found that genomic regions with a reduced meiotic recombination rate had reduced genetic diversity, were less impacted by gene flow, and had greater genetic differentiation. To test for associations between recombination rate and genetic metrics while accounting for historical demography, we estimated the per-base recombination rate (*r*) with ReLERNN (Adrion et al. 2020). This method uses recurrent neural networks to estimate recombination rates across windows of the genome, accounting for the historical demography of populations. General linear models found a strong association between predicted and simulated recombination rates (mean *R*^2^ = 0.90; *n* = 1,000) with low mean absolute error (mean MAE = 0.21) supporting relatively accurate parameter estimation. Recombination rate varied considerably across the genomes of *P. nigromaculata* (mean *r* = 2.1 × 10^−9^; SD = 4.4 × 10^−10^), *X. spixii* (mean *r* = 1.2 × 10^−9^; SD = 7.2 × 10^−10^), and *L. vociferans* (mean *r* = 1.8 × 10^−9^; SD = 4.0 × 10^−10^), but in predictable ways (Fig. 2; supplementary fig. S7, Supplementary Material online). We found that regions with higher recombination rates were often on smaller chromosomes and were positively correlated with gene density and nucleotide diversity in all 3 species (supplementary figs. S4 to S8 and table S12, Supplementary Material online). LOESS models with recombination rate and gene density as covariate predictors explained a large proportion of the variation in genetic diversity in *P. nigromaculata* (*R*^2^ = 0.33), *X. spixii* (*R*^2^ = 0.65), and *L. vociferans* (*R*^2^ = 0.41; supplementary figs. S8 and S9, Supplementary Material online).

Although the association between genetic diversity and recombination rate might indicate a significant contribution of linked selection across the genome, population-based methods, such as ReLERNN, might inflate this relationship. ReLERNN (Adrion et al. 2020) estimates recombination rates expressed in terms that can be confounded with effective population size (i.e. *rho* = 4*N*_e_*r*, where *rho* is the population recombination rate, *N*_e_ is the effective population size, and *r* is the recombination rate). Although ReLERNN estimates *r* by calculating *N*_e_ (i.e. *theta*) from the observed data, the circularity of this approach could impact our conclusions. To further test the impact of linked selection in driving the association between recombination rate and genetic diversity, we built a LOESS model using only areas assigned as neutral with high probability by diploS/HIC (see below). Given that, in the absence of selection, we did not predict an association between recombination rate and genetic diversity, we expected neutral areas to have a reduced association between these parameters. In all 3 species, we found that modeling genetic diversity and recombination rate explained a higher proportion of the variance when considering randomly sampled windows across the genome than when sampling only neutral regions (supplementary table S13, Supplementary Material online). This result was consistent with the effect of linked selection across the genome.

### Population Differentiation Is Associated with Recombination Rate

Genetic structure and levels of population differentiation varied substantially across the genome and were associated with intrinsic genomic processes. To explore the genome-wide variation in genetic structure, we used local principal component analyses (PCAs) across sliding windows with 1,000 Single Nucleotide Polymorphisms (SNPs) using lostruct v0.0.0.9 (Li and Ralph 2019). The average physical length of windows varied among species: *P. nigromaculata* (44,218 bp), *X. spixii* (35,592 bp), and *L. vociferans* (42,212 bp). We used windows with a fixed number of SNPs instead of a fixed length to ensure all windows would have comparable genetic information. Local PCAs showed that distinct parts of the genome support different clustering patterns in *P. nigromaculata* and *X. spixii* (Fig. 1). In *L. vociferans*, we observed a gradient between Tapajos, Xingu, and Belem individuals without clear geographic structuring, consistent with the low *F*_ST_ estimates reported for this species (Fig. 1; see supplementary results, Supplementary Material online). Patterns of genetic structure, as described by the first 2 multidimensional scaling (MDS) axes calculated from lostruct, were associated with recombination rate in *P. nigromaculata* (MDS1: *R*^2^ = −0.04, *P* < 0.0001, *n* = 20,143 windows) and *X. spixii* (MDS2: *R*^2^ = −0.15, *P* < 0.0001, *n* = 28,803 windows) but not in *L. vociferans* (*R*^2^ < 0.001 for all MDSs, *n* = 25,007 windows). A similar pattern was observed when comparing the average recombination rate between distinct MDS corners (supplementary fig. S10, Supplementary Material online). In *P. nigromaculata*, corner 1 had a significantly lower recombination rate, while corner 2 had a significantly higher rate than the rest of the genome (supplementary fig. S10, Supplementary Material online). In *X. spixii*, corner 2 had a higher rate of recombination than the remaining windows of the genome. Similarly, pairwise *F*_ST_ between populations was, in general, negatively correlated with genetic diversity metrics in all 3 species, and it was negatively correlated with the recombination rate in *P. nigromaculata* and *X. spixii* (supplementary figs. S1 to S6 and tables S6 to S12, Supplementary Material online). This result indicated that for species with marked genetic structure across rivers, recombination rate was a key predictor of spatial differentiation and clustering.

### Widespread Signatures of Selection across the Genome

**Fig. 4.**
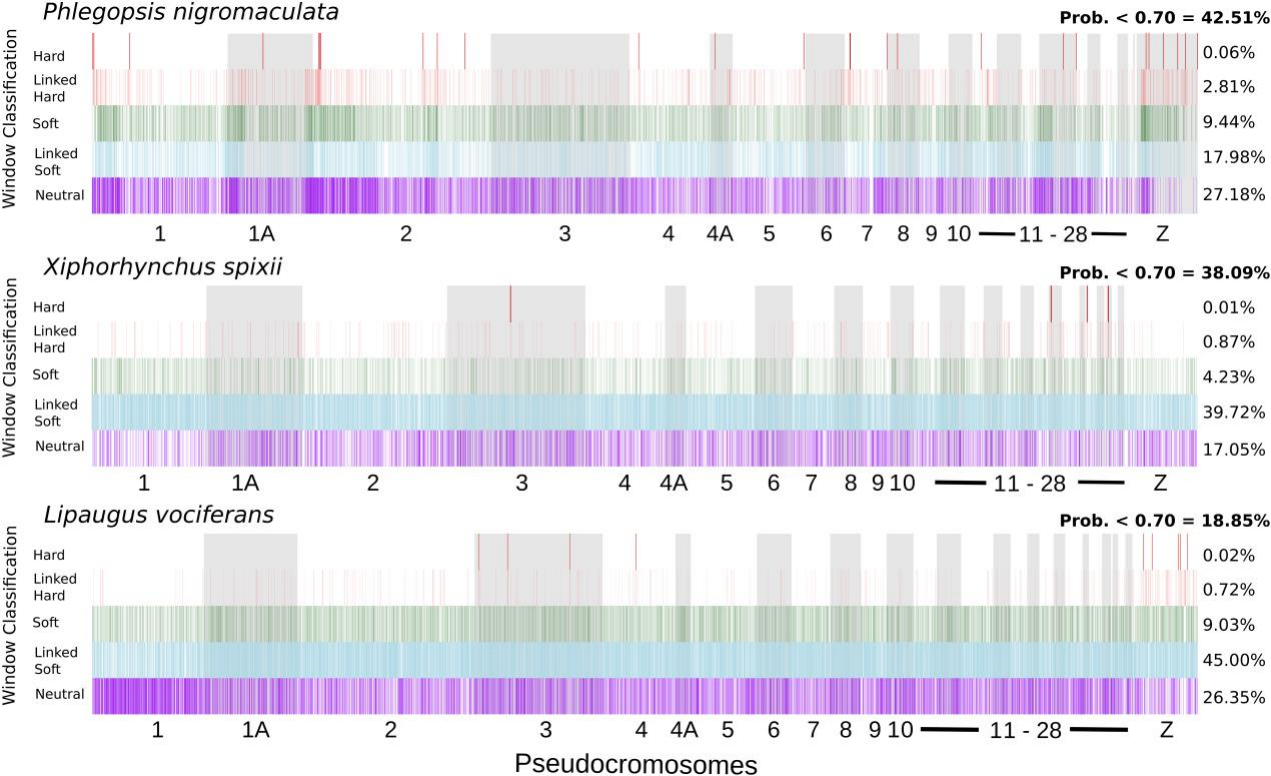
Signature of selection across the genomes of the studied species. Vertical bars represent the model with the highest probability for 20 kb genomic windows. On the right in bold is the proportion of windows with low probability for model classification, followed by the percentage of windows assigned to each of the 5 models with high probability (>0.70): hard sweep, linked to hard sweep, soft sweep, linked to soft sweep, and neutral.

Although an association between recombination rate and genetic diversity supports linked selection as an important mechanism in shaping levels of genetic diversity across the genome, it does not indicate which portions of the genome are directly impacted by this process. To further explore the extent of linked selection across the genome, we used a machine learning approach implemented on diploS/HIC (Kern and Schrider 2018) to predict which 20 kb genomic windows were evolving under neutrality or had signatures of selective sweeps and linked selection. diploS/HIC implements a deep convolutional neural network (CNN) to identify sweeps and genetic variation linked to sweeps by summarizing observed and simulated data into population genetic summary statistics, taking into account the demographic history and recombination rate (Kern and Schrider 2018). To obtain an overall landscape of selection per species, we ran diploS/HIC just for the Tapajos populations, which were represented exclusively by high-quality tissue samples. The demographic history of Tapajos populations was estimated with SMC++ v1.15.3 (Terhorst et al. 2017). The CNN implemented in diploS/HIC produced an average accuracy for model classification of 69% (68% to 72%) and a false positive rate (FPR) of 27% (25% to 28%) among species (supplementary table S14 and fig. S11, Supplementary Material online). Most of the confusion in model classification occurred between hard sweeps and linked to hard sweeps, which considerably affected the overall accuracy of our approach (supplementary fig. S12, Supplementary Material online). Neutral models were correctly classified 83.6% (82% to 87%) of the time across all species, indicating good accuracy in distinguishing neutral and selected areas of the genome. During the testing step of the neural network, we observed an increase in accuracy (84%) and a reduction in the FPR (15%) when considering only simulations that were assigned to models with a probability >70%. This cutoff allowed us to have increased confidence in model selection without excluding a large proportion of the genome and was used to calculate the overall proportion of the genome impacted by selection. On average, across all 3 species, 43.3% of genomic windows classified with high probability (>70%) were assigned to nonneutral models. In *P. nigromaculata*, 30.29% of tested windows had a high probability for models including the direct or indirect effect of selection (Fig. 4). For *X. spixii* (44.83%) and *L. vociferans* (54.77%), we obtained even higher proportions (Fig. 4). When accounting for a FPR by assuming that all potential false positives were neutral regions classified as having signatures of selection, on average, 36.8% of the sites in the genomes of the analyzed species were classified as nonneutral. We found that regions classified as hard sweeps or linked to hard sweeps were also associated with higher values of the *H* statistic, which tests for long runs of homozygosity, than neutral areas of the genome (supplementary fig. S13, Supplementary Material online). A similar pattern was observed for soft sweeps but not for areas linked to soft sweeps, suggesting that other processes across the genomes (e.g. recombination rate) might reduce the power of summary statistics to track the effect of linked selection across the genome.

Background selection may also play a significant role in shaping patterns of genetic diversity across the genome and likely bias demographic parameter estimations (Pouyet et al. 2018). Although the approach implemented by diploS/HIC does not directly account for background selection, the large proportion of the genome identified as nonneutral in our analyses suggested that background selection was probably being attributed to nonneutral models (i.e. sweeps or linked selection). To explore this scenario, we tested if areas with a high density of exons are more likely to be classified as sweeps or linked to sweeps. The reasoning behind this approach was that areas more likely to be affected by background selection (e.g. regions nearby coding sequences) would fit better with nonneutral models. We built a random forest classification model using randomForest in R to test if the proportion of coding sequences could predict if a window was classified as neutral or under selection/linked selection by diploS/HIC. Our random forest approach had a moderate error rate (33.84%), indicating that the model classification of diploS/HIC could be reasonably predicted by the proportion of coding sequences within a window, which was also validated by a Wilcoxon test (*P* = 0.0012, *n* = 4,199). In addition, the 100 kb windows with all overlapping diploS/HIC subwindows assigned to sweeps and linked selection contained 36.9% of all coding sequences, while the fully neutral 100 kb windows contained only 10.6% of all coding sequences. The 100 kb windows with diploS/HIC subwindows assigned to mixed models (neutral, sweep, and/or linked selection) contained 52.5% of all coding sequences. These results suggested that most coding sequences are in regions with signatures of sweeps and linked selection, which validates that windows more likely to be affected by background selection were assigned to nonneutral models.

### Phylogenetic Signal Was Associated with Genomic Architecture

**Fig. 5.**
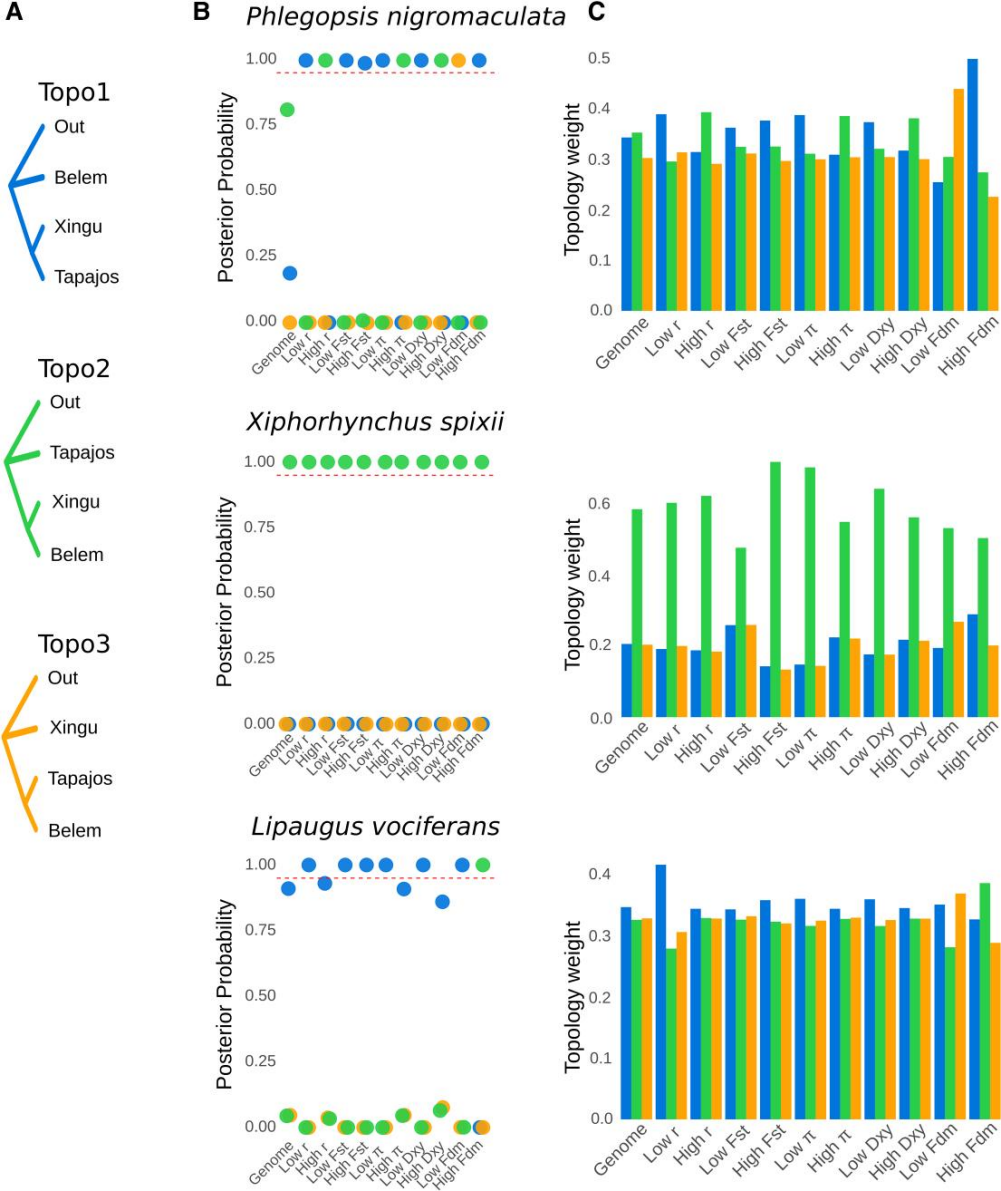
Species tree and topology weights vary accordingly to recombination rate and genetic diversity. a) Alternative topologies for the relationship between the 3 areas of endemism and the outgroup. b) Posterior probabilities for the 3 topologies for windows across the whole-genome and for distinct subsets of genomic windows that were selected based on upper and lower thresholds for summary statistics. Colors represent the 3 alternative topologies in a), and c) weights for 3 topologies for windows across the whole genome and for distinct subsets of genomic windows that were selected based on upper and lower thresholds for summary statistics: recombination rate (*r*); fixation index (*F*_ST_); nucleotide diversity (π); genetic distance (*D*_XY_); and introgression proportion (*f*_dm_).

**Fig. 6.**
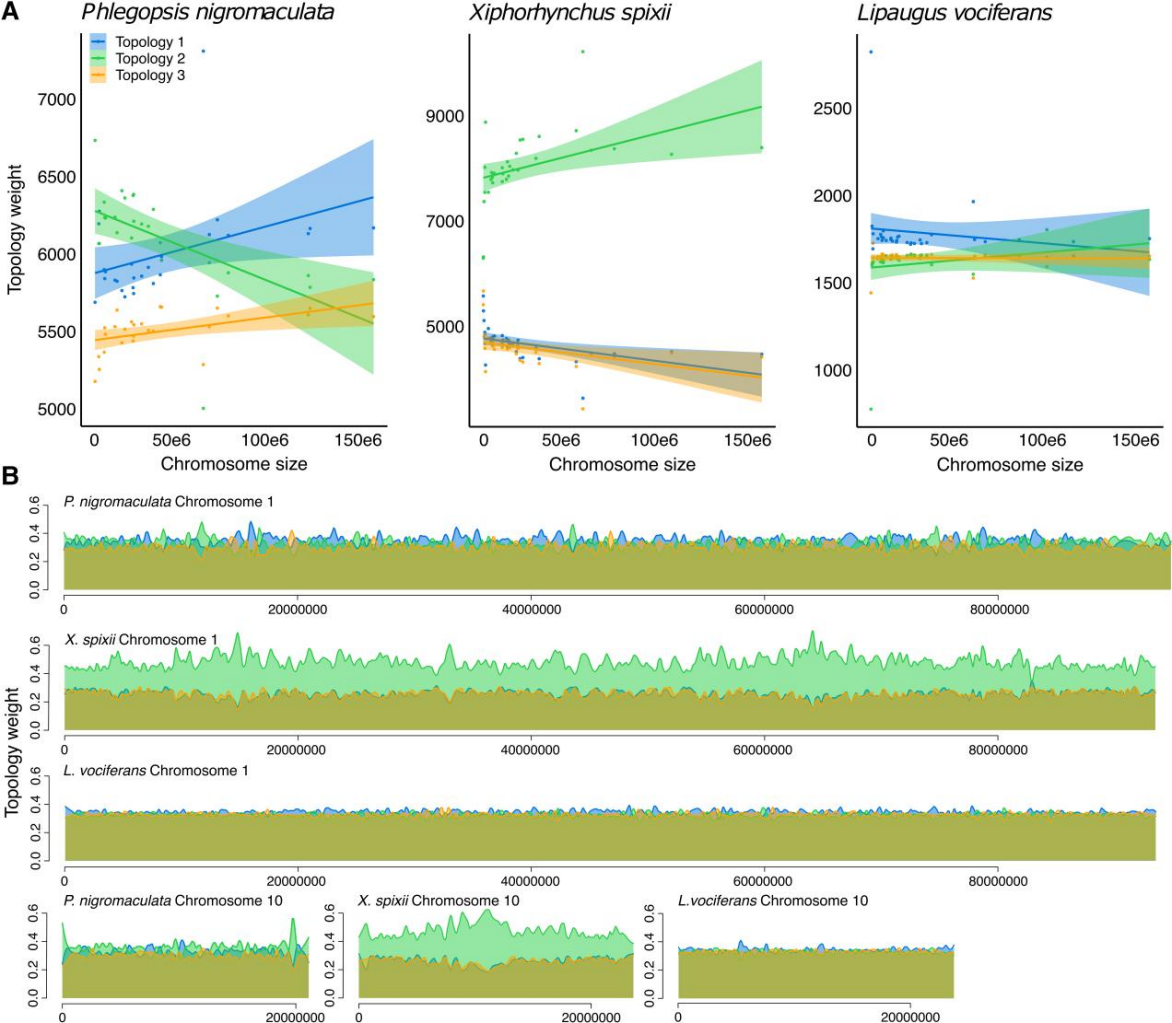
Chromosome size was associated with topology weight across the genome of *P. nigromaculata* (topology 2—*R*^2^ = 0.31, *P* = 0.003; topologies 1 and 2—*P* > 0.05, *n* = 26) but not in *X. spixii* (*P* > 0.05 for all 3 topologies, *n* = 32) or *L. vociferans* (*P* > 0.05 for all 3 topologies, *n* = 32). a) Scatterplot and regression line with 95% confidence interval showing the relationship between topology weights and chromosome size. We tested 3 alternative unrooted topologies for the relationship between the 3 areas of endemism (Tapajos, Xingu, and Belem): topology 1 (outgroup, Belem [Xingu, Tapajos]), topology 2 (outgroup, Tapajos [Xingu, Belem]), and topology 3 (outgroup, Xingu [Belem, Tapajos]). b) Topology weight across a large (Pseudochromosome 1) and small (Pseudochromosome 10) pseudochromosome for the three studied species.

We explored how evolutionary relationships were distributed across the genomes of the cooccurring species to test which aspects of the genomic architecture best predicted the phylogenetic signal. We found that support for alternative topologies varied with recombination rate and genetic diversity (Fig. 5). Our analysis explored the 3 possible unrooted trees representing the relationship between the 3 areas of endemism plus an outgroup. From hereon, we refer to these 3 unrooted topologies as topology 1 (outgroup, Belem [Xingu, Tapajos]), topology 2 (outgroup, Tapajos [Xingu, Belem]), and topology 3 (outgroup, Xingu [Belem, Tapajos]). To explore how phylogenetic relationships varied with genomic characteristics and population genetics summary statistics, we calculated gene trees for nonoverlapping genomic windows and ran species tree analyses for subsets of the genome. For *P. nigromaculata*, we did not obtain high support for any topology in the genome-wide species tree analysis, but the topology with the highest posterior probability (posterior probability = 0.81) matched the concatenated SNP tree (Fig. 5). The topology estimated from genomic regions with high recombination matched the concatenated tree, but regions of low recombination placed Tapajos and Xingu as sisters (Fig. 5). A similar pattern was observed when filtering gene trees based on *π* and *D*_XY_. The phylogenetic signal in *X. spixii* and *L. vociferans* was more stable than *P. nigromaculata*, with widespread support for the same topology across the genome but a substantially higher weight for that topology in areas with lower recombination and lower genetic diversity. The phylogenetic signal also varied with chromosome size in *P. nigromaculata* but not in *X. spixii* or *L. vociferans*. In *P. nigromaculata*, macrochromosomes supported the topology found in low recombination areas (topology 1), while microchromosomes (<50 Mb) supported the concatenated tree (topology 2; supplementary figs. S14 to S16, Supplementary Material online). Similar results were obtained when estimating topology weights across windows of the genome (Fig. 6; see supplementary results, Supplementary Material online).

### Gene Flow Affects Phylogenetic Inference

When modeling gene flow with a coalescent approach in PipeMaster (Gehara et al. 2017), our results indicated that the topology recovered for low recombination areas was the most likely species tree. To estimate the probability for alternative topologies assuming gene flow among nonsister populations, and estimate demographic parameters, we used a multiclass neural network approach with Keras v2.3 (https://github.com/rstudio/keras) in R. We simulated genetic data under the 3 possible unrooted topologies for the relationship between areas of endemism using uniform priors for *N*_e_, gene flow between geographically adjacent populations, and divergence times in PipeMaster (Gehara et al. 2017). To obtain a reduced representation of the genome, we selected 1 10 kb window every 100 kb to limit the effect of linkage between windows. Individuals with missing variants were excluded from the windows, and windows with <3 individuals per population were excluded. We randomly selected 5,000 windows per species from the total number of windows: *P. nigromaculata* (7,213 total), *X. spixii* (9,140), and *L. vociferans* (9,693). Genomic windows were converted into feature vectors representing the mean and variance of commonly used population genetic summary statistics (see Materials and Methods).

On average, the genome-wide model selection approach produced accurate classification probabilities (neural network accuracy = 0.93; categorical cross-entropy = 0.17) and a high correlation between observed and estimated parameters for testing data sets with low MAEs (supplementary tables S15 to S17, Supplementary Material online). PCAs and goodness-of-fit analyses showed that simulated models matched the observed values of summary statistics. In *P. nigromaculata*, we obtained a high probability for topology 1 (probability = 0.86), conflicting with the concatenated and species tree topology (topology 2; probability = 0.12) but agreeing with the topology of low recombination areas (supplementary table S18, Supplementary Material online). Our data indicated that gene flow among *P. nigromaculata* populations (2*Nm*) was negligible between Tapajos and Xingu (migration between Tapajos and Xingu = 0.002; SD = 0.005; MAE = 0.161) and low to moderate between the nonsisters, Xingu and Belem (migration between Xingu and Belem = 0.484; SD = 0.486; MAE = 0.138; supplementary table S15, Supplementary Material online). The relatively reduced levels of gene flow between populations of *P. nigromaculata* indicated that ancestral gene flow might be the source of the phylogenetic conflict. In *X. spixii*, we inferred moderate rates of gene flow between populations, which were highest between the recently diverged Xingu and Belem populations (migration between Xingu and Belem = 2.075; SD = 0.144; MAE = 0.139; supplementary table S16, Supplementary Material online). In *L. vociferans*, we also found moderate to high gene flow among populations (migration between Tapajos and Xingu = 2.347; MAE = 0.175; migration between Xingu and Belem = 1.827; MAE = 0.205; supplementary table S17, Supplementary Material online).

To explore how the signal for gene flow varied across the genome and test its association with recombination rate, we calculated the probability of alternative topologies and estimated demographic parameters for 100 kb genomic windows. We adapted PipeMaster by adding an additional parameter for intralocus recombination rate to our simulations. This approach yielded high accuracy in model classification (accuracy = 0.9314; categorical cross-entropy = 0.18). We also recovered high correlations between simulated and pseudoobserved data, indicating good accuracy in parameter estimation for *N*_e_ (average *R*^2^ = 0.94; average MAE = 73,099 individuals) and divergence times (average *R*^2^ = 0.87; average MAE = 39,172 ya) but not for gene flow (average *R*^2^ = 0.54; average MAE = 0.19 migrants per generation). Effective population sizes and divergence times varied over 1 order of magnitude, and gene flow varied over 2 orders of magnitude across the genome. The substantial variation in *N*_e_ and gene flow across the genome was positively associated with recombination rate in all 3 species, except gene flow in *L. vociferans* (supplementary fig. S17 and table S19, Supplementary Material online). Variation in divergence time was not associated with recombination rate in any of the species (supplementary table S19, Supplementary Material online).

### Selection Bias Estimates of Demographic Parameters

**Fig. 7.**
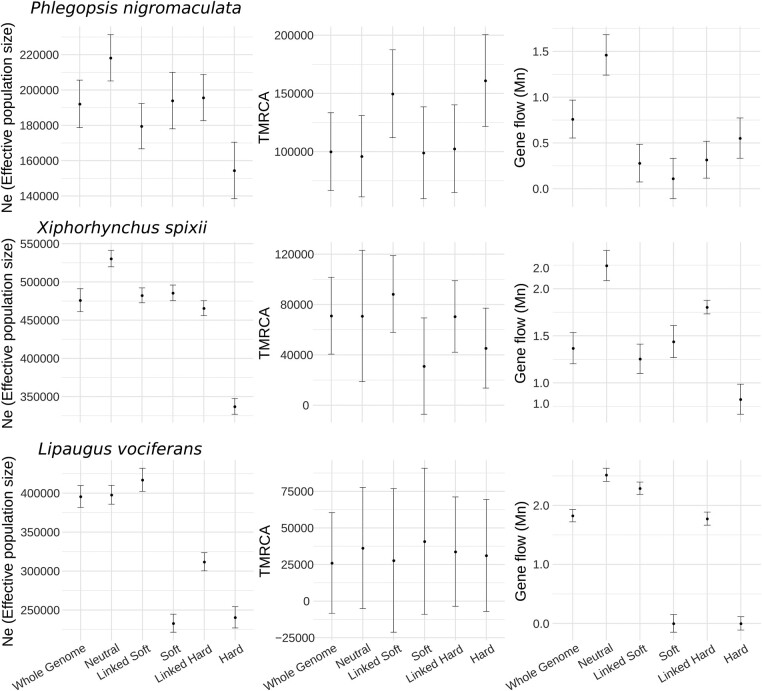
Genomic regions inferred to be evolving neutrally had larger effective population sizes and higher gene flow than areas with signatures of selection. Demographic parameter estimation for the 3 studied species of Amazonian birds. *N*_e_ refers to the effective population size of Tapajos populations. Time to the most recent common ancestor of the most recent divergence event (TMRCA). Gene flow rate between Tapajos and Xingu populations, expressed in migrants per generation (Mn). Classes on the *x* axis represent genome-wide windows (whole genome), and subsets of genomic windows assigned with high probability to distinct models tested with diploS/HIC. Neutral: neutrally evolving windows; Linked Soft: windows linked to a soft sweep; Soft: windows assigned to a soft sweep; Linked Hard: windows linked to a hard sweep; and Hard: windows assigned to a hard sweep.

Our results suggested that regions with reduced recombination and lower genetic diversity might have a better fit to bifurcating phylogenetic models; however, these regions might not be adequate for estimating evolutionary neutral processes such as gene flow due to the more pronounced effect of linked selection. To test if variation in genomic architecture might impact demographic parameter estimation, we explored how selection affected *N*_e_, gene flow, and divergence time estimates. We calculated parameters from subsets of genomic windows classified under distinct selection regimes with diploS/HIC using our window-based machine learning approach. Our results supported higher *N*_e_ and gene flow in neutral areas of the genome than areas under selection, with little to no overlap of standard error distributions (Fig. 7). We also found that genome-wide windows produced more similar estimates for *N*_e_ and gene flow in regions with signatures of linked selection than neutral regions (Fig. 7).

## Discussion

We found that genomic architecture directly impacts phylogenetic inference and population genetic parameters from genome-wide estimates, adding an underappreciated layer of complexity for testing population differentiation hypotheses. Population differentiation inferred from Amazonian taxa exhibits a wide array of temporal and geographic patterns (Smith et al. 2014; Lynch Alfaro et al. 2015; Penz et al. 2015; Thom and Aleixo 2015; de Oliveira et al. 2016; Byrne et al. 2018; Dagosta and De Pinna 2019; Silva et al. 2019; Thom et al. 2020). Our results indicate that the heterogeneity in parameters estimated in these studies may be influenced by unaccounted evolutionary processes. We found that the interplay of selection, gene flow, and recombination shaped the genomic landscape of genetic diversity, resulting in different portions of the genome strongly supporting alternative hypotheses on the geographic differentiation of populations. This study indicates that genome-wide estimates of phylogeny might be misleading, and accounting for the processes that produce a heterogeneous genomic landscape is essential to understanding the spatial–temporal dynamics underpinning genetic structuring across the landscape.

### Genomic Architecture Informs Patterns of Geographic Differentiation

We found that introgression, which is associated with recombination rate, can produce a highly heterogeneous landscape of phylogenetic conflict that obscures inferences on population history. For example, in *P. nigromaculata*, gene flow between nonsister taxa was positively associated with recombination rate, and the topology (topology 2) associated with introgression was found to be the most prevalent tree across the genome. This pattern affected the genome-wide species tree and topology weight analyses. In contrast, the topology reflecting the most probable species tree (topology 1) was more frequent in windows with low recombination rates. The phylogenetic conflict between alternative topologies has practical implications for testing spatial patterns of population differentiation. Support for topology 2 for *P. nigromaculata* would indicate that the taxa diverged via a stepping-stone process from the west through the Tapajos, Xingu, and Belem regions, consistent with the moisture gradient hypothesis (Silva et al. 2019). In contrast, if topology 1 reflects the population history of *P. nigromaculata*, it would indicate the opposite scenario, with an ancestral population in southeastern Amazonia, which could be linked to physiographic changes in the landscape (Albert et al. 2018; Musher et al. 2021). Although the probability of alternative topologies was more stable across the genomes of *X. spixii* and *L. vociferans*, topology weight was also predicted by recombination rate and gene flow. The stronger pattern recovered for *P. nigromaculata* suggests that idiosyncratic, species-specific histories might reflect the extent of the association between genomic architecture and phylogenetic signal. The pattern reported for *P. nigromaculata* might be common across the thousands of lineages isolated by Amazonian tributaries, given that recent studies have been reporting fast radiations and extensive introgression across rivers (Weir et al. 2015; Barrera-Guzmán et al. 2018; Ferreira et al. 2018; Berv et al. 2021; Del-Rio et al. 2021; Musher et al. 2021).

We found that the interaction between intrinsic (e.g. recombination and selection) and extrinsic (e.g. gene flow) genomic processes leads to phylogenetic conflict affecting genome-wide estimates. In the presence of gene flow, low recombination areas are more likely to maintain the ancient branching signal (Li et al. 2019; Tigano et al. 2022). The strong linkage in low recombination regions should lead to the more effective removal of alleles introduced by hybridization that are more likely to be deleterious (Nachman and Payseur 2012; Schumer et al. 2018). In this sense, even methods designed to incorporate gene flow into phylogenetic analyses, such as phylogenetic network approaches (Solís-Lemus and Ané 2016; Wen et al. 2018), might produce misleading results when considering genome-wide markers. Although phylogenetic networks are an ideal way to track the presence of gene flow, it might be difficult to disentangle the processes driving phylogenetic conflict given that introgression proportions might be biased by the genomic landscape.

### Selection and Recombination Effect Parameter Estimates

We showed that pervasive signatures of selection across the genomes of 3 cooccurring species impacted genome-wide demographic parameters. On average and after accounting for false positives, one-third of the genomes of our focal species had a high probability for models with selective sweeps or linked selection. Recent estimations for birds and mammals show substantial variation in the proportion of the genome subject to selection, with approximations above 50% (McVicker et al. 2009; Pouyet et al. 2018; Brand et al. 2021; Manthey et al. 2021). Although our data indicate that regions potentially affected by linked selection have a better fit to a bifurcating phylogenetic model, these regions violate neutral models of evolution, impacting genome-wide population genomic parameters (Schrider et al. 2016; Pouyet et al. 2018). Whole-genome approximations of *N*_e_ and gene flow were more similar to regions with signatures of selection versus regions deemed to be evolving neutrally. For instance, areas of the genome evolving neutrally had up to 13% larger *N*_e_ in *P. nigromaculata* and 64% higher gene flow in *X. spixii* than estimates based on genome-wide loci (Fig. 7). These results are in agreement with studies indicating that demographic parameters can be severely affected by positive and background selection (Schrider et al. 2016; Johri et al. 2021). Although we did not directly model background selection, the large proportion of the genome inferred to be under selection and the fact that the majority of areas with coding sequences were assigned to nonneutral models indicate that sites under negative selection were likely classified under nonneutral models. Simulation studies have reported that background selection might lead to distinct genomic signatures compared to positive selection and hitchhiking (Schrider 2020); however, variation in model parameters might lead to considerable confusion between these processes (Stephan 2010; DeGiorgio et al. 2016). Understanding the relative contribution of both positive and negative selection in shaping levels of genetic diversity across the genome and their specific impacts in phylogenomic approaches may yield greater resolution in identifying the source of phylogenetic conflict and the reliability of specific genomic regions in tree building.

Natural selection can also skew levels of genetic variation in a similar way to certain nonequilibrium demographic histories, often leading to overestimates of population bottlenecks and the rate of demographic expansions (Ewing and Jensen 2016; Schrider et al. 2016). For example, positive selection leading to fixation of large haplotypes linked to the target of selection may mimic population bottlenecks (Wayne and Simonsen 1998), and the recovery from these sweeps might inflate the proportion of rare variants, resembling recent population expansions (Schrider et al. 2016). Although we did not model demographic changes over time, summary statistics that are indicative of demographic oscillations, such as Tajima's *D*, varied considerably across the genome, with more negative values in regions of low recombination (Fig. 2). Our examination of 3 codistributed species showed that the effect of selection on demographic analyses is a general phenomenon that can have a profound effect on modeling population histories.

Reconciling phylogenetic inference and demographic parameter estimation from whole genomes is aided by recombination and selection-aware approaches. By characterizing the genomic landscape, the effect of selection in demographic parameter estimations can be mitigated by targeting genomic regions as distant as possible from potential targets of selection, such as genes and functional elements, as well as avoiding areas of low recombination or affected by biased gene conversion (Pouyet et al. 2018). A key problem with selecting loci with distinct characteristics is that current methods designed to calculate recombination and selection across the genome achieve optimal performance when the demographic history of a population is known (Dapper and Payseur 2018; Harris et al. 2018; Rousselle et al. 2018; Johri et al. 2020). On the other hand, demographic parameters may be heavily biased when recombination and selection are neglected (Ewing and Jensen 2016; Pouyet et al. 2018). On top of that, the historical demography of populations as estimated by coalescent methods (e.g. SMC++) might also be impacted by other unaccounted factors such as historical variation in population structure and gene flow from unsampled populations (Chikhi et al. 2010; Heller et al. 2013; Mazet et al. 2015). The surprisingly high effective population sizes in our focal species inferred from our SMC++ analyses could be a direct effect of these unaccounted processes (supplementary fig. S11, Supplementary Material online). This complex interaction of processes indicates that methods designed to simultaneously account for multiple genomic characteristics, such as recombination, selection, drift, and mutation (Johri et al. 2020; Barroso and Dutheil 2021; Johri et al. 2021), and associated with simulation studies (Johri et al. 2022; Tigano et al. 2022) might be necessary to obtain less biased evolutionary parameters from genome-wide variation.

### Heterogeneous Genomic Landscapes within and between Species

The recombination rate and the proportion of the genome impacted by selection varied considerably among species, leading to different levels of association between genomic architecture and population history. Chromosome size was a good predictor of genetic diversity, recombination rate, and phylogenetic signal in 2 of the species but not in *L. vociferans*. The lack of association between chromosome size and genomic characteristics in *L. vociferans* was unexpected given that during meiosis, chromosome segregation often requires at least 1 recombination event per homologous chromosome pair (Fledel-Alon et al. 2009). This process should lead to an overall higher recombination rate in shorter chromosomes (Kaback et al. 1992; Farré et al. 2012; Kawakami et al. 2014; Haenel et al. 2018; Manthey et al. 2021). Similar results to the ones we obtained for *L. vociferans* have also been observed in other species of birds and mammals (Pessia et al. 2012; Dutoit, Burri, et al. 2017; Kartje et al. 2020) and could be explained by a reduced synteny between our reference and the zebra finch genome or the historical demography of the species, which in some cases can reverse the expected associations between recombination, genetic diversity, and chromosome size (Van Belleghem et al. 2018; Tigano et al. 2022). However, the former scenario is less likely due to the relative stability of chromosomes across avian species, as reported here and elsewhere (Ellegren 2010; Coelho et al. 2019; Peñalba et al. 2020). These results suggest that this was not the case in *L. vociferans*, although genomic architecture seems to be a strong predictor for phylogenetic relationships in the presence of gene flow. It is important to note that the strong positive association between genetic diversity and recombination rate that we obtained likely overrepresented the effects of linked selection across the genome. Population-based methods for estimating recombination, such as ReLERNN, might be affected not only by recombination rate (*r*) but also by variation in effective population size and selection (Adrion et al. 2020). However, the reduced association between recombination rate and genetic diversity in neutral regions of the genome and the significant association between recombination rate and gene flow that we reported indicated that linked selection was an important process in shaping population differentiation and levels of genetic diversity across the genome.

Genomic architecture was likely stable over the timeframe of our study, indicating that a conserved genomic landscape might produce similar patterns of associations with demographic and phylogenetic histories in closely related populations (Dutoit, Vijay, et al. 2017; Van Doren et al. 2017; Vijay et al. 2017; Delmore et al. 2018; Tigano et al. 2022). For example, consistent variation in *F*_ST_ values across the genomes of different population pairs of the same species likely reflects the genomic landscape of the ancestral population. Although the temporal scale on which the genomic architecture remains conserved between diverging lineages is still unknown, our data suggest that regions of low recombination rates and small *N*_e_ in the parent population would promote faster differentiation between daughter populations after isolation.

### Multiple Processes Produce Heterogeneous Landscapes across the Genome

Identifying the driving forces shaping patterns of diversity along the genome of nonmodel organisms and understanding how theoretical models extend to natural systems is a major endeavor in speciation genomics (Comeron 2014; Elyashiv et al. 2016; Stankowski et al. 2019; Barroso and Dutheil 2021). In this study, we demonstrated that the interplay between recombination and selection had a strong impact on phylogenetic inference and demographic parameters, which are key for distinguishing alternative spatial models of divergence. However, other genomic processes might be shaping phylogenetic signals and demographic parameters across the genome. For instance, levels of polymorphism could be associated with variation in mutation rate (Jónsson et al. 2018; Smith et al. 2018; Besenbacher et al. 2019; Barroso and Dutheil 2021). Noncrossover gene conversion (Korunes and Noor 2017), where DNA strands break during meiosis and are repaired based on homologous sequences without crossing-over, and crossover events could be mutagenic, leading to higher mutation rates in areas of higher recombination (Arbeithuber et al. 2015; Korunes and Noor 2017). Simulation studies have rejected gene conversion as a process driving genome-wide patterns of genomic diversity in relatively recent divergence events (Tigano et al. 2022), and empirical studies suggest that mutations associated with crossover events occur at relatively low frequencies (Halldorsson et al. 2019). Although differential mutation rates across the genome might not explain the strong association between genetic diversity and genomic architecture, the majority of the variation in genetic diversity in our focal species was not explained by recombination. This suggests that variation in mutation rate, not associated with recombination, could be playing a role in the genomic landscape of genetic diversity. It is important to note that irrespective of the processes driving the heterogeneous levels of genetic variation across the genome, these biases on genome-wide phylogenetic and population genetics inferences may remain unless the multitude of parameters varying across the genome are modeled in a unifying approach (Johri et al. 2021, 2022). Until that horizon is reached, genomic architecture-aware approaches can be used to disentangle the effects of intrinsic genomic characteristics and selection from neutral processes to better inform how species diversify across the landscape.

## Materials and Methods

### Studied Species, Sampling Design, and Whole-Genome Sequencing

We selected 3 species that occur in southeastern Amazonia and occur in distinct forest strata of upland forest habitats: (i) *P. nigromaculata*, an obligatory army-ant follower restricted to the understory, with 3 distinct subspecies that are genetically structured and isolated by the Xingu and Tocantins Rivers (Aleixo et al. 2009); (ii) *X. spixii*, which occupies the midstory of eastern Amazonian forests and has 2 structured populations divided by the Xingu River (Aleixo 2004); and (iii) *L. vociferans*, a widespread canopy species that is expected to be less structured across rivers.

To optimize the spatial representation of our samples, we selected a single individual per locality, targeting approximately 10 individuals per interfluve per species (Tapajos, Xingu, and Belem), yielding a total of 33, 33, and 30 samples for *P. nigromaculata*, *L. vociferans*, and *X. spixii*, respectively (supplementary table S1, Supplementary Material online; Fig. 1). We isolated genomic DNA from muscle tissue preserved in alcohol (*n* = 65) and skin from the toe pads of museum specimens (*n* = 31). The proportion of tissue and toe pad samples varied between species (supplementary table S1, Supplementary Material online). All samples were loaned from the Museu Paraense Emilio Goeldi (MPEG). From tissues, we extracted DNA with a Qiagen high molecular weight DNA kit (MagAttract HMW DNA Kit, Qiagen). For the toe pads, we performed a protocol specific for degraded DNA consisting of additional steps for washing the samples with H_2_O and EtOH prior to extracting and extra time for digestion. We modified the DNeasy extraction protocol (DNeasy Blood & Tissue Kits, Qiagen) by replacing the standard spin columns with the QIAquick PCR filter columns (QIAquick PCR Purification Kit, Qiagen), selecting for smaller fragments of DNA, typically found in degraded samples. Toe pad extractions were conducted in a dedicated lab for working with historical samples at the American Museum of Natural History (AMNH) to reduce contamination risk. We quantified DNA extracts using a Qubit 2.0 Fluorometer (Thermo Fisher Scientific). Illumina libraries with variable insert sizes were generated, and samples were sequenced by Rapid Genomics (Gainesville, Florida) to ∼10× coverage using 3.5 lanes of paired-end (2 × 150 bp) Illumina S4 NovaSeq 6000. Given the short insert size obtained for historical samples due to DNA degradation, we used 2 full lanes for sequencing 31 toe pad samples (supplementary table S1, Supplementary Material online). The larger sequencing output for historical samples allowed us to obtain similar coverage between distinct tissue types after bioinformatic filtering. Raw reads were trimmed and filtered using Trimmomatic v0.36 (Bolger et al. 2014).

### Reference Genomes and Gene Annotations

We obtained reference genomes from closely related species. For *P. nigromaculata*, we used as a reference the genome of *R. melanosticta* (Coelho et al. 2019) with time to the most recent common ancestor (TMRCA) = 9.60 Ma (Harvey et al. 2020). For *X. spixii*, we used the genome of *X. elegans* (GCA_013401175.1 ASM1340117v1; NCBI genome ID: 92877; scaffold N50: 172,271 bp; number of scaffolds: 89,575; Feng et al. 2020) with TMRCA = 2.36 Ma (Harvey et al. 2020), and for *L. vociferans*, we used the genome of *C. ornatus* (GCA_013396775.1 ASM1339677v1; NCBI genome ID: 92752; scaffold N50: 292,884 bp; number of scaffolds: 26,262; Feng et al. 2020) with TMRCA = 15.10 Ma (Harvey et al. 2020). Given that passerine bird chromosomes are known to have high synteny and evolutionary stasis between distantly related species (Ellegren 2010; Coelho et al. 2019; Peñalba et al. 2020), we produced a pseudochromosome reference genome for *X. elegans* and *C. ornatus* by ordering and orienting their scaffolds to the chromosomes of the zebra finch (*T. guttata*; version taeGut3.2.4) with chromosemble in Satsuma v3.1.0 (Grabherr et al. 2010). For *R. melanosticta*, we used the chromosome assignment conducted in a previous study (Coelho et al. 2019). To check the completeness of our pseudochromosome references, we used BUSCO v2.0.1 (Waterhouse et al. 2018) to search for a set of single-copy avian ortholog loci. To transfer the original genome annotations from the scaffold assemblies to the pseudochromosome reference genomes, we mapped the genomic coordinates of each annotated feature using gmap (Wu and Watanabe 2005). For *R. melanosticta*, we used the annotation performed by Mikkelsen and Weir (2020), and for *X. elegans* and *L. vociferans*, we used distinct genome annotations performed by Feng et al. (2020). A total of 98.90% (15,195 genes), 97.46% (14,834), and 98.92% (15,599) of all annotated genes in *R. melanosticta*, *X. elegans*, and *C. ornatus* were successfully mapped to the pseudochromosome reference, respectively.

We downloaded raw reads from additional closely related species that were used as outgroups in phylogenetic analyses. For *P. nigromaculata*, we included *R. melanosticta*, *Sakesphorus luctuosus* (GCA_013396695.1 ASM1339669v1; NCBI genome ID: 92896; Feng et al. 2020), and *X. elegans* as outgroups. For *X. spixii*, we included *X. elegans*, *S. luctuosus*, *Campylorhamphus procurvoides* (GCA_013396655.1 ASM1339665v1; NCBI genome ID: 92894; Feng et al. 2020), and *Furnarius figulus* (GCA_013397465.1 ASM1339746v1; NCBI genome ID: 92763; Feng et al. 2020). For *L. vociferans*, we included *C. ornatus*, *Pachyramphus minor* (GCA_013397135.1 ASM1339713v1; NCBI genome ID: 92755; Feng et al. 2020), and *Tyrannus savana* (GCA_013399735.1 ASM1339973v1; NCBI genome ID: 92814; Feng et al. 2020).

### Read Alignment, Variant Calling, and Filtering

Trimmed and filtered reads were aligned to the references in BWA v0.7.17 (Li and Durbin 2009) using default parameters. We used Picard v.2.0.1 (Broad Institute, Cambridge, Massachusetts; http://broadinstitute.github.io/picard/) to (i) sort sam files with SortSam; (ii) reassign reads to groups with AddOrReplaceReadGroups; (iii) identify duplicated reads with Markduplicates; (iv) calculate summary statistics with CollectAlignmentSummaryMetrics, CollectInsertSizeMetrics, and CollectRawWgsMetrics; and (v) create indexes with BuildBamIndex. All Picard functions were run with default parameters. We used the standard GATK v3.8 (McKenna et al. 2010) pipeline to (i) call SNPs and Indels for each individual separately with HaplotypeCaller; (ii) perform genotyping with GenotypeGVCFs, assuming a value of 0.05 for the --heterozygosity flag; and (iii) flag and filter variants with VariantFiltration. Given the lack of a high confidence SNP panel, we implemented hard filtering options recommended by the Broad Institute’s Best Practices (https://gatk.broadinstitute.org/). We filtered SNPs with quality by depth below 2 (QD < 2.0), SNPs where reads containing the alternative allele were considerably shorter than reads with the reference allele (ReadPosRankSum < −8), SNPs with root mean square of the mapping quality lower than 40 (MQ < 40.0), SNPs with evidence of strand bias (FS > 60.0 and SOR > 3.0), and SNPs where the read with the alternative allele had a lower mapping quality than the reference allele (MQRankSumTest < −12.5). Lastly, we filtered raw VCF files by keeping only biallelic sites, with no more than 50% of missing information, with a minimum read depth of 4 and maximum of 30, and read quality score >Q20 using VCFTOOLS v0.1.15 (Danecek et al. 2011). We phased the genotypes in our genomic VCF files using BEAGLE v5.1 (Browning and Browning 2007) in sliding windows of 10 kb and overlapped between windows of 1 kb.

### Window-Based Summary Statistics and Population Structure

We tested whether genetic diversity, population differentiation, and phylogenetic relationships were associated with intrinsic genomic characteristics (i.e. recombination rate and chromosome size) and selection using a sliding window and model-based approach. To summarize the genomic landscape of differentiation and genetic diversity within the 3 studied species, we calculated *F*_ST_, *D*_XY_, nucleotide diversity (*π*), Patterson's *D* statistic (ABBA/BABA), and *f*_dm_ (Malinsky et al. 2015) using scripts available at https://github.com/simonhmartin/genomics_general (Martin et al. 2014). We initially converted VCF files per species into geno format using parseVCF.py, and calculations were performed for the different populations in each of the 3 interfluves using popgenWindows.py. We used phased VCF files, setting the window size to 100 kb (-w option) without overlap between windows and the minimum number of sites without missing information per window to 500 (-m option). To obtain GC content proportion across 100 kb windows for our reference genomes, we used sequir v4.2 (Charif and Lobry 2007) in R (R Core Team 2021).

Next, to characterize how genome-wide patterns of genetic structure were distributed across rivers, we performed PCA and individual relatedness analyses based on identity by descent using SNPRelate v1.20.1 (Zheng et al. 2012) in R. In order to minimize the effect of missing genotypes in the PCA, we filtered our VCF files to keep SNPs present in at least 70% of the individuals. We also used SNPRelate to perform an identity-by-state (IBS) analysis among individuals for each species. To avoid the influence of SNP clusters in our PCA and IBS analyses, we pruned SNPs in approximate linkage equilibrium (LD > 0.2) with each other.

Patterns of population structuring might differ along the genome due to variation in recombination rates, selection, mutation, and drift (Ellegren et al. 2012; Langley et al. 2012; Li et al. 2019). To test if distinct regions of the genome have contrasting patterns of population structure, we used Lostruct (Li and Ralph 2019). This approach (i) summarizes the relatedness between individuals across genomic windows using local PCAs; (ii) calculates the pairwise dissimilarity among windows; (iii) uses MDS to produce a visualization of how variable patterns of relatedness are across the genome; and (iv) allows the user to combine regions by similarity to inspect contrasting patterns of genetic structure across the genome. We ran Lostruct for windows with 1,000 SNPs, allowing for 30% of missing genotypes. To visualize the results, we selected 10% of the windows closer to the 3 further points on the 2 first MDS coordinates and performed individual PCA analysis on clustered windows.

### Phylogenomic Analyses and Topology Weighting

After characterizing the genomic landscape of genetic diversity and differentiation among the populations, we explored how phylogenetic relationships vary between species and across the genome. These analyses combined allowed us to test whether genetic diversity metrics, population differentiation, gene flow, and phylogenetic signal could be predicted by recombination rate and chromosome size, and whether areas of the genome with reduced genetic diversity and lower introgression levels were more likely to maintain phylogenetic signal supporting the species tree.

First, we estimated genome-wide phylogenies for each species in IQTREE-2 v2.1.5 (Nguyen et al. 2015; Minh et al. 2020) by concatenating SNPs and controlling for ascertainment bias (Fig. 1). We converted VCF files to PHYLIP format using vcf2phylip.py (Ortiz 2019), randomly resolving heterozygous genotypes, and keeping SNPs present in at least 80% of the individuals. In IQTREE2, we ran a total of 1,000 rapid bootstrap replicates and controlled for ascertainment bias by assuming a GTR + ASC substitution model.

To estimate the posterior probability of unrooted species trees, we used Astral-III v5.1.1 (Zhang et al. 2018; Rabiee et al. 2019). To obtain phylogenetic trees based on sliding windows of phased VCF files, we used PHYML v3.0 (Guindon et al. 2010) following Martin and Van Belleghem (2017). We tested windows with different amounts of information content, selecting regions with 50, 100, 500, and 1,000 SNPs. We opted to use windows with a fixed number of SNPs instead of fixed alignment length to homogenize the amount of informative sites between windows. We conducted 100 bootstrap replicates per window. Given that windows with different information content yielded similar results, we only present the results for 100 SNP windows (with an average window size of 14,503, 15,637, and 5,821 bp for *P. nigromaculata*, *X. spixii*, and *L. vociferans*, respectively) in subsequent analyses. We used Astral to score unrooted trees (-q option) by calculating their quartet score, branch lengths, and branch support. We set our main topology (outgroup, Belem [Xingu, Tapajos]) and used the -t 2 option to calculate the same metrics for the first and second alternative topologies. Given we only have 4 terminals per lineage (3 populations + outgroup), there are 3 possible unrooted trees. Therefore, this approach allowed us to calculate the posterior probability of all possible topologies. We conducted this approach for the whole set of gene trees and also for subsets of the data, based on specific characteristics of each window. To assess how support for a specific topology varies based on thresholds for specific summary statistics, we selected windows across the genome with the upper and lower 10% tile for recombination rate, *F*_ST_, *π*, *D*_XY_, and *D* statistics.

To obtain a continuous variable describing how phylogenetic signal varied across genomic windows, we calculated unrooted topology weights using Twisst (Martin and Van Belleghem 2017). This approach allowed us to quantify the relationships among populations for each genomic window, providing a direct way to test how phylogenetic signal varied with recombination rate and chromosome size.

### Population Genetic Estimate of Recombination Rate

We tested whether recombination rate was associated with population differentiation and topological relationships across the genome (Li et al. 2019; Manthey et al. 2021). To estimate recombination rate (*r* = recombination rate per base pair per generation) from population-level data for each of the species complexes, we used ReLERNN (Adrion et al. 2020). This approach approximates the genomic landscape of recombination by leveraging recurrent neural networks using the raw genotype matrix as a feature vector, avoiding the need to convert the data into summary statistics. We used ReLERNN according to Adrion et al. (2020) by performing 100,000 coalescent simulations based on the population genetic properties of the empirical data. Simulations are then used to train, test, and validate a recurrent neural network model designed to predict the per-base recombination rate across sliding windows of the genome. The fully trained recurrent neural network was then used to predict the recombination landscape across the genomes of the targeted populations. Given that genetic structure could potentially influence ReLERNN results (Mezmouk et al. 2011; Mangin et al. 2012), we restricted our analyses to the Tapajos population, which was composed exclusively of modern tissue samples, and we did not find any sign of spatial genetic substructure in the area for all 3 species (see below). Although we did not calculate *r* for all populations, the landscape of recombination across bird lineages is considered conserved, and variation between recently diverged populations should be minimal (Singhal et al. 2015). To account for the historical demography of the populations, we provided to ReLERNN the output of our SMC++ analyses (see below) with the --demographicHistory option. To account for uncertainty in the prediction of the recombination rate, we performed 5 replicates with the ReLERNN_BSCORRECT function. We assessed the accuracy of our estimates by evaluating the *R*^2^ and MAE of general linear models between predicted and simulated rates for 1,000 test examples. We considered a mutation rate of 2.42 × 10^−9^ mutations per generation for all 3 species based on *Picoides pubescens* (Jarvis et al. 2014; Zhang et al. 2014; Nadachowska-Brzyska et al. 2015), and a 1-yr generation time (Willis 1967, 1978, 1981). The mutation rate we used is in line with the estimates for multiple passerine birds such as *Certhia americana* (2.506 × 10^−9^; Manthey et al. 2021), *T. guttata* (2.21 × 10^−9^, Nam et al. 2010), and *Manacus vitellinus* (3.15 × 10^−9^; Nadachowska-Brzyska et al. 2015).

### Testing for Associations across the Genomic Landscape

After characterizing the genomic landscape of 3 codistributed birds in southeastern Amazonia, we tested for associations between window-based summary statistics, recombination rate, and chromosome size and identified which variables best predicted gene tree heterogeneity, gene flow, population structuring, and genetic diversity across the genome. All window-based estimates were combined into 100 kb genomic windows. For variables calculated based on a fixed number of SNPs (e.g. topology weight and Lostruct) and the recombination rate obtained with ReLERNN that produced windows of variable sizes but smaller than 100 kb, we calculated a weighted average among windows matching each 100 kb window. We fit general linear regressions and Pearson's correlation index between population genetics summary statistics, demographic parameters, phylogenetic weights, recombination rate, and chromosome size in R. To account for the potential nonlinearity of these relationships, we also fit a LOESS model using the R package caret (Kuhn 2008). Models were trained using leave-one-out cross-validation from 80% of the total data.

### Testing for Biases Associated with Chromosome Synteny

Chromosome rearrangements, fission, and fusion might affect the overall recombination rate of a genomic window and also the chromosome assignment of the reference genome's scaffolds in our pseudochromosome approach. To investigate if differences in chromosomal structure among species could be driving biased associations between genomic architecture and demographic and phylogenetic parameters, we aligned the pseudochromosomes of *R. melanosticta* (Coelho et al. 2019) to the chromosomes of *Chiroxiphia lanceolata* (bChiLan1.pri; SAMN12620979) and reran correlations using only syntenic genomic regions located on homologous chromosomes. The genome of *C. lanceolata* is one of the highest quality genomes available for a suboscine passerine, which is the suborder of our targeted species, and conserved regions between these taxa will likely be stable in all target species. We performed whole-genome alignment using ProgressiveCactus with default parameters (Armstrong et al. 2020). Synteny analysis was conducted in halSynteny (Krasheninnikova et al. 2020), using --maxAnchorDistance 100000 and --minBlockSize 100000.

### Inferring Selection across the Genome

Although a positive association between recombination rate and genetic diversity suggests that linked selection has a significant role in shaping levels of genetic diversity across the genome, it does not indicate which portions of the genome are directly impacted by this process. By explicitly modeling selection across the genome, we were able to infer if areas that are more impacted by linked selection, despite being less affected by gene flow and having a better fit to bifurcating phylogenetic models, could yield biased demographic parameters by violating the assumption of neutral evolution. We estimated the effects of selection and linked selection across the genome by modeling selective sweeps (positive selection) and neutral variation linked to selective sweeps using a machine learning approach implemented on diploS/HIC (Kern and Schrider 2018). This method uses coalescent simulations of genomic windows to train and test a CNN designed to predict hard and soft selective sweeps and genetic variation linked to selective sweeps across sliding windows of the genome. We ran this analysis only on Tapajos populations, which contained exclusively high-quality tissue samples. Genomic windows were simulated using discoal (Kern and Schrider 2016) according to 5 distinct models: (i) hard selective sweep; (ii) soft selective sweep; (iii) neutral variation linked to soft selective sweep; (iv) neutral variation linked to hard selective sweep; and (v) neutral genetic variation. We performed 5,000 simulations per model using 220 kb genomic windows divided into 11 subwindows of 20 kb. To account for the neutral demography of the populations, which is essential to obtain robust model classification between windows (Harris et al. 2018), we modeled population sizes through time using unphased genomes in SMC++ v1.15.3 (Terhorst et al. 2017). Our goal with this approach was to track past fluctuations in *N*_e_ to be included both in ReLERNN (Adrion et al. 2020) and discoal simulations (Kern and Schrider 2016). We ran SMC++ exclusively for the Tapajos population of each species, assuming the mutation rate and generation time described above. We explored the historical demography of populations within a time window between the present and 300,000 yr ago.

To account for uncertainty in simulated parameters, we followed the approach of Manthey et al. (2021) by allowing current *N*_e_ to vary between 1/3 and 3× the value obtained with SMC++ within a uniform distribution. Priors for the population-scaled recombination rate (*rho* = 4*N*_e_*r*; where *r* is the recombination rate estimated with ReLERNN) were set based on the minimum and maximum values obtained across windows with ReLERNN. We set a uniform prior for selection coefficients ranging from 0.00025 to 0.025, and we conditioned sweep completion between the present and 10,000 generations ago. We used a uniform prior between 0.01 and 0.2 for the initial frequency of adaptive variants in soft sweep models. Simulations were converted into feature vectors consisting of population genetic summary statistics, taking into account the observed amount of missing data by using a genomic mask. We calculated the probability of alternative models for the observed 20 kb windows. We ran CNNs for 1,000 epochs, stopping the run if validation accuracy did not improve for 50 consecutive epochs. We ran 5 independent runs and predicted the observed data with the run that provided the highest accuracy on testing data. To assess the classification power of the CNNs, we inspected the overall accuracy, the FPR, recall (the number of correct positive predictions made out of all positive predictions that could have been made), and area under the curve (AUC). To acknowledge the uncertainty in model selection, we only assigned a model with a probability higher than 70% to a genomic window. Selective sweeps, both soft and hard, are expected to produce long tracks of homozygosity near the site under selection. To further validate the results obtained with diploS/HIC, we estimated *H* statistics using H-scan (Schlamp et al. 2016). This approach allowed us to test if regions classified as selective sweeps by diploS/HIC had longer homozygosity tracts than neutral regions. We measured haplotype homozygosity tract lengths based on physical distance (in number of base pairs, -d 0) and a maximum gap length between neighboring SNPs of 100 kb (-g 100,000).

To explore if areas putatively impacted by background selection were more likely assigned to nonneutral models by diploS/HIC, we tested if the proportion of coding sequences could predict if a window was classified as neutral or nonneutral (i.e. selective sweep or linked to selective sweep) by diploS/HIC. In addition, we calculated the proportion of exons that were found on windows assigned to these 2 categories. We used a random forest classification model using RandomForest in R with default parameters. The proportion of exons was calculated based on 100 kb windows.

### Model-Based Approach to Account for Recombination and Selection

Because the methods we used to infer gene and species trees do not incorporate gene flow and we expected introgression to occur across rivers, we employed an additional model-based approach to test the likelihood of alternative topologies while accounting for introgression. Specifically, we used coalescent simulations that modeled demographic history, including gene flow, and performed model selection using supervised machine learning. We simulated data under 3 alternative topologies that reflected different geographic arrangements of rivers: topology 1 (out [Belem {Xingu, Tapajos}]); topology 2 (out [Tapajos {Xingu, Belem}]); and topology 3 (out [Xingu {Tapajos, Belem}]). We allowed for constant gene flow after the divergence between Xingu and Belem, and Xingu and Tapajos populations. We did not allow gene flow between Belem and Tapajos due to the geographic disjunction between these populations. We simulated 5,000 loci of 10 kb, using uniform and wide priors for all parameters (supplementary table S20, Supplementary Material online), and performed 1 million simulations per model assuming the same mutation rate described previously. Genetic data for each model were simulated in PipeMaster (Gehara et al. 2017), which allows for a user-friendly implementation of msABC (Pavlidis et al. 2010). To align loci across individuals, phased VCF files per population were split every 10 kb window and converted into fasta format, including monomorphic sites, using bcftools (Li 2011). Fasta alignments were converted into feature vectors with PipeMaster, which uses PopGenome (Pfeifer et al. 2014) in R. To obtain a genome-wide estimate of demographic parameters, we selected 1 10 kb genomic window every 100 kb to reduce the effect of linkage between windows. This procedure yielded a total of 7,213, 9,140, and 9,693 windows for *P. nigromaculata*, *X. spixii*, and *L. vociferans*, respectively. We randomly subsampled 5,000 windows from these data sets. We summarized the genetic variation of observed and simulated data in feature vectors composed of the mean and variance of the following summary statistics across loci: number of segregating sites per population and summed across populations; nucleotide diversity per population and for all populations combined; Watterson's theta (Watterson 1975) per population and for all populations combined; pairwise *F*_ST_ between populations; number of shared alleles between pairs of populations; number of private alleles per population and between pairs of populations; and number of fixed alleles per population and between pairs of populations. We explored how simulated models fit the observed data using PCAs by plotting the first 4 PCs of simulated statistics versus observed. We also generated goodness-of-fit plots using the gfit function of abc v2.1 (Csilléry et al. 2012) in R.

To classify observed data sets into our 3 models, we used a neural network (nnet) implemented in Keras v2.3 (Arnold 2017; https://github.com/rstudio/keras) in R. After an initial exploration for the best architecture for our nnet, we conducted our final analyses using 3 hidden layers with 32 internal nodes and a “relu” activation function. The output layer was composed of 3 nodes and a “softmax” activation function. A quarter (25%) of the simulations were used as testing data. We ran the training step for 1,000 epochs using “adam” optimizer and a batch size of 20,000. A small percentage (5%) of the training data set was used for validation. We used the overall accuracy and the sparse_categorical_crossentropy to track improvements in model classification. For the most probable model considering genome-wide windows per species, we estimated demographic parameters with a nnet with similar architecture but designed to predict continuous variables. For this step, we used an output layer with a single node and a “relu” activation. In the training step, we used the mean absolute percentage error (MAE) as an optimizer, training the nnet for 3,000 epochs with a batch size of 10,000 and a validation split of 0.1. We ran this procedure 10 times for each demographic parameter and summarized the results by calculating the mean across replicates. To additionally assess the accuracy of parameter estimation, we calculated the coefficient of correlation between estimated and true simulated values of the testing data set.

To explore how genome-wide parameters differed from the ones estimated for regions with distinct signatures of selection and under neutrality, we created subsets of 10 kb windows that were assigned with high probability (>70%) to 1 of the 5 distinct models implemented in diploS/HIC. For each species, we estimated parameters based on the best model considering genome-wide windows. We selected up to 1,000 windows for each of the 5 selection classes and performed the same approach as described above.

Lastly, to explore the associations between demographic parameters and recombination rate, we calculated the probability of alternative topologies and estimated demographic parameters for 100 kb genomic windows, taking intralocus recombination into account. We used a similar approach as described above, but simulations and observed data consisted of individual 100 kb windows. To model intralocus recombination, we used a modified version of PipeMaster that included an additional parameter for recombination rate. By selecting a larger window size than the genome-wide approach, we increased the information content and resolution of summary statistics for single genomic windows. We performed 100,000 simulations per model and used the same uniform priors for all parameters as implemented above. For intralocus recombination, we set a uniform prior ranging from 0 to the maximum value obtained with ReLERNN per species (*P. nigromaculata* = 3.021 × 10^−9^; *X. spixii* = 2.475 × 10^−9^; *L. vociferans* = 2.171 × 10^−9^).

### Simulating the Effect of Recombination Rate and Gene Flow on Topology Weight

To better understand how recombination rate and gene flow impact topology weight, we performed coalescent simulations based on demographic parameters obtained for *P. nigromaculata* and calculated topology weights using Twisst (Martin and Van Belleghem 2017). We simulated 1,000 windows of 10 kb for 4 models, varying the presence of intralocus recombination and gene flow between Xingu and Belem, assuming topology 1 (3 ingroups plus 1 outgroup). Simulated parameters are available in supplementary table S21, Supplementary Material online. Simulations were performed with PipeMaster, and we converted the ms output to PHYLIP format with PopGenome. We ran trees for each 10 kb window with IQTREE-2 using default parameters and ran Twisst on this estimated set of trees.

## Supplementary Material

evae002_Supplementary_Data

## Data Availability

The raw genetic data underlying this article are available in NCBI Short Read Archive BioProject PRJNA1061539 (http://www.ncbi.nlm.nih.gov/bioproject/1061539). All code and source data sets needed to replicate this study are available in Dryad (10.5061/dryad.76hdr7t2s) and on GitHub (https://github.com/GregoryThom/Genomic-architecture-Amazonian-birds).

## References

[evae002-B1] Adrion JR , Galloway JG, Kern AD. Predicting the landscape of recombination using deep learning. Mol Biol Evol. 2020:37(6):1790–1808. 10.1093/molbev/msaa038. 32077950 PMC7253213

[evae002-B2] Albert JS , CraigAlbert JS, Craig JM, Tagliacollo VA, Petry P. Upland and lowland fishes: a test of the River Capture Hypothesis. In: Hoorn CM, Perrigo A, Antonelli A, editors. Mountains, climate and biodiversity. Cambridge: Wiley Press; 2018. p. 273–294.

[evae002-B3] Aleixo A . Historical diversification of a terra-firme forest bird superspecies: a phylogeographic perspective on the role of different hypotheses of Amazonian diversification. Evolution 2004:58(6):1303–1317. 10.1111/j.0014-3820.2004.tb01709.x. 15266979

[evae002-B4] Aleixo A , Burlamaqui TCT, Schneider MPC, Gonçalves EC. Molecular systematics and plumage evolution in the monotypic obligate army-ant-following genus *Skutchia* (Thamnophilidae). Condor. 2009:111(2):382–387. 10.1525/cond.2009.080097.

[evae002-B5] Arbeithuber B , Betancourt AJ, Ebner T, Tiemann-Boege I. Crossovers are associated with mutation and biased gene conversion at recombination hotspots. Proc Natl Acad Sci U S A. 2015:112(7):2109–2114. 10.1073/pnas.1416622112. 25646453 PMC4343121

[evae002-B6] Armstrong J , Hickey G, Diekhans M, Fiddes IT, Novak AM, Deran A, Fang Q, Xie D, Feng S, Stiller J, et al Progressive Cactus is a multiple-genome aligner for the thousand-genome era. Nature 2020:587(7833):246–251. 10.1038/s41586-020-2871-y. 33177663 PMC7673649

[evae002-B7] Arnold TB . Kerasr: R interface to the Keras deep learning library. J Open Source Software. 2017:2(14):296. 10.21105/joss.00296.

[evae002-B8] Barrera-Guzmán AO , Aleixo A, Shawkey MD, Weir JT. Hybrid speciation leads to novel male secondary sexual ornamentation of an Amazonian bird. Proc Natl Acad Sci USA. 2018:115(2):E218–E225. 10.1073/pnas.1717319115. 29279398 PMC5777072

[evae002-B9] Barroso GV , Dutheil JY. 2021. Mutation rate variation shapes genome-wide diversity in Drosophila melanogaster. bioRxiv 460667. 10.1101/2021.09.16.460667, 16 September 2021, preprint: not peer reviewed.

[evae002-B10] Berv JS , Campagna L, Feo TJ, Castro-Astor I, Ribas CC, Prum RO, Lovette IJ. Genomic phylogeography of the White-crowned Manakin *Pseudopipra pipra* (Aves: Pipridae) illuminates a continental-scale radiation out of the Andes. Mol Phylogenet Evol. 2021:164:107205. 10.1016/j.ympev.2021.107205. 34015448

[evae002-B11] Besenbacher S , Hvilsom C, Marques-Bonet T, Mailund T, Schierup MH. Direct estimation of mutations in great apes reconciles phylogenetic dating. Nat Ecol Evol. 2019:3(2):286–292. 10.1038/s41559-018-0778-x. 30664699

[evae002-B12] Bolger AM , Lohse M, Usadel B. Trimmomatic: a flexible trimmer for Illumina sequence data. Bioinformatics 2014:30(15):2114–2120. 10.1093/bioinformatics/btu170. 24695404 PMC4103590

[evae002-B13] Brand CM , White FJ, Ting N, Webster TH. 2021. Soft sweeps predominate recent positive selection in bonobos (*Pan paniscus*) and chimpanzees (*Pan troglodytes*). bioRxiv 422788. 10.1101/2020.12.14.422788, 15 December 2020, preprint: not peer reviewed.

[evae002-B14] Brandvain Y , Kenney AM, Flagel L, Coop G, Sweigart AL. Speciation and introgression between *Mimulus nasutus* and *Mimulus guttatus*. PLoS Genet. 2014:10(6):e1004410. 10.1371/journal.pgen.1004410. 24967630 PMC4072524

[evae002-B15] Browning SR , Browning BL. Rapid and accurate haplotype phasing and missing-data inference for whole-genome association studies by use of localized haplotype clustering. Am J Hum Genet. 2007:81(5):1084–1097. 10.1086/521987. 17924348 PMC2265661

[evae002-B16] Burri R , Nater A, Kawakami T, Mugal CF, Olason PI, Smeds L, Suh A, Dutoit L, Bureš S, Garamszegi LZ, et al Linked selection and recombination rate variation drive the evolution of the genomic landscape of differentiation across the speciation continuum of *Ficedula* flycatchers. Genome Res. 2015:25(11):1656–1665. 10.1101/gr.196485.115. 26355005 PMC4617962

[evae002-B17] Byrne H , Lynch Alfaro JW, Sampaio I, Farias I, Schneider H, Hrbek T, Boubli JP. Titi monkey biogeography: parallel Pleistocene spread by *Plecturocebus* and *Cheracebus* into a post-Pebas Western Amazon. Zool Scr. 2018:47(5):499–517. 10.1111/zsc.12300.

[evae002-B18] Charif D , Lobry JR. Seqinr 1.0-2: a contributed package to the R project for statistical computing devoted to biological sequences retrieval and analysis. In: Bastolla U Porto M Roman HE, Vendruscolo M, editors. Structural approaches to sequence evolution: molecules, networks, populations. Berlin, Heidelberg: Springer Berlin Heidelberg; 2007. p. 207–232.

[evae002-B19] Charlesworth B . Measures of divergence between populations and the effect of forces that reduce variability. Mol Biol Evol. 1998:15(5):538–543. 10.1093/oxfordjournals.molbev.a025953. 9580982

[evae002-B20] Charlesworth B , Morgan MT, Charlesworth D. The effect of deleterious mutations on neutral molecular variation. Genetics 1993:134(4):1289–1303. 10.1093/genetics/134.4.1289. 8375663 PMC1205596

[evae002-B21] Chase MA , Ellegren H, Mugal CF. Positive selection plays a major role in shaping signatures of differentiation across the genomic landscape of two independent *Ficedula* flycatcher species pairs. Evolution 2021:75(9):2179–2196. 10.1111/evo.14234. 33851440

[evae002-B22] Chikhi L , Sousa VC, Luisi P, Goossens B, Beaumont MA. The confounding effects of population structure, genetic diversity and the sampling scheme on the detection and quantification of population size changes. Genetics 2010:186(3):983–995. 10.1534/genetics.110.118661. 20739713 PMC2975287

[evae002-B23] Coelho LA , Musher LJ, Cracraft J. A multireference-based whole genome assembly for the obligate ant-following antbird, *Rhegmatorhina melanosticta* (Thamnophilidae). Diversity (Basel). 2019:11(9):144. 10.3390/d11090144.

[evae002-B24] Comeron JM . Background selection as baseline for nucleotide variation across the *Drosophila* genome. PLoS Genet. 2014:10(6):e1004434. 10.1371/journal.pgen.1004434. 24968283 PMC4072542

[evae002-B25] Cruickshank TE , Hahn MW. Reanalysis suggests that genomic islands of speciation are due to reduced diversity, not reduced gene flow. Mol Ecol. 2014:23(13):3133–3157. 10.1111/mec.12796. 24845075

[evae002-B26] Csilléry K , François O, Blum MGB. Abc: an R package for approximate Bayesian computation (ABC). Methods Ecol Evol. 2012:3(3):475–479. 10.1111/j.2041-210X.2011.00179.x.

[evae002-B27] Dagosta FCP , De Pinna M. The fishes of the Amazon: distribution and biogeographical patterns, with a comprehensive list of species. Bull Am Mus Nat Hist. 2019:2019(431):1–163. 10.1206/0003-0090.431.1.1.

[evae002-B28] Danecek P , Auton A, Abecasis G, Albers CA, Banks E, DePristo MA, Handsaker RE, Lunter G, Marth GT, Sherry ST, et al The variant call format and VCFtools. Bioinformatics 2011:27(15):2156–2158. 10.1093/bioinformatics/btr330. 21653522 PMC3137218

[evae002-B29] Dapper AL , Payseur BA. Effects of demographic history on the detection of recombination hotspots from linkage disequilibrium. Mol Biol Evol. 2018:35(2):335–353. 10.1093/molbev/msx272. 29045724 PMC5850621

[evae002-B30] Degiorgio M , Huber CD, Hubisz MJ, Hellmann I, Nielsen R. 2016. SweepFinder2: increased sensitivity, robustness and flexibility. Bioinformatics. 32:1895–1897. 27153702 10.1093/bioinformatics/btw051

[evae002-B31] Del-Rio G , Rego MA, Whitney BM, Schunck F, Silveira LF, Faircloth BC, Brumfield RT. Displaced clines in an avian hybrid zone (Thamnophilidae: Rhegmatorhina) within an Amazonian interfluve. Evolution 2021:76(3):455–475. 10.1111/evo.14377. 34626500

[evae002-B32] Delmore KE , Lugo Ramos JS, van Doren BM, Lundberg M, Bensch S, Irwin DE, Liedvogel M. Comparative analysis examining patterns of genomic differentiation across multiple episodes of population divergence in birds. Evol Lett. 2018:2(2):76–87. 10.1002/evl3.46. 30283666 PMC6121856

[evae002-B33] de Oliveira DP , de Carvalho VT, Hrbek T. Cryptic diversity in the lizard genus *Plica* (Squamata): phylogenetic diversity and Amazonian biogeography. Zool Scr. 2016:45(6):630–641. 10.1111/zsc.12172.

[evae002-B34] Dutoit L , Burri R, Nater A, Mugal CF, Ellegren H. Genomic distribution and estimation of nucleotide diversity in natural populations: perspectives from the collared flycatcher (*Ficedula albicollis*) genome. Mol Ecol Resour. 2017:17(4):586–597. 10.1111/1755-0998.12602. 27717155

[evae002-B35] Dutoit L , Vijay N, Mugal CF, Bossu CM, Burri R, Wolf J, Ellegren H. Covariation in levels of nucleotide diversity in homologous regions of the avian genome long after completion of lineage sorting. Proc R Soc B Biol Sci. 2017:284:20162756. 10.1098/rspb.2016.2756. PMC5326536 28202815

[evae002-B36] Edelman NB , Frandsen PB, Miyagi M, Clavijo B, Davey J, Dikow RB, García-Accinelli G, Van Belleghem SM, Patterson N, Neafsey DE, et al Genomic architecture and introgression shape a butterfly radiation. Science 2019:366(6465):594–599. 10.1126/science.aaw2090. 31672890 PMC7197882

[evae002-B37] Ellegren H . Evolutionary stasis: the stable chromosomes of birds. Trends Ecol Evol. 2010:25(5):283–291. 10.1016/j.tree.2009.12.004. 20363047

[evae002-B38] Ellegren H , Smeds L, Burri R, Olason PI, Backström N, Kawakami T, Künstner A, Mäkinen H, Nadachowska-Brzyska K, Qvarnström A, et al The genomic landscape of species divergence in *Ficedula* flycatchers. Nature 2012:491(7426):756–760. 10.1038/nature11584. 23103876

[evae002-B39] Elyashiv E , Sattath S, Hu TT, Strutsovsky A, McVicker G, Andolfatto P, Coop G, Sella G. A genomic map of the effects of linked selection in *Drosophila*. PLoS Genet. 2016:12(8):e1006130. 10.1371/journal.pgen.1006130. 27536991 PMC4990265

[evae002-B40] Ewing GB , Jensen JD. The consequences of not accounting for background selection in demographic inference. Mol Ecol. 2016:25(1):135–141. 10.1111/mec.13390. 26394805

[evae002-B41] Farré M , Micheletti D, Ruiz-Herrera A. Recombination rates and genomic shuffling in human and chimpanzee—a new twist in the chromosomal speciation theory. Mol Biol Evol. 2012:30(4):853–864. 10.1093/molbev/mss272. 23204393 PMC3603309

[evae002-B42] Feng S , Stiller J, Deng Y, Armstrong J, Fang Q, Reeve AH, Xie D, Chen G, Guo C, Faircloth BC, et al Dense sampling of bird diversity increases power of comparative genomics. Nature 2020:587(7833):252–257. 10.1038/s41586-020-2873-9. 33177665 PMC7759463

[evae002-B43] Ferreira M , Fernandes AM, Aleixo A, Antonelli A, Olsson U, Bates JM, Cracraft J, Ribas CC. Evidence for mtDNA capture in the jacamar *Galbula leucogastra*/*chalcothorax* species-complex and insights on the evolution of white-sand ecosystems in the Amazon basin. Mol Phylogenet Evol. 2018:129:149–157. 10.1016/j.ympev.2018.07.007. 30026124

[evae002-B44] Fledel-Alon A , Wilson DJ, Broman K, Wen X, Ober C, Coop G, Przeworski M. Broad-scale recombination patterns underlying proper disjunction in humans. PLoS Genet. 2009:5(9):e1000658. 10.1371/journal.pgen.1000658. 19763175 PMC2734982

[evae002-B45] Fontaine MC , Pease JB, Steele A, Waterhouse RM, Neafsey DE, Sharakhov IV, Jiang X, Hall AB, Catteruccia F, Kakani E, et al Mosquito genomics. Extensive introgression in a malaria vector species complex revealed by phylogenomics. Science 2015:347(6217):1258524. 10.1126/science.1258524. 25431491 PMC4380269

[evae002-B46] Garrigan D , Kingan SB, Geneva AJ, Andolfatto P, Clark AG, Thornton KR, Presgraves DC. Genome sequencing reveals complex speciation in the *Drosophila simulans* clade. Genome Res. 2012:22(8):1499–1511. 10.1101/gr.130922.111. 22534282 PMC3409263

[evae002-B47] Gehara M , Garda AA, Werneck FP, Oliveira EF, da Fonseca EM, Camurugi F, Magalhães FM, Lanna FM, Sites JW, Marques R, et al Estimating synchronous demographic changes across populations using hABC and its application for a herpetological community from northeastern Brazil. Mol Ecol. 2017:26(18):4756–4771. 10.1111/mec.14239. 28734050

[evae002-B48] Gillespie JH . Genetic drift in an infinite population. The pseudohitchhiking model. Genetics 2000:155(2):909–919. 10.1093/genetics/155.2.909. 10835409 PMC1461093

[evae002-B49] Grabherr MG , Russell P, Meyer M, Mauceli E, Alföldi J, Di Palma F, Lindblad-Toh K. Genome-wide synteny through highly sensitive sequence alignment: Satsuma. Bioinformatics 2010:26(9):1145–1151. 10.1093/bioinformatics/btq102. 20208069 PMC2859124

[evae002-B50] Guindon S , Dufayard J-F, Lefort V, Anisimova M, Hordijk W, Gascuel O. New algorithms and methods to estimate maximum-likelihood phylogenies: assessing the performance of PhyML 3.0. Syst Biol. 2010:59(3):307–321. 10.1093/sysbio/syq010. 20525638

[evae002-B51] Haenel Q , Laurentino TG, Roesti M, Berner D. Meta-analysis of chromosome-scale crossover rate variation in eukaryotes and its significance to evolutionary genomics. Mol Ecol. 2018:27(11):2477–2497. 10.1111/mec.14699. 29676042

[evae002-B52] Haffer J . Speciation in Amazonian forest birds. Science 1969:165(3889):131–137. 10.1126/science.165.3889.131. 17834730

[evae002-B53] Haffer J . Hypotheses to explain the origin of species in Amazonia. Braz J Biol. 2008:68(4 Suppl):917–947. 10.1590/S1519-69842008000500003. 19197466

[evae002-B54] Halldorsson BV , Palsson G, Stefansson OA, Jonsson H, Hardarson MT, Eggertsson HP, Gunnarsson B, Oddsson A, Halldorsson GH, Zink F, et al Characterizing mutagenic effects of recombination through a sequence-level genetic map. Science 2019:363(6425):eaau1043. 10.1126/science.aau1043. 30679340

[evae002-B55] Harris RB , Sackman A, Jensen JD. On the unfounded enthusiasm for soft selective sweeps II: examining recent evidence from humans, flies, and viruses. PLoS Genet. 2018:14(12):e1007859. 10.1371/journal.pgen.1007859. 30592709 PMC6336318

[evae002-B56] Harvey MG , Bravo GA, Claramunt S, Cuervo AM, Derryberry GE, Battilana J, Seeholzer GF, McKay JS, O’Meara BC, Faircloth BC, et al The evolution of a tropical biodiversity hotspot. Science 2020:370(6522):1343–1348. 10.1126/science.aaz6970. 33303617

[evae002-B57] Heller R , Chikhi L, Siegismund HR. The confounding effect of population structure on Bayesian skyline plot inferences of demographic history. PLoS One 2013:8(5):e62992. 10.1371/journal.pone.0062992. 23667558 PMC3646956

[evae002-B58] Hudson RR , Kaplan NL. Deleterious background selection with recombination. Genetics 1995:141(4):1605–1617. 10.1093/genetics/141.4.1605. 8601498 PMC1206891

[evae002-B59] Jarvis ED , Mirarab S, Aberer AJ, Li B, Houde P, Li C, Ho SYW, Faircloth BC, Nabholz B, Howard JT, et al Whole-genome analyses resolve early branches in the tree of life of modern birds. Science 2014:346(6215):1320–1331. 10.1126/science.1253451. 25504713 PMC4405904

[evae002-B60] Jensen JD , Payseur BA, Stephan W, Aquadro CF, Lynch M, Charlesworth D, Charlesworth B. The importance of the neutral theory in 1968 and 50 years on: a response to Kern and Hahn 2018. Evolution 2019:73(1):111–114. 10.1111/evo.13650. 30460993 PMC6496948

[evae002-B61] Johri P , Aquadro CF, Beaumont M, Charlesworth B, Excoffier L, Eyre-Walker A, Keightley PD, Lynch M, McVean G, Payseur BA, et al Recommendations for improving statistical inference in population genomics. PLoS Biol. 2022:20(5):e3001669. 10.1371/journal.pbio.3001669. 35639797 PMC9154105

[evae002-B62] Johri P , Charlesworth B, Jensen JD. Toward an evolutionarily appropriate null model: jointly inferring demography and purifying selection. Genetics 2020:215(1):173–192. 10.1534/genetics.119.303002. 32152045 PMC7198275

[evae002-B63] Johri P , Riall K, Becher H, Excoffier L, Charlesworth B, Jensen JD. The impact of purifying and background selection on the inference of population history: problems and prospects. Mol Biol Evol. 2021:38(7):2986–3003. 10.1093/molbev/msab050. 33591322 PMC8233493

[evae002-B64] Jónsson H , Sulem P, Arnadottir GA, Pálsson G, Eggertsson HP, Kristmundsdottir S, Zink F, Kehr B, Hjorleifsson KE, Jensson BÖ, et al Multiple transmissions of de novo mutations in families. Nat Genet. 2018:50(12):1674–1680. 10.1038/s41588-018-0259-9. 30397338

[evae002-B65] Kaback D , Guacci V, Barber D, Mahon J. Chromosome size-dependent control of meiotic recombination. Science 1992:256(5054):228–232. 10.1126/science.1566070. 1566070

[evae002-B66] Kartje ME , Jing P, Payseur BA. Weak correlation between nucleotide variation and recombination rate across the house mouse genome. Genome Biol Evol. 2020:12(4):293–299. 10.1093/gbe/evaa045. 32108880 PMC7186785

[evae002-B67] Kawakami T , Smeds L, Backström N, Husby A, Qvarnström A, Mugal CF, Olason P, Ellegren H. A high-density linkage map enables a second-generation collared flycatcher genome assembly and reveals the patterns of avian recombination rate variation and chromosomal evolution. Mol Ecol. 2014:23(16):4035–4058. 10.1111/mec.12810. 24863701 PMC4149781

[evae002-B68] Kern AD , Hahn MW. The neutral theory in light of natural selection. Mol Biol Evol. 2018:35(6):1366–1371. 10.1093/molbev/msy092. 29722831 PMC5967545

[evae002-B69] Kern AD , Schrider DR. Discoal: flexible coalescent simulations with selection. Bioinformatics 2016:32(24):3839–3841. 10.1093/bioinformatics/btw556. 27559153 PMC5167068

[evae002-B70] Kern AD , Schrider DR. Diplos/HIC: an updated approach to classifying selective sweeps. G3 (Bethesda). 2018:8(6):1959–1970. 10.1534/g3.118.200262. 29626082 PMC5982824

[evae002-B71] Korunes KL , Noor MAF. Gene conversion and linkage: effects on genome evolution and speciation. Mol Ecol. 2017:26(1):351–364. 10.1111/mec.13736. 27337640

[evae002-B72] Krasheninnikova K , Diekhans M, Armstrong J, Dievskii A, Paten B, O’Brien S. halSynteny: a fast, easy-to-use conserved synteny block construction method for multiple whole-genome alignments. Gigascience 2020:9(6):giaa047. 10.1093/gigascience/giaa047. 32463100 PMC7254927

[evae002-B73] Kuhn M . Building predictive models in R using the caret package. J Stat Softw. 2008:28(5):1–26. 10.18637/jss.v028.i05.27774042

[evae002-B74] Langley CH , Stevens K, Cardeno C, Lee YCG, Schrider DR, Pool JE, Langley SA, Suarez C, Corbett-Detig RB, Kolaczkowski B, et al Genomic variation in natural populations of *Drosophila melanogaster*. Genetics 2012:192(2):533–598. 10.1534/genetics.112.142018. 22673804 PMC3454882

[evae002-B75] Li H . A statistical framework for SNP calling, mutation discovery, association mapping and population genetical parameter estimation from sequencing data. Bioinformatics 2011:27(21):2987–2993. 10.1093/bioinformatics/btr509. 21903627 PMC3198575

[evae002-B76] Li H , Durbin R. Fast and accurate short read alignment with Burrows–Wheeler transform. Bioinformatics 2009:25(14):1754–1760. 10.1093/bioinformatics/btp324. 19451168 PMC2705234

[evae002-B77] Li G , Figueiró HV, Eizirik E, Murphy WJ. Recombination-aware phylogenomics reveals the structured genomic landscape of hybridizing cat species. Mol Biol Evol. 2019:36(10):2111–2126. 10.1093/molbev/msz139. 31198971 PMC6759079

[evae002-B78] Li H , Ralph P. Local PCA shows how the effect of population structure differs along the genome. Genetics 2019:211(1):289–304. 10.1534/genetics.118.301747. 30459280 PMC6325702

[evae002-B79] Luna LW , Ribas CC, Aleixo A. Genomic differentiation with gene flow in a widespread Amazonian floodplain-specialist bird species. J Biogeogr. 2021:49:1670–1682. 10.1111/jbi.14257.

[evae002-B80] Lynch Alfaro JW , Boubli JP, Paim FP, Ribas CC, Silva MNF, Messias MR, Röhe F, Mercês MP, Silva Júnior JS, Silva CR, et al Biogeography of squirrel monkeys (genus *Saimiri*): south-central Amazon origin and rapid pan-Amazonian diversification of a lowland primate. Mol Phylogenet Evol. 2015:82 Pt B:436–454. 10.1016/j.ympev.2014.09.004. 25305518

[evae002-B81] Malinsky M , Challis RJ, Tyers AM, Schiffels S, Terai Y, Ngatunga BP, Miska EA, Durbin R, Genner MJ, Turner GF. Genomic islands of speciation separate cichlid ecomorphs in an East African crater lake. Science 2015:350(6267):1493–1498. 10.1126/science.aac9927. 26680190 PMC4700518

[evae002-B82] Mangin B , Siberchicot A, Nicolas S, Doligez A, This P, Cierco-Ayrolles C. Novel measures of linkage disequilibrium that correct the bias due to population structure and relatedness. Heredity (Edinb). 2012:108(3):285–291. 10.1038/hdy.2011.73. 21878986 PMC3282397

[evae002-B83] Manthey JD , Klicka J, Spellman GM. The genomic signature of allopatric speciation in a songbird is shaped by genome architecture (Aves: *Certhia americana*). Genome Biol Evol. 2021:13(8):evab120. 10.1093/gbe/evab120. 34042960 PMC8364988

[evae002-B84] Martin SH , Davey JW, Jiggins CD. Evaluating the use of ABBA–BABA statistics to locate introgressed loci. Mol Biol Evol. 2014:32(1):244–257. 10.1093/molbev/msu269. 25246699 PMC4271521

[evae002-B85] Martin SH , Davey JW, Salazar C, Jiggins CD. Recombination rate variation shapes barriers to introgression across butterfly genomes. PLoS Biol. 2019:17(2):e2006288. 10.1371/journal.pbio.2006288. 30730876 PMC6366726

[evae002-B86] Martin SH , Van Belleghem SM. Exploring evolutionary relationships across the genome using topology weighting. Genetics 2017:206(1):429–438. 10.1534/genetics.116.194720. 28341652 PMC5419486

[evae002-B87] Mazet O , Rodríguez W, Chikhi L. Demographic inference using genetic data from a single individual: separating population size variation from population structure. Theor Popul Biol. 2015:104:46–58. 10.1016/j.tpb.2015.06.003. 26120083

[evae002-B88] McKenna A , Hanna M, Banks E, Sivachenko A, Cibulskis K, Kernytsky A, Garimella K, Altshuler D, Gabriel S, Daly M, et al The Genome Analysis Toolkit: a MapReduce framework for analyzing next-generation DNA sequencing data. Genome Res. 2010:20(9):1297–1303. 10.1101/gr.107524.110. 20644199 PMC2928508

[evae002-B89] McVicker G , Gordon D, Davis C, Green P. Widespread genomic signatures of natural selection in hominid evolution. PLoS Genet. 2009:5(5):e1000471. 10.1371/journal.pgen.1000471. 19424416 PMC2669884

[evae002-B90] Meunier J , Duret L. Recombination drives the evolution of GC-content in the human genome. Mol Biol Evol. 2004:21(6):984–990. 10.1093/molbev/msh070. 14963104

[evae002-B91] Mezmouk S , Dubreuil P, Bosio M, Décousset L, Charcosset A, Praud S, Mangin B. Effect of population structure corrections on the results of association mapping tests in complex maize diversity panels. Theor Appl Genet. 2011:122(6):1149–1160. 10.1007/s00122-010-1519-y. 21221527 PMC3057001

[evae002-B92] Mikkelsen EK , Weir JT. The genome of the Xingu scale-backed antbird (*Willisornis vidua nigrigula*) reveals lineage-specific adaptations. Genomics 2020:112(6):4552–4560. 10.1016/j.ygeno.2020.07.047. 32771623

[evae002-B93] Minh BQ , Schmidt HA, Chernomor O, Schrempf D, Woodhams MD, von Haeseler A, Lanfear R. IQ-TREE 2: new models and efficient methods for phylogenetic inference in the genomic era. Mol Biol Evol. 2020:37(5):1530–1534. 10.1093/molbev/msaa015. 32011700 PMC7182206

[evae002-B94] Mořkovský L , Janoušek V, Reif J, Rídl J, Pačes J, Choleva L, Janko K, Nachman MW, Reifová R. Genomic islands of differentiation in two songbird species reveal candidate genes for hybrid female sterility. Mol Ecol. 2018:27(4):949–958. 10.1111/mec.14479. 29319911 PMC5878113

[evae002-B95] Musher LJ , Giakoumis M, Albert J, Del-Rio G, Rego M, Thom G, Aleixo A, Ribas CC, Brumfield RT, Tilston B, et al 2021. River network rearrangements promote speciation in lowland Amazonian birds. bioRxiv 468717. 10.1101/2021.11.15.468717, 16 November 2021, preprint: not peer reviewed. PMC8993111 35394835

[evae002-B96] Nachman MW , Payseur BA. Recombination rate variation and speciation: theoretical predictions and empirical results from rabbits and mice. Philos Trans R Soc Lond B Biol Sci. 2012:367(1587):409–421. 10.1098/rstb.2011.0249. 22201170 PMC3233716

[evae002-B97] Nadachowska-Brzyska K , Li C, Smeds L, Zhang G, Ellegren H. Temporal dynamics of avian populations during Pleistocene revealed by whole-genome sequences. Curr Biol. 2015:25(10):1375–1380. 10.1016/j.cub.2015.03.047. 25891404 PMC4446789

[evae002-B98] Nam K , Mugal C, Nabholz B, Schielzeth H, Wolf JBW, Backström N, Künstner A, Balakrishnan CN, Heger A, Ponting CP, et al Molecular evolution of genes in avian genomes. Genome Biol. 2010:11(6):R68. 10.1186/gb-2010-11-6-r68. 20573239 PMC2911116

[evae002-B99] Nguyen LT , Schmidt HA, von Haeseler A, Minh BQ. IQ-TREE: a fast and effective stochastic algorithm for estimating maximum-likelihood phylogenies. Mol Biol Evol. 2015:32(1):268–274. 10.1093/molbev/msu300. 25371430 PMC4271533

[evae002-B100] Ortiz EM . vcf2phylip v2.0: convert a VCF matrix into several matrix formats for phylogenetic analysis (v2.0). 2019. Zenodo. 10.5281/zenodo.2540861.

[evae002-B101] Pavlidis P , Laurent S, Stephan W. msABC: a modification of Hudson's ms to facilitate multi-locus ABC analysis. Mol Ecol Resour. 2010:10(4):723–727. 10.1111/j.1755-0998.2010.02832.x. 21565078

[evae002-B102] Peñalba JV , Deng Y, Fang Q, Joseph L, Moritz C, Cockburn A. Genome of an iconic Australian bird: high-quality assembly and linkage map of the superb fairy-wren (*Malurus cyaneus*). Mol Ecol Resour. 2020:20(2):560–578. 10.1111/1755-0998.13124. 31821695

[evae002-B103] Penz C , DeVries P, Tufto J, Lande R. Butterfly dispersal across Amazonia and its implication for biogeography. Ecography 2015:38(4):410–418. 10.1111/ecog.01172.

[evae002-B104] Pessia E , Popa A, Mousset S, Rezvoy C, Duret L, Marais GAB. Evidence for widespread GC-biased gene conversion in eukaryotes. Genome Biol Evol. 2012:4(7):675–682. 10.1093/gbe/evs052. 22628461 PMC5635611

[evae002-B105] Pfeifer B , Wittelsbürger U, Ramos-Onsins SE, Lercher MJ. PopGenome: an efficient Swiss army knife for population genomic analyses in R. Mol Biol Evol. 2014:31(7):1929–1936. 10.1093/molbev/msu136. 24739305 PMC4069620

[evae002-B106] Pouyet F , Aeschbacher S, Thiéry A, Excoffier L. Background selection and biased gene conversion affect more than 95% of the human genome and bias demographic inferences. Elife 2018:7:e36317. 10.7554/eLife.36317. 30125248 PMC6177262

[evae002-B107] Rabiee M , Sayyari E, Mirarab S. Multi-allele species reconstruction using ASTRAL. Mol Phylogenet Evol. 2019:130:286–296. 10.1016/j.ympev.2018.10.033. 30393186

[evae002-B108] R Core Team . R: a language and environment for statistical computing. Vienna, Austria: R Foundation for Statistical Computing; 2021.

[evae002-B109] Ribas CC , Aleixo A, Nogueira ACR, Miyaki CY, Cracraft J. A palaeobiogeographic model for biotic diversification within Amazonia over the past three million years. Proc Biol Sci. 2012:279(1729):681–689. 10.1098/rspb.2011.1120. 21795268 PMC3248724

[evae002-B110] Rousselle M , Mollion M, Nabholz B, Bataillon T, Galtier N. Overestimation of the adaptive substitution rate in fluctuating populations. Biol Lett. 2018:14(5):20180055. 10.1098/rsbl.2018.0055. 29743267 PMC6012701

[evae002-B111] Roux C , Fraïsse C, Castric V, Vekemans X, Pogson GH, Bierne N. Can we continue to neglect genomic variation in introgression rates when inferring the history of speciation? A case study in a *Mytilus* hybrid zone. J Evol Biol. 2014:27(8):1662–1675. 10.1111/jeb.12425. 24913446

[evae002-B112] Schlamp F , Van Der Made J, Stambler R. Evaluating the performance of selection scans to detect selective sweeps in domestic dogs. Mol Ecol. 2016:25(1):342–356. 10.1111/mec.13485. 26589239 PMC4706764

[evae002-B113] Schrider DR . 2020. Background selection does not mimic the patterns of genetic diversity produced by selective sweeps. Genetics. 216:499–519. 32847814 10.1534/genetics.120.303469 PMC7536861

[evae002-B114] Schrider DR , Shanku AG, Kern AD. Effects of linked selective sweeps on demographic inference and model selection. Genetics 2016:204(3):1207–1223. 10.1534/genetics.116.190223. 27605051 PMC5105852

[evae002-B115] Schumer M , Xu C, Powell DL, Durvasula A, Skov L, Holland C, Blazier JC, Sankararaman S, Andolfatto P, Rosenthal GG, et al Natural selection interacts with recombination to shape the evolution of hybrid genomes. Science 2018:360(6389):656–660. 10.1126/science.aar3684. 29674434 PMC6069607

[evae002-B116] Schumer M , Xu C, Powell DL, Durvasula A, Skov L, Holland C, Sankararaman S, Andolfatto P, Rosenthal GG, Przeworski M, et al 2017. Natural selection interacts with the local recombination rate to shape the evolution of hybrid genomes. bioRxiv 212407. 10.1101/212407, 1 November 2017, preprint: not peer reviewed. PMC6069607 29674434

[evae002-B117] Seehausen O , Butlin RK, Keller I, Wagner CE, Boughman JW, Hohenlohe PA, Peichel CL, Saetre G-P, Bank C, Brännström Å, et al Genomics and the origin of species. Nat Rev Genet. 2014:15(3):176–192. 10.1038/nrg3644. 24535286

[evae002-B118] Silva SM , Peterson AT, Carneiro L, Burlamaqui TCT, Ribas CC, Sousa-Neves T, Miranda LS, Fernandes AM, d'Horta FM, Araújo-Silva LE, et al A dynamic continental moisture gradient drove Amazonian bird diversification. Sci Adv. 2019:5(7):eaat5752. 10.1126/sciadv.aat5752. 31281878 PMC6609164

[evae002-B119] Singhal S , et al 2015. Stable recombination hotspots in birds. Science. 350:928–932. 26586757 10.1126/science.aad0843 PMC4864528

[evae002-B120] Smith TCA , Arndt PF, Eyre-Walker A. Large scale variation in the rate of germ-line de novo mutation, base composition, divergence and diversity in humans. PLoS Genet. 2018:14(3):e1007254. 10.1371/journal.pgen.1007254. 29590096 PMC5891062

[evae002-B121] Smith JM , Haigh J. The hitch-hiking effect of a favourable gene. Genet Res. 1974:23(1):23–35. 10.1017/S0016672300014634. 4407212

[evae002-B122] Smith BT , McCormack JE, Cuervo AM, Hickerson MJ, Aleixo A, Cadena CD, Pérez-Emán J, Burney CW, Xie X, Harvey MG, et al The drivers of tropical speciation. Nature 2014:515(7527):406–409. 10.1038/nature13687. 25209666

[evae002-B123] Solís-Lemus C , Ané C. Inferring phylogenetic networks with maximum pseudolikelihood under incomplete lineage sorting. PLoS Genet. 2016:12(3):e1005896. 10.1371/journal.pgen.1005896. 26950302 PMC4780787

[evae002-B124] Stankowski S , Chase MA, Fuiten AM, Rodrigues MF, Ralph PL, Streisfeld MA. Widespread selection and gene flow shape the genomic landscape during a radiation of monkeyflowers. PLoS Biol. 2019:17(7):e3000391. 10.1371/journal.pbio.3000391. 31339877 PMC6660095

[evae002-B125] Stephan W . 2010. Genetic hitchhiking versus background selection: the controversy and its implications. Philos Trans R Soc Lond B Biol Sci. 365:1245–1253. 20308100 10.1098/rstb.2009.0278 PMC2871815

[evae002-B126] Terhorst J , Kamm JA, Song YS. Robust and scalable inference of population history from hundreds of unphased whole genomes. Nat Genet. 2017:49(2):303–309. 10.1038/ng.3748. 28024154 PMC5470542

[evae002-B127] Thom G , Aleixo A. Cryptic speciation in the white-shouldered antshrike (*Thamnophilus aethiops*, Aves—Thamnophilidae): the tale of a transcontinental radiation across rivers in lowland Amazonia and the northeastern Atlantic Forest. Mol Phylogenet Evol. 2015:82:95–110. 10.1016/j.ympev.2014.09.023. 25291073

[evae002-B128] Thom G , Xue AT, Sawakuchi AO, Ribas CC, Hickerson MJ, Aleixo A, Miyaki C. Quaternary climate changes as speciation drivers in the Amazon floodplains. Sci Adv. 2020:6(11):eaax4718. 10.1126/sciadv.aax4718. 32195336 PMC7065905

[evae002-B129] Tigano A , Khan R, Omer AD, Weisz D, Dudchenko O, Multani AS, Pathak S, Behringer RR, Aiden EL, Fisher H, et al Chromosome size affects sequence divergence between species through the interplay of recombination and selection. Evolution 2022:76(4):782–798. 10.1111/evo.14467. 35271737 PMC9314927

[evae002-B130] Van Belleghem SM , Baquero M, Papa R, Salazar C, McMillan WO, Counterman BA, Jiggins CD, Martin SH. Patterns of Z chromosome divergence among *Heliconius* species highlight the importance of historical demography. Mol Ecol. 2018:27(19):3852–3872. 10.1111/mec.14560. 29569384 PMC6151167

[evae002-B131] Van Doren BM , Campagna L, Helm B, Illera JC, Lovette IJ, Liedvogel M. Correlated patterns of genetic diversity and differentiation across an avian family. Mol Ecol. 2017:26:3982–3997. 10.1111/mec.14083. 28256062

[evae002-B132] Vijay N , Weissensteiner M, Burri R, Kawakami T, Ellegren H, Wolf JBW. Genomewide patterns of variation in genetic diversity are shared among populations, species and higher-order taxa. Mol Ecol. 2017:26(16):4284–4295. 10.1111/mec.14195. 28570015

[evae002-B133] Waterhouse RM , Seppey M, Simão FA, Manni M, Ioannidis P, Klioutchnikov G, Kriventseva EV, Zdobnov EM. BUSCO applications from quality assessments to gene prediction and phylogenomics. Mol Biol Evol. 2018:35(3):543–548. 10.1093/molbev/msx319. 29220515 PMC5850278

[evae002-B134] Watterson GA . On the number of segregating sites in genetical models without recombination. Theor Popul Biol. 1975:7(2):256–276. 10.1016/0040-5809(75)90020-9. 1145509

[evae002-B135] Wayne ML , Simonsen KL. Statistical tests of neutrality in the age of weak selection. Trends Ecol Evol. 1998:13(6):236–240. 10.1016/S0169-5347(98)01360-3. 21238278

[evae002-B136] Weir JT , Faccio MS, Pulido-Santacruz P, Barrera-Guzmán AO, Aleixo A. Hybridization in headwater regions, and the role of rivers as drivers of speciation in Amazonian birds. Evolution 2015:69(7):1823–1834. 10.1111/evo.12696. 26095719

[evae002-B137] Wen D , Yu Y, Zhu J, Nakhleh L. Inferring phylogenetic networks using PhyloNet. Syst Biol. 2018:67(4):735–740. 10.1093/sysbio/syy015. 29514307 PMC6005058

[evae002-B138] Willis EO . 1967. The behavior of bicolored antbirds. Univ Calif Publ Zool. 79:1–132. 10.2307/4511425

[evae002-B139] Willis EO . Diversity in adversity: the behaviors of two subordinate antbirds. Arquivos de Zoologia. 1981:30(3):159–234. 10.11606/issn.2176-7793.v30i3p159-234.

[evae002-B140] Willis EO , Oniki Y. Birds and army ants. Ann Rev Ecal Syst. 1978:9(1):243–263. 10.1146/annurev.es.09.110178.001331.

[evae002-B141] Wolf JBW , Ellegren H. Making sense of genomic islands of differentiation in light of speciation. Nat Rev Genet. 2017:18(2):87–100. 10.1038/nrg.2016.133. 27840429

[evae002-B142] Wu TD , Watanabe CK. GMAP: a genomic mapping and alignment program for mRNA and EST sequences. Bioinformatics 2005:21(9):1859–1875. 10.1093/bioinformatics/bti310. 15728110

[evae002-B143] Zeng K . A coalescent model of background selection with recombination, demography and variation in selection coefficients. Heredity (Edinb). 2013:110(4):363–371. 10.1038/hdy.2012.102. 23188176 PMC3607694

[evae002-B144] Zhang G , Li C, Li Q, Li B, Larkin DM, Lee C, Storz JF, Antunes A, Greenwold MJ, Meredith RW, et al Comparative genomics reveals insights into avian genome evolution and adaptation. Science 2014:346(6215):1311–1320. 10.1126/science.1251385. 25504712 PMC4390078

[evae002-B145] Zhang C , Rabiee M, Sayyari E, Mirarab S. ASTRAL-III: polynomial time species tree reconstruction from partially resolved gene trees. BMC Bioinformatics 2018:19(S6):153. 10.1186/s12859-018-2129-y. 29745866 PMC5998893

[evae002-B146] Zheng X , Levine D, Shen J, Gogarten SM, Laurie C, Weir BS. A high-performance computing toolset for relatedness and principal component analysis of SNP data. Bioinformatics 2012:28(24):3326–3328. 10.1093/bioinformatics/bts606. 23060615 PMC3519454

