# Supplementary material for: Genomic Architecture Predicts Tree Topology, Population Structuring, and Demographic History in Amazonian Birds
Source: Genome Biol Evol. 2024 Jan 17;16(1):evae002. doi: 10.1093/gbe/evae002 (PMC10823491; doi:10.1093/gbe/evae002)
Supplement: evae002_Supplementary_Data [file evae002_supplementary_data.zip › Thom et al. 2023 GBE supplementary material.docx]

**Supplementary information**

**Supplementary results**

*Historical DNA and reference mapping*

Samples obtained from toe pads had similar coverage and amount of missing data (mean coverage = 9.54x; mean missingness = 0.18), compared to tissue samples (mean coverage = 10.69x; mean missingness = 0.17), despite the greater number of sequenced reads (mean number of reads for tissue samples = 117 million; mean number of reads for toe pad samples = 268 million). This was associated with the shorter insert size of toe pad samples, leading to an average of 39% of the reads being excluded due to paired-end overlap, compared to 12% in tissue samples.

On average, a high proportion of reads correctly mapped to pseudo-chromosome reference genomes in *P. nigromaculata* (93.1%), *X. spixii* (96.3%), and *L. vociferans* (87.6%). The proportion of mapped reads was associated with the degree of divergence between the reference genome and the targeted species, with X. spixii having the highest proportion of mapped reads and L. vociferans the lowest. The number of segregating sites was of a similar magnitude but varied between species: *P. nigromaculata* (n = 20,838,931), *X. spixii* (n = 26,583,784), and *L. vociferans* (n = 21,769,167). The proportion of missing sites per individual was, on average, 18% (Table S1).

To test if the pseudochromosome approach used to assign scaffolds of our reference genomes to chromosomes of distantly related species (e.g., Zebra Finch) affected the correlations between chromosome size and phylogenetic and demographic parameters, we reran our analyses using only syntenic genomic regions within homologous chromosomes between R. melanosticta (pseudochromosomes; see Coelho et al. 2019) and *Chiroxiphia* *lanceolata*. Our results indicate that 95.7% of the analyzed genomic windows were conserved in regard to chromosomic assignment between species, and that the correlation index and p-values for all explored parameters remained relatively unchanged after removing regions located in distinct chromosomes between species (Figure S18; Table S21). In a scenario with high levels of chromosomal rearrangements within passerine birds, our approach will fail to accurately recover the genomic location of the reference genomes’ scaffolds by assuming the chromosomal structure of the Zebra Finch and other more distantly related species. However, contrary to what we found, this bias would lead to a lack of association between chromosome size and phylogenetic metrics, given that the reshuffle of genomic regions between small and large chromosomes would homogenize the average estimates between chromosomes. By aligning one of our reference genomes to the genome of *Chiroxiphia lanceolata*, a more closely related taxon than Zebra Finch, we validated the high synteny between the genomes of passerine birds, as reported elsewhere (Coelho et al. 2019; Peñalba et al. 2020; Ellegren 2010). This level of synteny indicates chromosome rearrangements and structural variants between analyzed species do not affect genome-wide correlations between chromosome size and phylogenetic or demographic estimates.

*Genome-wide genetic structure*

Genome-wide levels of differentiation between species match the evolutionary expectations associated with their life histories. The least dispersive species that inhabit the understory, *P. nigromaculata*, had the most pronounced levels of genetic structure across rivers, followed by *X. spixii* which occupies the midstory, and the most dispersive, canopy species, *L. vociferans*, had the shallowest structure. To visualize patterns of genetic structure based on independently evolving sets of SNPs (linkage disequilibrium R2 < 0.2), we used Principal Components Analyses (PCAs). Three isolated clusters of individuals supported strong geographic structure within *P. nigromaculata*, consistent with previous studies based on mtDNA, spatially matching areas of endemism (Silva et al. 2019; Alexandre Aleixo et al. 2009; Figure S19). In *X. spixii*, the PCA supported strong differentiation between the Tapajos from Belem and Xingu populations, which had a substantial overlap in PC1 (8.8% of the explained variance; Figure S19). For *L. vociferans*, all samples clustered together, indicating a lack of spatial structure in the genetic variation (Figure S19). In agreement with these results, the average FST between populations was considerably higher in *P. nigromaculata* (mean FST = 0.1262; SD = 0.09) than in *X. spixii* (mean FST = 0.059; SD = 0.046) and *L. vociferans* (mean FST = 0.008; SD = 0.019)

*Phylogenetic signal was associated with genomic architecture.*

The weight for alternative topologies varied considerably across windows and was associated with genomic architecture. To test how the probability of alternative topologies varied across the genome of each species, we calculated topology weights using Twisst (Martin & Van Belleghem 2017). When averaging weights for genome-wide windows of P. nigromaculata we observed a higher weight for Topology 2, followed closely by Topology 1. Genomic windows from P. nigromaculata based on upper and lower thresholds for summary statistics showed substantial variation in which topology had the highest average weight, consistent with our species tree approach (Figure 5). For the other two species, we found less variation along the genome for the topology with the highest average weight. The topology with the highest weight also varied across chromosomes of different sizes. Smaller chromosomes in P. nigromaculata had a higher weight for Topology 2, putatively derived from gene flow (Figure 6). In X. spixii we observed a progressive increase in the weight for the species tree (Topology 2) in larger chromosomes, despite a non-significant correlation, whereas in L. vociferans all three topologies had a similar weight across chromosomes.

*Exploring the effects of gene flow and recombination rate on gene trees with coalescent simulations*

The conflicting phylogenetic pattern observed for *P. nigromaculata* could be driven by gene flow increasing the signal for the topology where introgressing populations are sisters. To further explore how topology weight varied according to gene flow and intralocus recombination, we performed coalescent simulations with demographic parameters similar to those estimated for *P. nigromaculata*, and we calculated topology weights using the approach mentioned above. Our simulations suggested that in the absence of gene flow, the frequency of the alternative topologies (Topologies 2 and 3) to the species tree (topology 1) was similar (Figure S20). The presence of gene flow between non-sister species produces a deviation from this pattern by increasing the average weight for the topology with introgressing populations as sisters (Topology 2), as we found in our observed data for *P. nigromaculata*. This relationship was further intensified by intra-locus recombination (Figure S20). Although our simulations corroborate that recombination rate by itself does not affect levels of genetic diversity (Hudson 1983), it does affect levels of incomplete lineage sorting (ILS) between populations when gene flow is present by increasing the variance of topology weights across the genome (Figure S20). It is important to mention that linked selection and recombination rate should not affect rates of substitution between populations (Birky and Walsh 1988) but how the higher impact of gene flow in areas with high recombination might affect this pattern is still not fully understood. When comparing the results obtained with this simulation approach with the genome-wide topology weights obtained for *P. nigromaculata*, our results suggest that gene flow between non-sister taxa was likely increasing the weights for one of the two best topologies.

**Supplementary Tables**

Table S1:Specimens sequenced in this study for three species of Amazonian Birds. Available at (DOI will be provided upon acceptance)

Tables S2: Benchmarking Universal Single-Copy Orthologs analyses (BUSCO) output.

|  | R. melanosticta | X. elegans | C. ornatus |
| --- | --- | --- | --- |
| Complete BUSCOs (C) | 4389 | 4378 | 4588 |
| Complete and single-copy BUSCOs (S) | 4341 | 4320 | 4536 |
| Complete and duplicated BUSCOs (D) | 48 | 58 | 52 |
| Fragmented BUSCOs (F) | 163 | 272 | 209 |
| Missing BUSCOs (M) | 363 | 265 | 118 |
| Total BUSCO groups searched | 4915 | 4915 | 4915 |
| Total % | 89.3 | 89.1 | 93.4 |

Table S3: Summary statistics calculated for 100kb non overlapping widows for *Phlegopsis nigromaculata*. Available at  [(DOI will be provided upon acceptance)](https://github.com/GregoryThom/Genomic-architecture-Amazonian-birds)

Table S4: Summary statistics calculated for 100kb non overlapping widows for *Xiphorhynchus spixii*. Available at  [(DOI will be provided upon acceptance)](https://github.com/GregoryThom/Genomic-architecture-Amazonian-birds)

Table S5: Summary statistics calculated for 100kb non overlapping widows for *Lipaugus vociferans*. Available at  [(DOI will be provided upon acceptance)](https://github.com/GregoryThom/Genomic-architecture-Amazonian-birds)

Table S6: P-values for the correlogram (Pearson’s correlation index) between population genetics summary statistics, phylogenetic weights, and genomic architecture estimates for *Phlegopsis nigromaculata*. Correlogram is available on Figure S1. Available at  [(DOI will be provided upon acceptance)](https://github.com/GregoryThom/Genomic-architecture-Amazonian-birds)

Table S7: P-values for the correlogram (Pearson’s correlation index) between population genetics summary statistics, phylogenetic weights, and genomic architecture estimates for *Xiphorhynchus spixii*. Correlogram is available on Figure S2. Available at  [(DOI will be provided upon acceptance)](https://github.com/GregoryThom/Genomic-architecture-Amazonian-birds)

Table S8: P-values for the correlogram (Pearson’s correlation index) between population genetics summary statistics, phylogenetic weights, and genomic architecture estimates for *Lipaugus vociferans* Correlogram is available on Figure S3. Available at  [(DOI will be provided upon acceptance)](https://github.com/GregoryThom/Genomic-architecture-Amazonian-birds)

Table S9: P-values for the correlogram (Pearson’s correlation index) between population genetics summary statistics, phylogenetic weights, and genomic architecture estimates for *Phlegopsis nigromaculata*, based on the average values per chromosome. Correlogram is available on Figure S4. Available at  [(DOI will be provided upon acceptance)](https://github.com/GregoryThom/Genomic-architecture-Amazonian-birds)

Table S10: P-values for the correlogram (Pearson’s correlation index) between population genetics summary statistics, phylogenetic weights, and genomic architecture estimates for *Xiphorhynchus spixii*, based on the average values per chromosome. Correlogram is available on Figure S5. Available at  [(DOI will be provided upon acceptance)](https://github.com/GregoryThom/Genomic-architecture-Amazonian-birds)

Table S11: P-values for the correlogram (Pearson’s correlation index) between population genetics summary statistics, phylogenetic weights, and genomic architecture estimates for *Lipaugus vociferans*, based on the average values per chromosome. Correlogram is available on Figure S6. Available at  [(DOI will be provided upon acceptance)](https://github.com/GregoryThom/Genomic-architecture-Amazonian-birds)

Table S12: LOESS models results between population genetics summary statistics and genomic architecture variables.

| Species | Model | span | RMSE | R^2^ | MAE |
| --- | --- | --- | --- | --- | --- |
| *P. nigromaculata* | Topology 1 weight ~ Recombination | 0.5 | 986.341 | 0.040 | 741.486 |
| *P. nigromaculata* | Topology 2 weight ~ Recombination | 0.5 | 1019.543 | 0.064 | 770.654 |
| *P. nigromaculata* | Topology 3 weight ~ Recombination | 0.5 | 895.657 | 0.010 | 671.060 |
| *P. nigromaculata* | D statistics ~ Recombination | 0.5 | 0.105 | 0.030 | 0.079 |
| *P. nigromaculata* | Theta Pi Tapajos ~ Recombination | 0.5 | 0.001 | 0.325 | 0.001 |
| *P. nigromaculata* | Theta Pi Tapajos ~ Recombination + Gene density | 0.5 | 0.001 | 0.329 | 0.001 |
| *P. nigromaculata* | Gene density ~ Recombination | 0.5 | 0.030 | 0.023 | 0.022 |
| *P. nigromaculata* | Theta Pi Tapajos ~ GC content | 0.5 | 0.001 | 0.110 | 0.001 |
| *X. spixii* | Topology 1 weight ~ Recombination | 0.5 | 641.862 | 0.113 | 468.449 |
| *X. spixii* | Topology 2 weight ~ Recombination | 0.5 | 1145.018 | 0.147 | 823.209 |
| *X. spixii* | Topology 3 weight ~ Recombination | 0.5 | 630.543 | 0.134 | 455.687 |
| *X. spixii* | D statistics ~ Recombination | 0.5 | 0.059 | 0.004 | 0.044 |
| *X. spixii* | Theta Pi Tapajos ~ Recombination | 0.5 | 0.002 | 0.431 | 0.001 |
| *X. spixii* | Theta Pi Tapajos ~ Recombination + Gene density | 0.5 | 0.001 | 0.650 | 0.001 |
| *X. spixii* | Gene density ~ Recombination | 0.5 | 0.023 | 0.030 | 0.017 |
| *X. spixii* | Theta Pi Tapajos ~ GC content | 0.5 | 0.002 | 0.260 | 0.001 |
| *L. vociferans* | Topology 1 weight ~ Recombination | 0.5 | 113.203 | 0.004 | 87.709 |
| *L. vociferans* | Topology 2 weight ~ Recombination | 0.5 | 134.331 | 0.027 | 105.043 |
| *L. vociferans* | Topology 3 weight ~ Recombination | 0.5 | 136.926 | 0.029 | 105.716 |
| *L. vociferans* | D statistics ~ Recombination | 0.5 | 0.089 | 0.024 | 0.066 |
| *L. vociferans* | Theta Pi Tapajos ~ Recombination | 0.5 | 0.001 | 0.353 | 0.001 |
| *L. vociferans* | Theta Pi Tapajos ~ Recombination + Gene density | 0.5 | 0.001 | 0.406 | 0.001 |
| *L. vociferans* | Gene density ~ Recombination | 0.5 | 0.022 | 0.027 | 0.013 |
| *L. vociferans* | Theta Pi Tapajos ~ GC content | 0.5 | 0.001 | 0.031 | 0.001 |

Table S13: LOESS models results between genetic diversity (π) and recombination rate for randomly sampled windows across the genome and neutral areas inferred by diploS/HIC.

| Model | RMSE | R^2^ | MAE | # windows | Species |
| --- | --- | --- | --- | --- | --- |
| Neutral | 0.0003 | 0.30 | 0.0002 | 1476 | *P. nigromaculata* |
| Whole genome | 0.0003 | 0.36 | 0.0002 | 1476 | *P. nigromaculata* |
| Neutral | 0.0002 | 0.48 | 0.002 | 1235 | *X. spixii* |
| Whole genome | 0.0002 | 0.65 | 0.002 | 1235 | *X. spixii* |
| Neutral | 0.0003 | 0.33 | 0.002 | 980 | *L. vociferans* |
| Whole genome | 0.0003 | 0.39 | 0.002 | 980 | *L. vociferans* |

Table S14: Neural network testing step performance for the multi-class model classification implemented in DiploS/HIC.

| Parameter | *P. nigromaculata* | *L. vociferans* | *X. spixii* |
| --- | --- | --- | --- |
| Loss | 0.924 | 0.856 | 0.781 |
| Accuracy | 0.722 | 0.686 | 0.688 |
| FPR | 0.258 | 0.276 | 0.278 |
| Recall | 0.698 | 0.662 | 0.666 |
| AUC | 0.939 | 0.92 | 0.927 |

FPR - False Positive Rate; AUC - Area Under the Curve

Table S15: Demographic parameter estimation for *P. nigromaculata* obtained with a Neural Network regression model based on 5,000 genomic windows of 10kb.

| Parameter | Average across 10 replicates | SD | R^2^ | MAE |
| --- | --- | --- | --- | --- |
| Ne Tapajos | 259924.184 | 12977.39 | 0.993 | 12801.096 |
| Ne Xingu | 169100.342 | 6053.634 | 0.999 | 6531.002 |
| Ne Belem | 332015.849 | 21924.035 | 0.994 | 12469.778 |
| Divergence time Tapajos/Xingu | 77866.978 | 17849.052 | 0.969 | 37810.559 |
| Divergence time Belem(Tapajos/Xingu) | 149836.929 | 15272.708 | 0.974 | 36576.586 |
| Migration Tapajos -> Xingu | 0.002 | 0.005 | 0.917 | 0.161 |
| Migration Tapajos <- Xingu | 0.881 | 0.659 | 0.776 | 0.301 |
| Migration Xingu -> Belem | 0.484 | 0.486 | 0.962 | 0.138 |
| Migration Xingu -> Belem | 0.035 | 0.072 | 0.861 | 0.198 |

Ne - Effective population size

Table S16: Demographic parameter estimation for *X. spixii* obtained with a Neural Network regression model based on 5,000 genomic windows of 10kb.

| Parameters | Average across 10 replicates | SD | R^2^ | MAE |
| --- | --- | --- | --- | --- |
| Ne Tapajos | 517153.561 | 34287.162 | 0.995 | 11445.349 |
| Ne Xingu | 488411.928 | 64266.264 | 0.995 | 11262.503 |
| Ne Belem | 168861.451 | 28343.541 | 0.998 | 6569.784 |
| Divergence time Tapajos/Xingu | 40303.463 | 16236.143 | 0.973 | 32798.519 |
| Divergence time Belem(Tapajos/Xingu) | 218858.047 | 12095.599 | 0.986 | 30668.129 |
| Migration Tapajos -> Xingu | 0.965 | 0.662 | 0.879 | 0.182 |
| Migration Tapajos <- Xingu | 0.745 | 0.145 | 0.972 | 0.106 |
| Migration Xingu -> Belem | 1.25 | 0.383 | 0.895 | 0.186 |
| Migration Xingu -> Belem | 2.075 | 0.144 | 0.939 | 0.139 |

Ne - Effective population size

Table S17: Demographic parameter estimation for *L. vociferans* obtained with a Neural Network regression model based on 5,000 genomic windows of 10kb.

| Parameters | Average across 10 replicates | R^2^ | MAE |
| --- | --- | --- | --- |
| Ne Tapajos | 395748.563 | 0.983 | 13956.26 |
| Ne Xingu | 409559.188 | 0.98 | 7546.477 |
| Ne Belem | 294531.406 | 0.97 | 12449.989 |
| Divergence time Tapajos/Xingu | 26090.762 | 0.87 | 3382.221 |
| Divergence time Belem(Tapajos/Xingu) | 36558.706 | 0.874 | 2728.545 |
| Migration Tapajos -> Xingu | 2.347 | 0.852 | 0.175 |
| Migration Tapajos <- Xingu | 1.956 | 0.792 | 0.225 |
| Migration Xingu -> Belem | 1.827 | 0.863 | 0.205 |
| Migration Xingu -> Belem | 2.125 | 0.923 | 0.179 |

Ne - Effective population size

Table S18: Model probability for the three alternative unrooted topologies between Tapajos, Xingu, and Belem areas of endemism (plus outgroup), obtained with a Neural Network multi-class model classification approach based on 5,000 genomic windows of 10kb.

| Species | Topology 1 | Topology 2 | Topology 3 |
| --- | --- | --- | --- |
| *Phlegopsis nigromaculata* | 0.865 | 0.124 | 0.011 |
| *Xiphorhynchus spixii* | 0.000 | 1.000 | 0.000 |
| *Lipaugus vociferans* | 0.542 | 0.285 | 0.172 |

Table S19: LOESS models for demographic parameters estimated with a Neural Network regression model based on genome-wide windows of 100 kbp as response variables, and recombination rate as predictor variable for the three studied species.

| Species | span | RMSE | R^2^ | MAE | Response variable |
| --- | --- | --- | --- | --- | --- |
| *P. nigromaculata* | 0.5 | 0.4527 | 0.0055 | 0.4336 | Probability for Topology 1 |
| *P. nigromaculata* | 0.5 | 0.4527 | 0.0049 | 0.4335 | Probability for Topology 2 |
| *P. nigromaculata* | 0.5 | 88313.7500 | 0.2725 | 69768.1481 | Ne Tapajos |
| *P. nigromaculata* | 0.5 | 0.1086 | 0.1405 | 0.0873 | Gene flow |
| *P. nigromaculata* | 0.5 | 28463.0250 | 0.0172 | 21342.1566 | TMRCA |
| *X. spixii* | 0.5 | 0.0537 | 0.0028 | 0.0087 | Probability for Topology 1 |
| *X. spixii* | 0.5 | 0.0526 | 0.0037 | 0.0087 | Probability for Topology 2 |
| *X. spixii* | 0.5 | 93266.0627 | 0.5648 | 72882.1395 | Ne Tapajos |
| *X. spixii* | 0.5 | 0.0823 | 0.2621 | 0.0661 | Gene flow |
| *X. spixii* | 0.5 | 46318.1524 | 0.0086 | 34985.3272 | TMRCA |
| *L. vociferans* | 0.5 | 0.1109 | 0.1520 | 0.0390 | Probability for Topology 1 |
| *L. vociferans* | 0.5 | 0.1105 | 0.1502 | 0.0390 | Probability for Topology 2 |
| *L. vociferans* | 0.5 | 73405.4776 | 0.3645 | 57788.2004 | Ne Tapajos |
| *L. vociferans* | 0.5 | 0.0246 | 0.0242 | 0.0186 | Gene flow |
| *L. vociferans* | 0.5 | 8567.5522 | 0.1832 | 6628.1080 | TMRCA |

Effective population size (*Ne*); Time to the most recent common ancestor of the most recent divergence event (TMRCA); and Gene flow rate between Tapajos and Xingu populations (Gene flow).

Table S20: Set of uniform priors for demographic parameters implemented in our Neural network approaches for model selection.

| Parameter | Lower bound | Upper bound |
| --- | --- | --- |
| Ne Tapajos | 50,000 | 700,000 |
| Ne Xingu | 50,000 | 700,000 |
| Ne Belem | 50,000 | 700,000 |
| Migration Tapajos -> Xingu | 0.01 | 2.00 |
| Migration Tapajos -> Belem | 0.00 | 0.00 |
| Migration Xingu -> Tapajos | 0.01 | 2.00 |
| Migration Xingu -> Belem | 0.01 | 2.00 |
| Migration Belem -> Tapajos | 0.00 | 0.00 |
| Migration Belem -> Xingu | 0.01 | 2.00 |
| Divergence Time (generations) Tapajos/Xingu | 10,000 | 1,000,000 |
| Divergence Time (generations) Belem(Tapajos/Xingu) | 10,000 | 1,000,000 |
| Mutation rate (u) | 2.42E-09 | 2.42E-09 |

Effective population size (*Ne*).

Table S21: Demographic parameters used in our simulation approach to explore the effects of recombination and gene flow on topology weights. We tested four alternative models varying the presence of intra-locus recombination and gene flow among non sister taxa.

| Parameters | No recombination/No gene flow | Recombination/No gene flow | No recombination/Gene flow | Recombination/Gene flow |
| --- | --- | --- | --- | --- |
| Ne pop1 | 20000 | 20000 | 20000 | 20000 |
| Ne pop2 | 20000 | 20000 | 20000 | 20000 |
| Ne pop3 | 20000 | 20000 | 20000 | 20000 |
| Ne outgroup | 20000 | 20000 | 20000 | 20000 |
| Tdiv 1-2 | 100000 | 100000 | 100000 | 100000 |
| Tdiv 3(1-2) | 300000 | 300000 | 300000 | 300000 |
| Tdiv 4(3(1-2)) | 700000 | 700000 | 700000 | 700000 |
| mig 2->3 | 0 | 0 | 1-10 | 1-10 |
| mig 3->2 | 0 | 0 | 1-10 | 1-10 |
| Recombination | 0 | 1E-08 | 0 | 1E-08 |
| Mutation Rate | 2.42E-09 | 2.42E-09 | 2.42E-09 | 2.42E-09 |

Effective population size (Ne); Divergence time (Tdiv); and Gene flow (mig).

Table S21:P-values for the correlogram (Pearson’s correlation index) between population genetics summary statistics, phylogenetic weights, and genomic architecture estimates for *Phlegopsis nigromaculata*, based on the average values per chromosome for syntenic regions within homologous chromosomes between the *Rhegmatorhina melanosticta* (pseudochromosomes) and *Chiroxiophia lanceolata* genomes. A correlogram is available in Figure S18. Available at  [(DOI will be provided upon acceptance)](https://github.com/GregoryThom/Genomic-architecture-Amazonian-birds)

**Supplementary Figures**


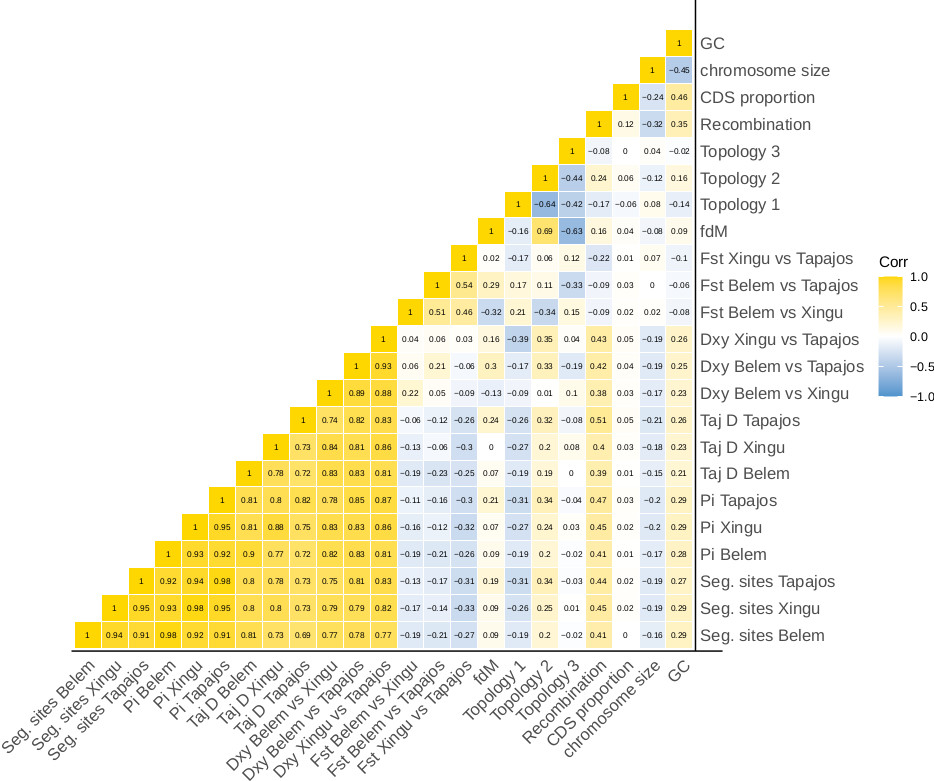


Figure S1: Correlogram (Pearson’s correlation index) between population genetics summary statistics, phylogenetic weights, and genomic architecture estimates for *Phlegopsis nigromaculata*. P-values are available on supplementary table 6.


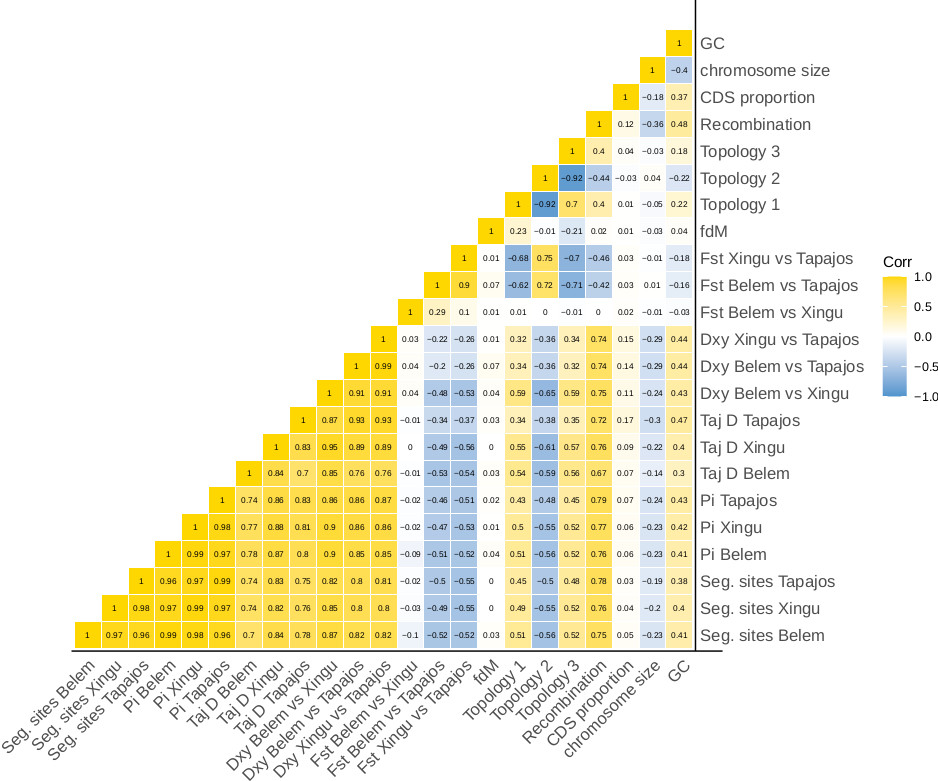


Figure S2: Correlogram (Pearson’s correlation index) between population genetics summary statistics, phylogenetic weights, and genomic architecture estimates for *Xiphorhynchus spixii*. P-values are available on supplementary table 7.


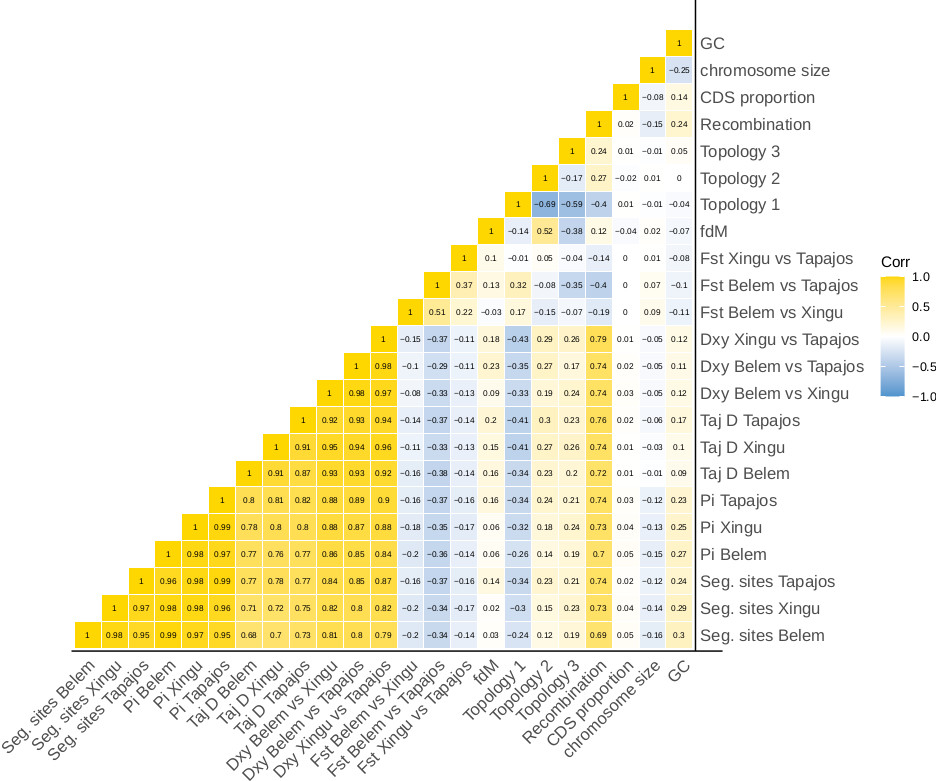


Figure S3: Correlogram (Pearson’s correlation index) between population genetics summary statistics, phylogenetic weights, and genomic architecture estimates for *Lipaugus vociferans.* P-values are available on supplementary table 8.


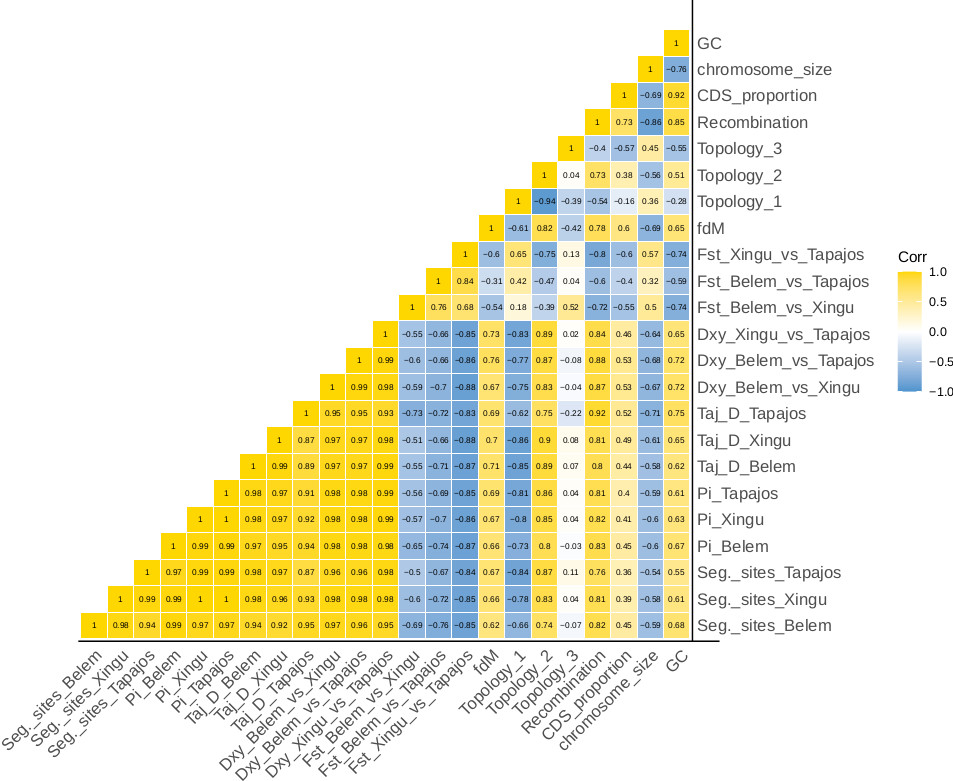


Figure S4: Correlogram (Pearson’s correlation index) between population genetics summary statistics, phylogenetic weights, and genomic architecture estimates for *P. nigromaculata*, based on the average values per chromosome. P-values are available on supplementary table 9.
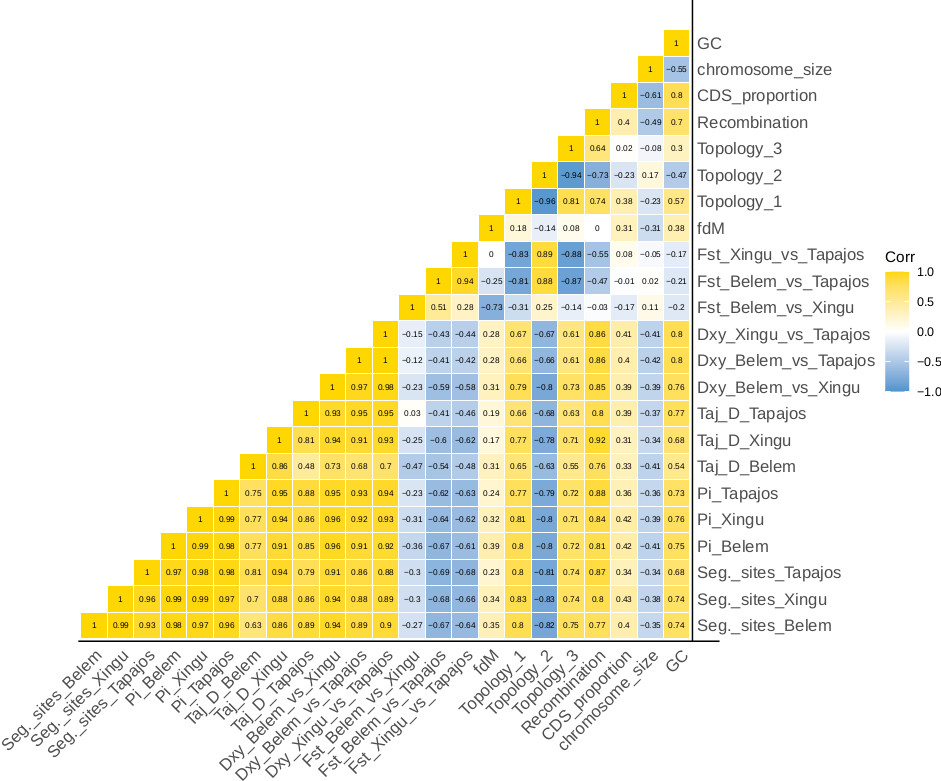


Figure S5: Correlogram (Pearson’s correlation index) between population genetics summary statistics, phylogenetic weights, and genomic architecture estimates for *Xiphorhynchus spixii*, based on the average values per chromosome. P-values are available on supplementary table 10.


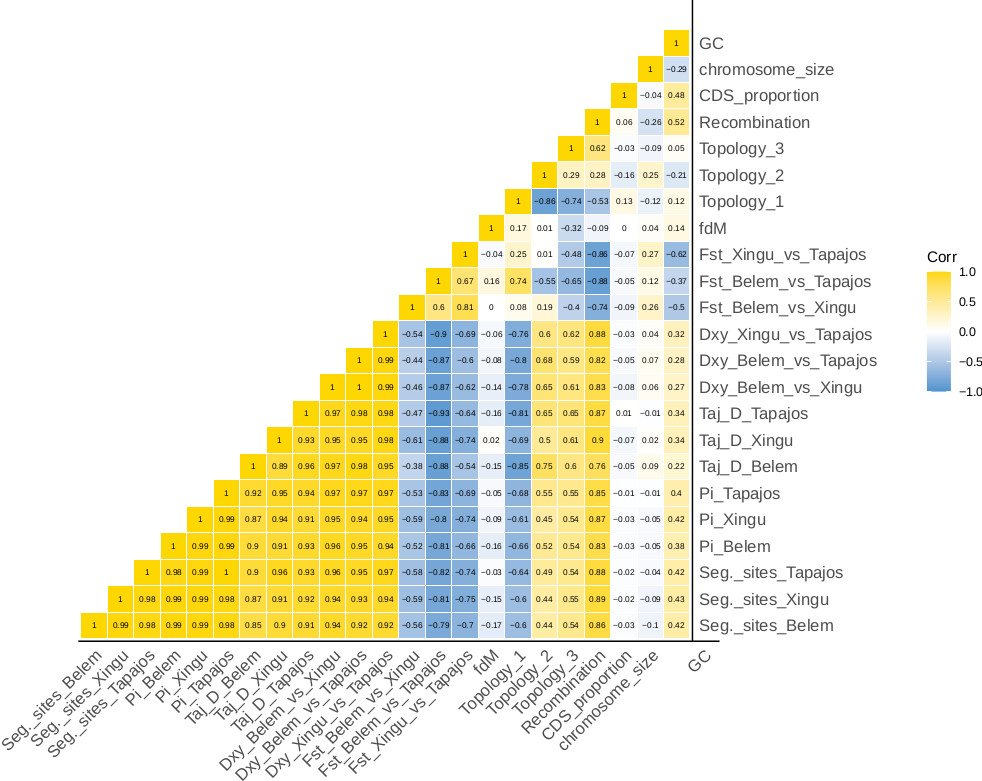


Figure S6: Correlogram (Pearson’s correlation index) between population genetics summary statistics, phylogenetic weights, and genomic architecture estimates for *Lipaugus vociferans*, based on the average values per chromosome. P-values are available in supplementary table 11.


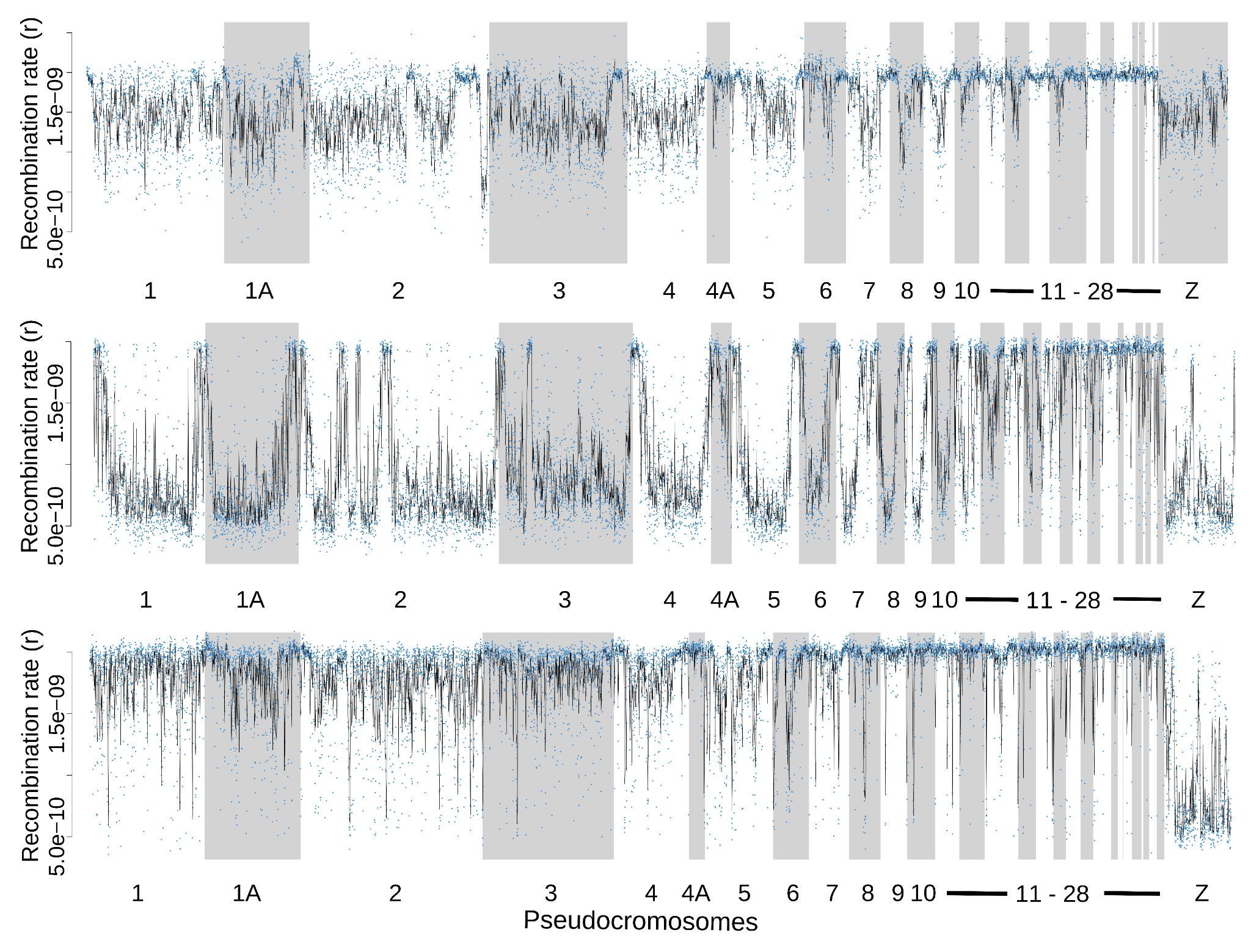


Figure S7: Recombination rate (r = recombination rate per base pair per generation) across the genome of three Amazonian species: *Phlegopsis nigromaculata* (top); *Xiphorhynchus spixii* (center); and *Lipaugus vociferans* (bottom).


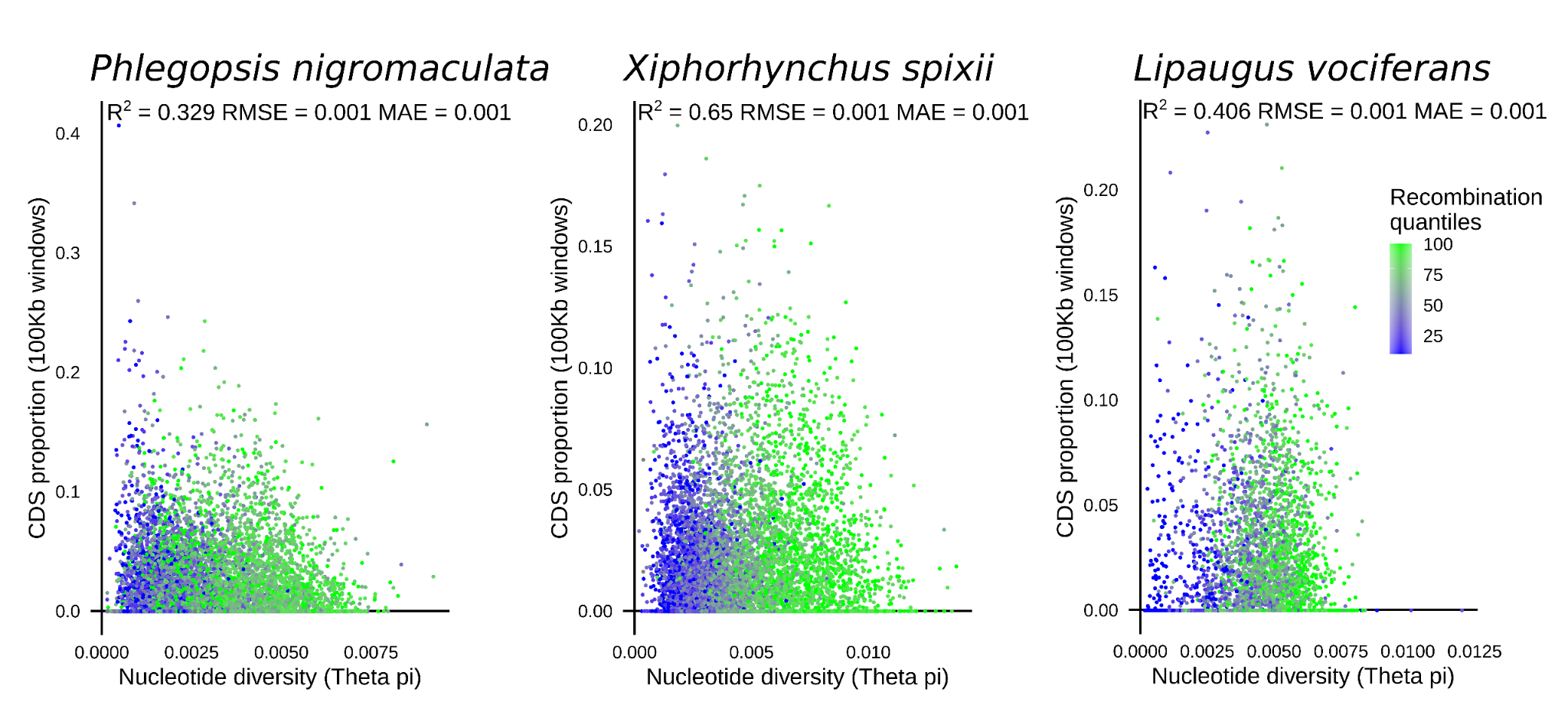


Figure S8: Relationship between nucleotide diversity, gene density and recombination rate for three species of Amazonian birds. R^2^, root mean square error (RMSE) and mean absolute error (MAE) were obtained with a LOESS model with nucleotide diversity as a response variable, and recombination rate and gene density as predictors.


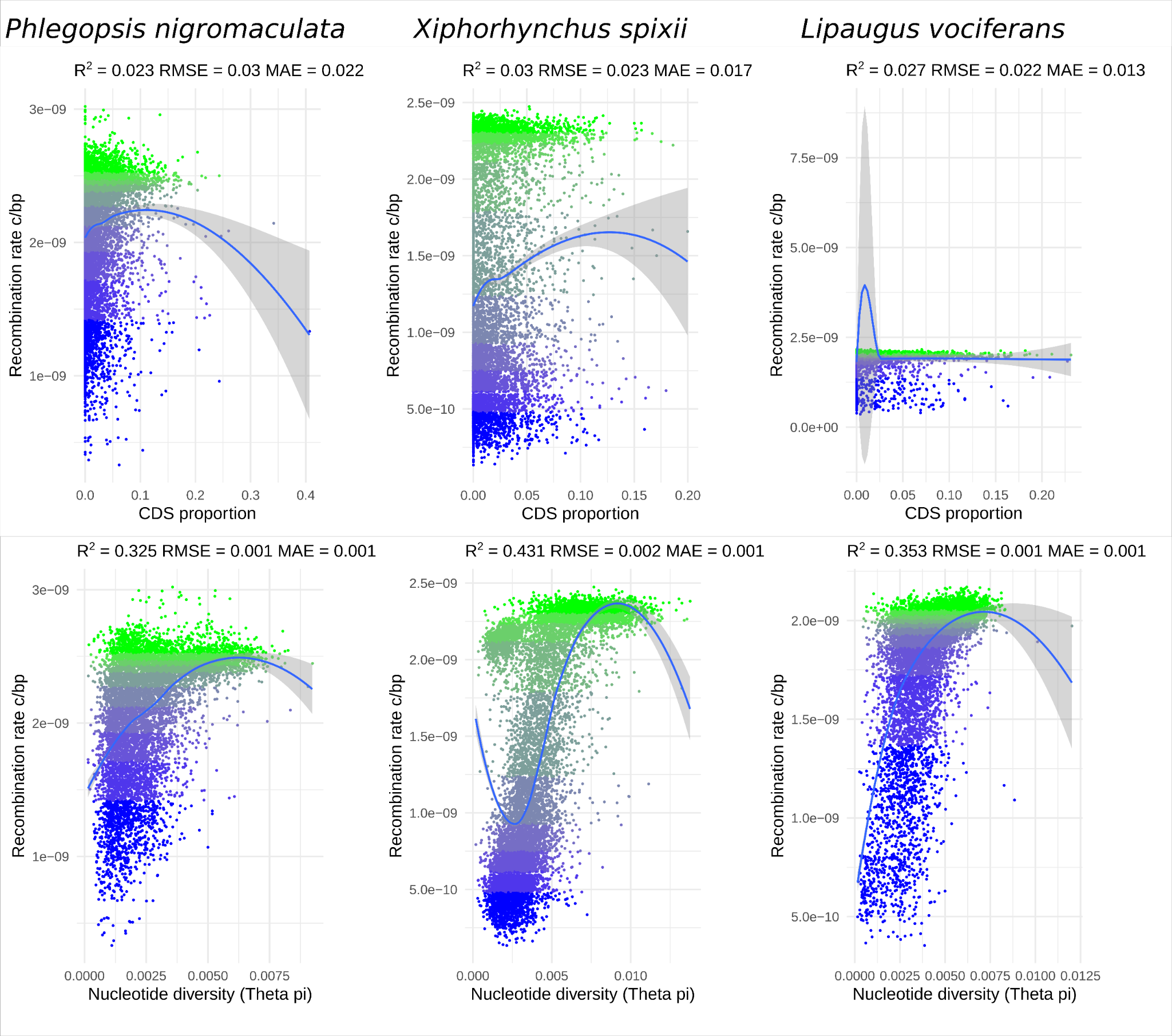


Figure S9: Relationship between nucleotide diversity, gene density and recombination rate for three species of Amazonian Birds. R^2^, root mean square error (RMSE) and mean absolute error (MAE) were obtained with a LOESS model with nucleotide diversity or gene density as response variable, and recombination rate as predictor.


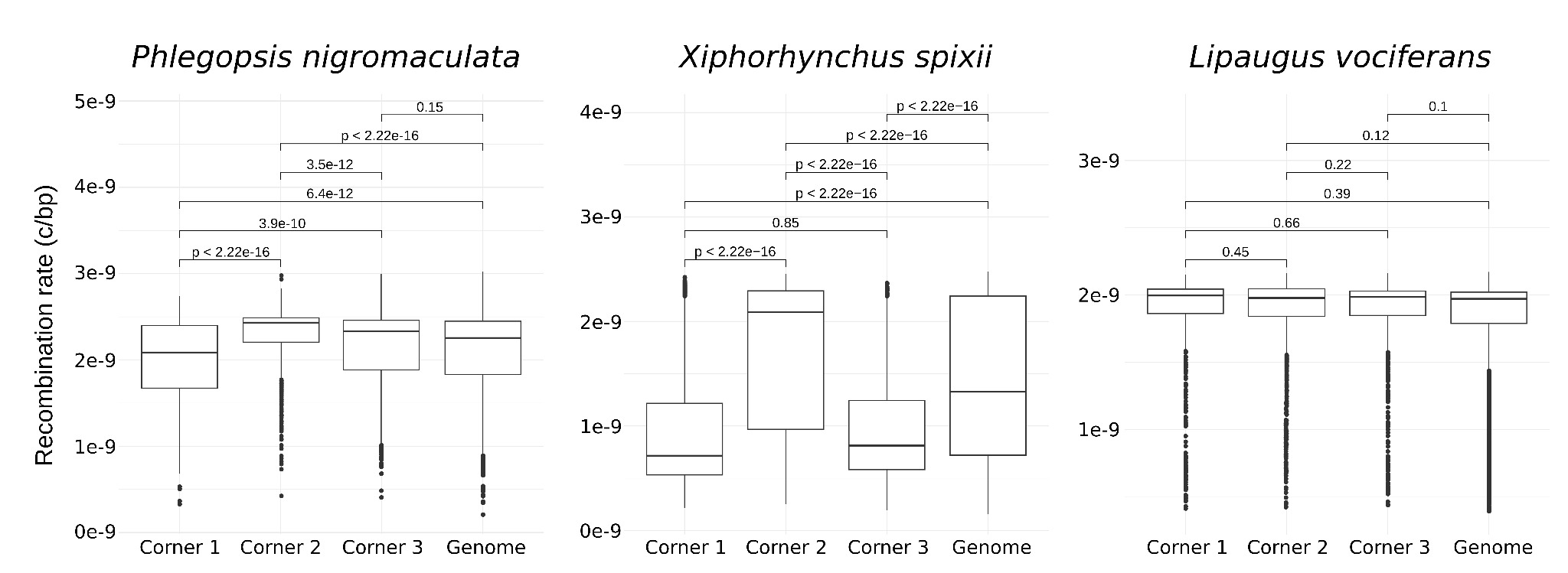


Figure S10: Differences in recombination rate among distinct MDS corners identified with local PCAs, and the whole genome (Genome) for the three studied species. The pairwise comparisons show p-values for Wilcoxon tests. Corners were selected by clustering the 10% of the windows closer to the three further points in the graphs. PCA plots for each corner are available in Figure 1.


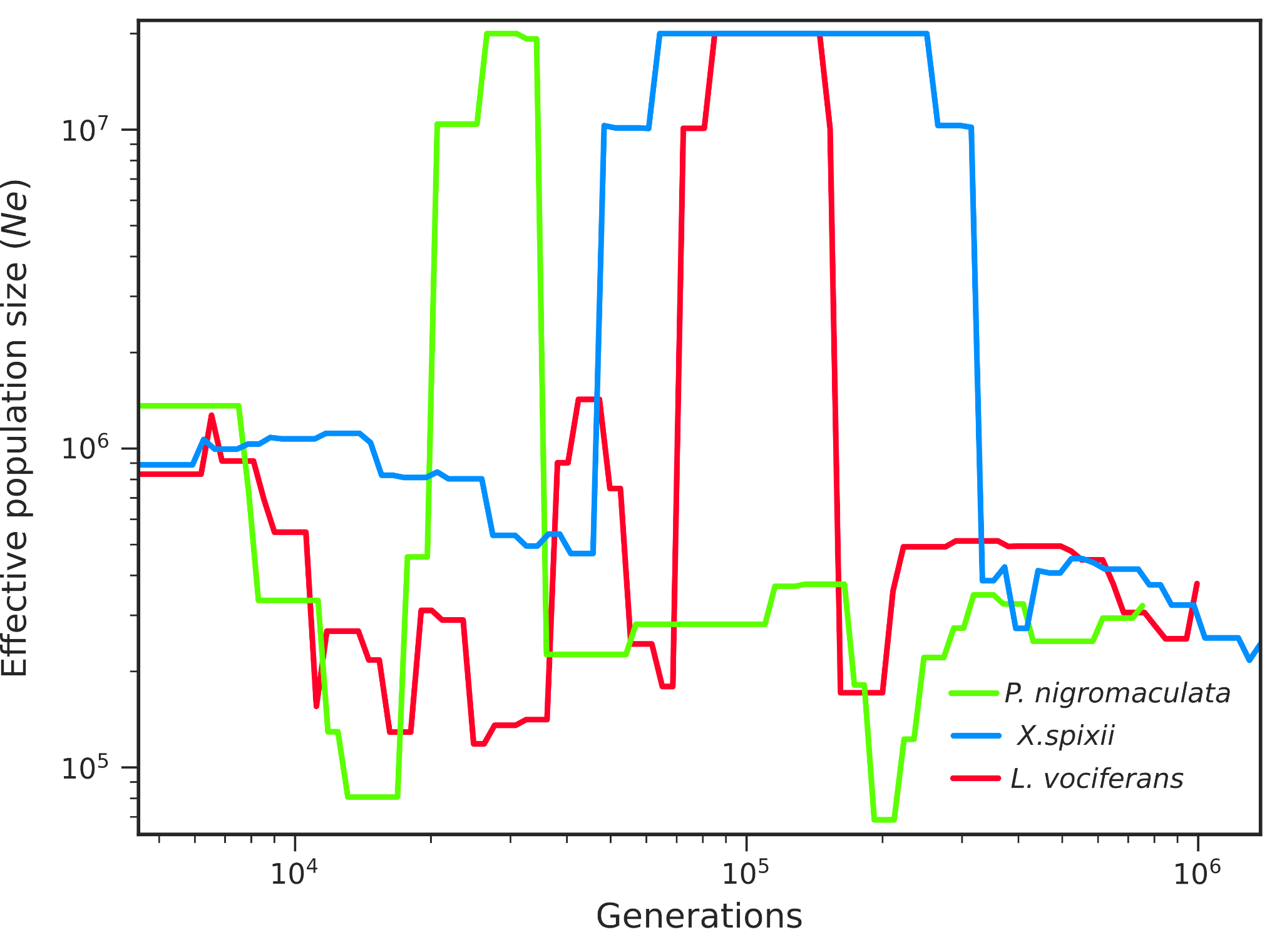


Figure S11: SMC++ estimates of effective population size through time for the Tapajos population of the three studied species. We considered a mutation rate of 2.42 x 10^-9^ mutations per generation and a one year of generation time [(Jarvis et al. 2014; Zhang et al. 2014)](https://paperpile.com/c/oJUljg/UsKO+1LCw).


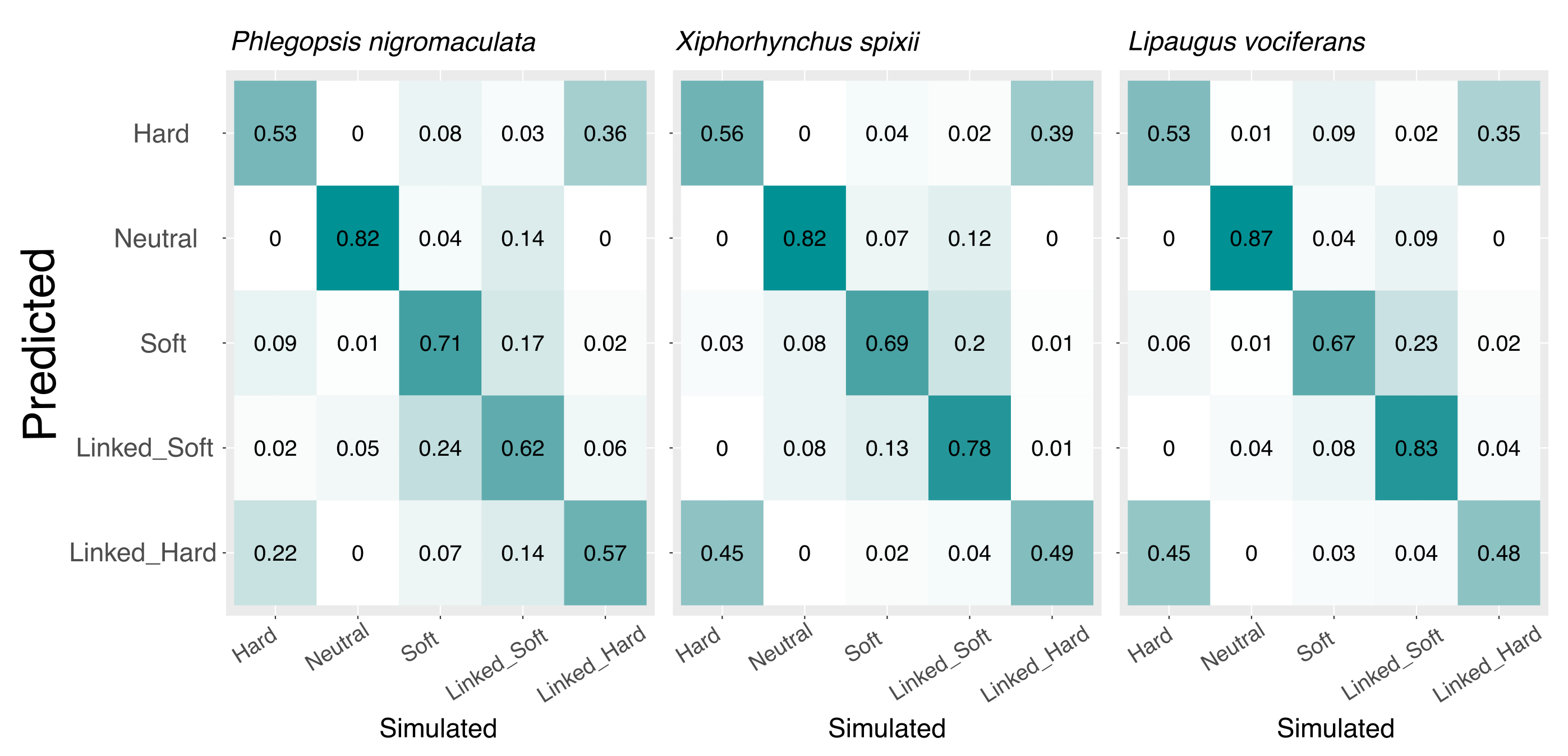


Figure S12: Confusion matrix obtained in the testing step of diploS/HIC


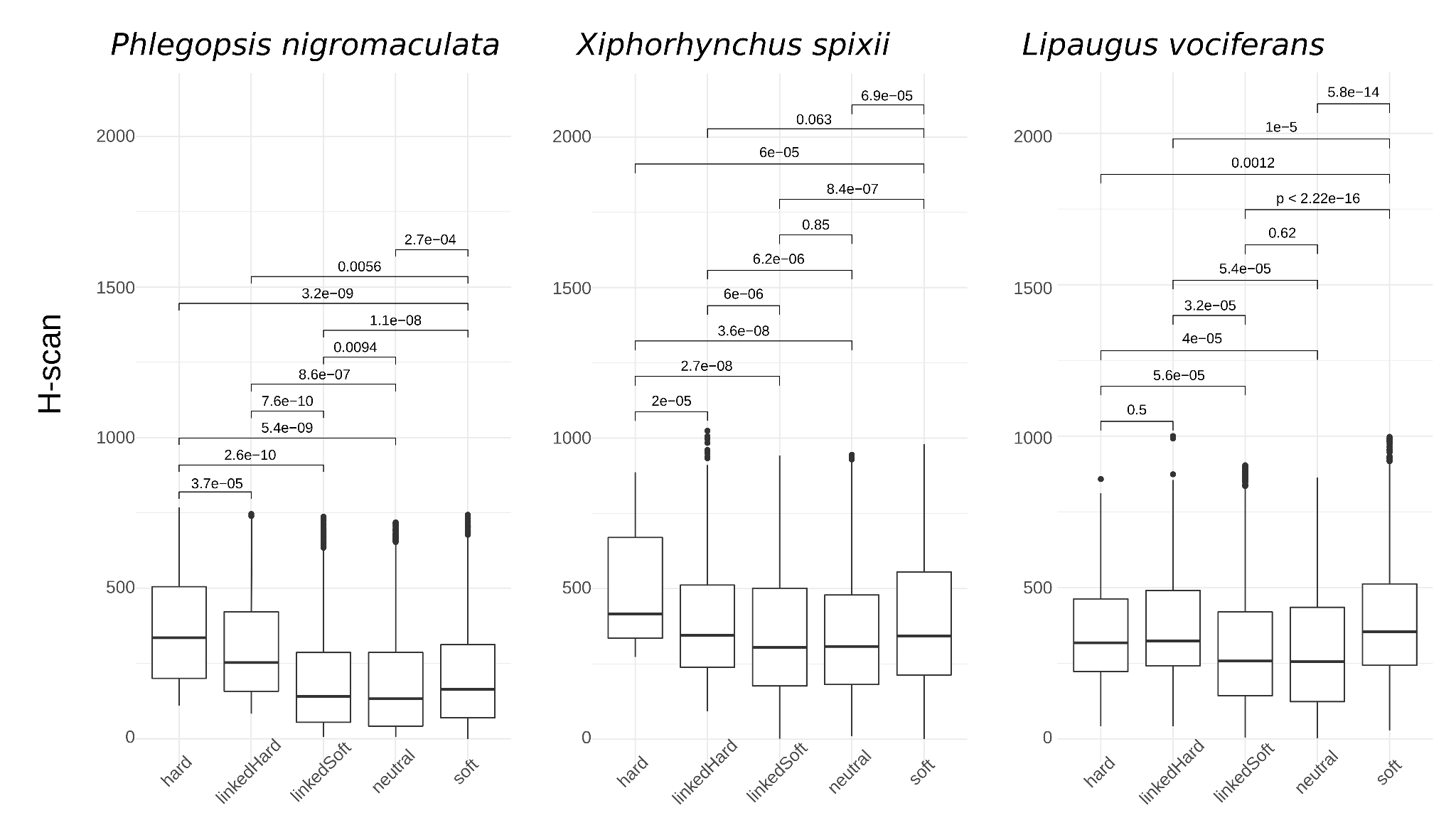


Figure S13: Distribution of pairwise homozygosity tracks between windows of the genome assigned to the five models used on DiploS/Hic. The pairwise comparisons show p-values for Wilcoxon tests.


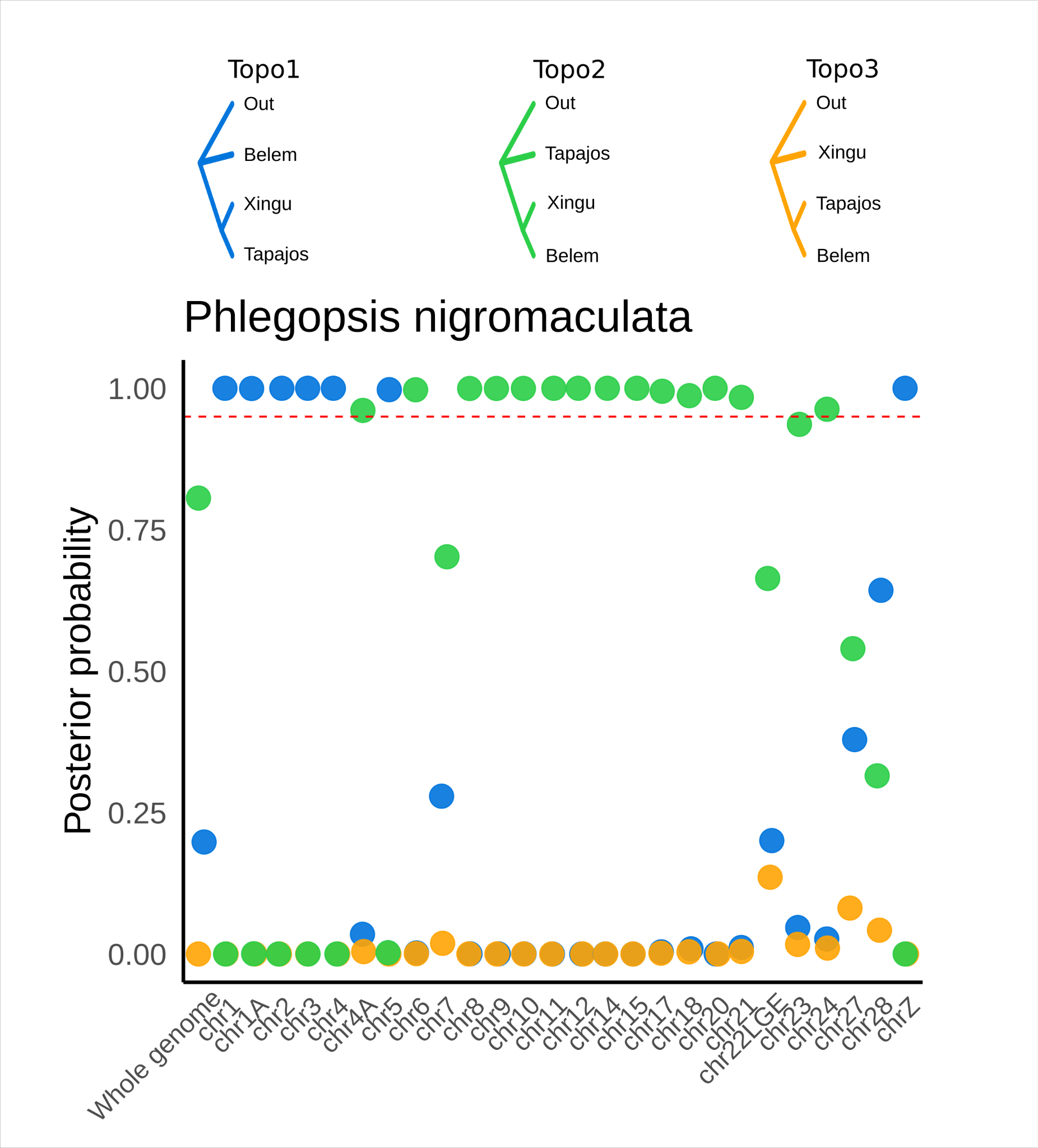


Figure S14: Species tree support across chromosomes of *Phlegopsis nigromaculata.* On top are depicted the three possible unrooted topologies for the relationship between Tapajos, Xingu, and Belem areas of endemism plus an outgroup. Dashed red line on the graph represents the 95% threshold of posterior probability.


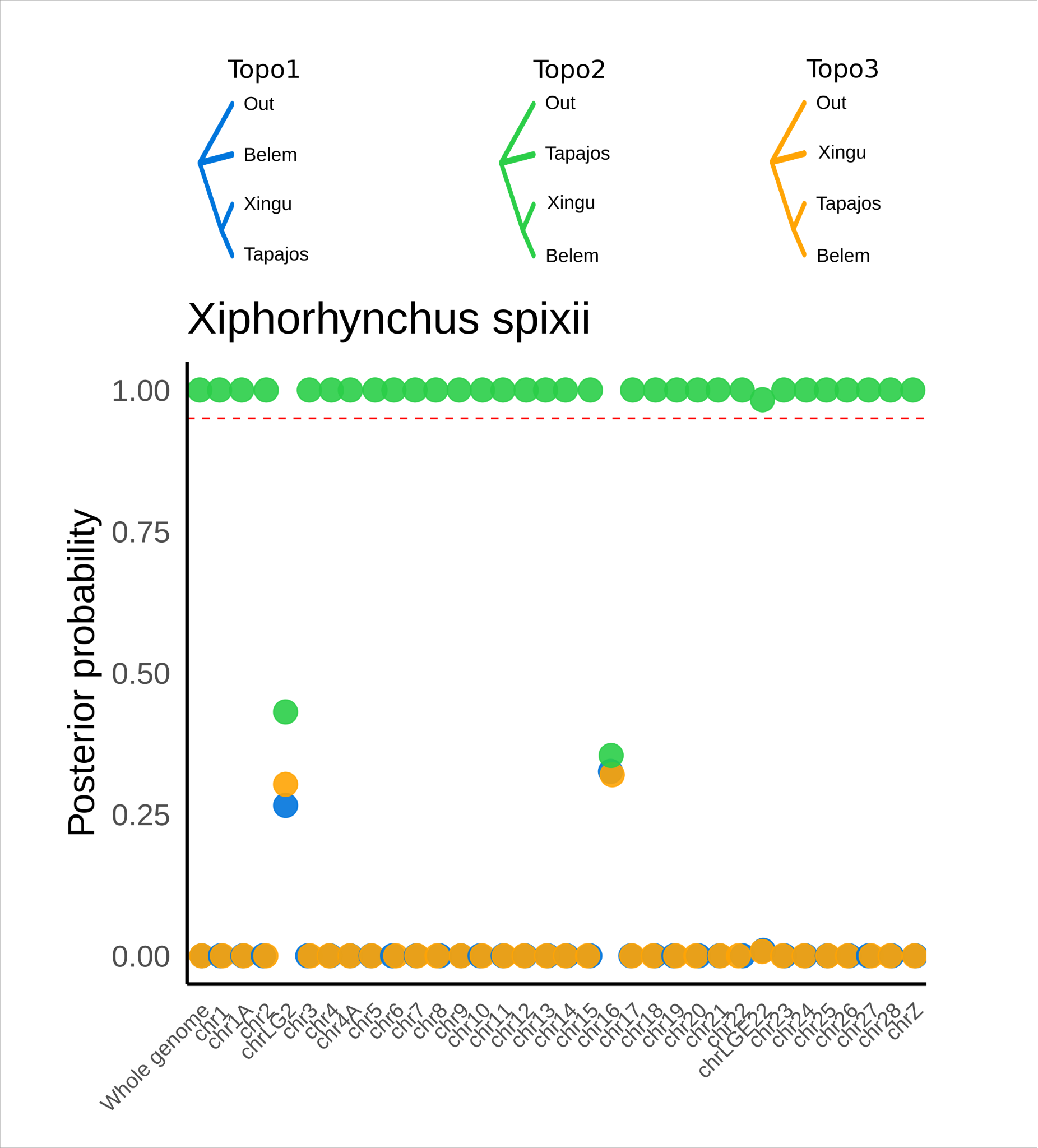


Figure S15: Species tree support across chromosomes of *Xiphorhynchus spixii.* On top are depicted the three possible unrooted topologies for the relationship between Tapajos, Xingu, and Belem areas of endemism plus an outgroup. Dashed red line on the graph represents the 95% threshold of posterior probability.


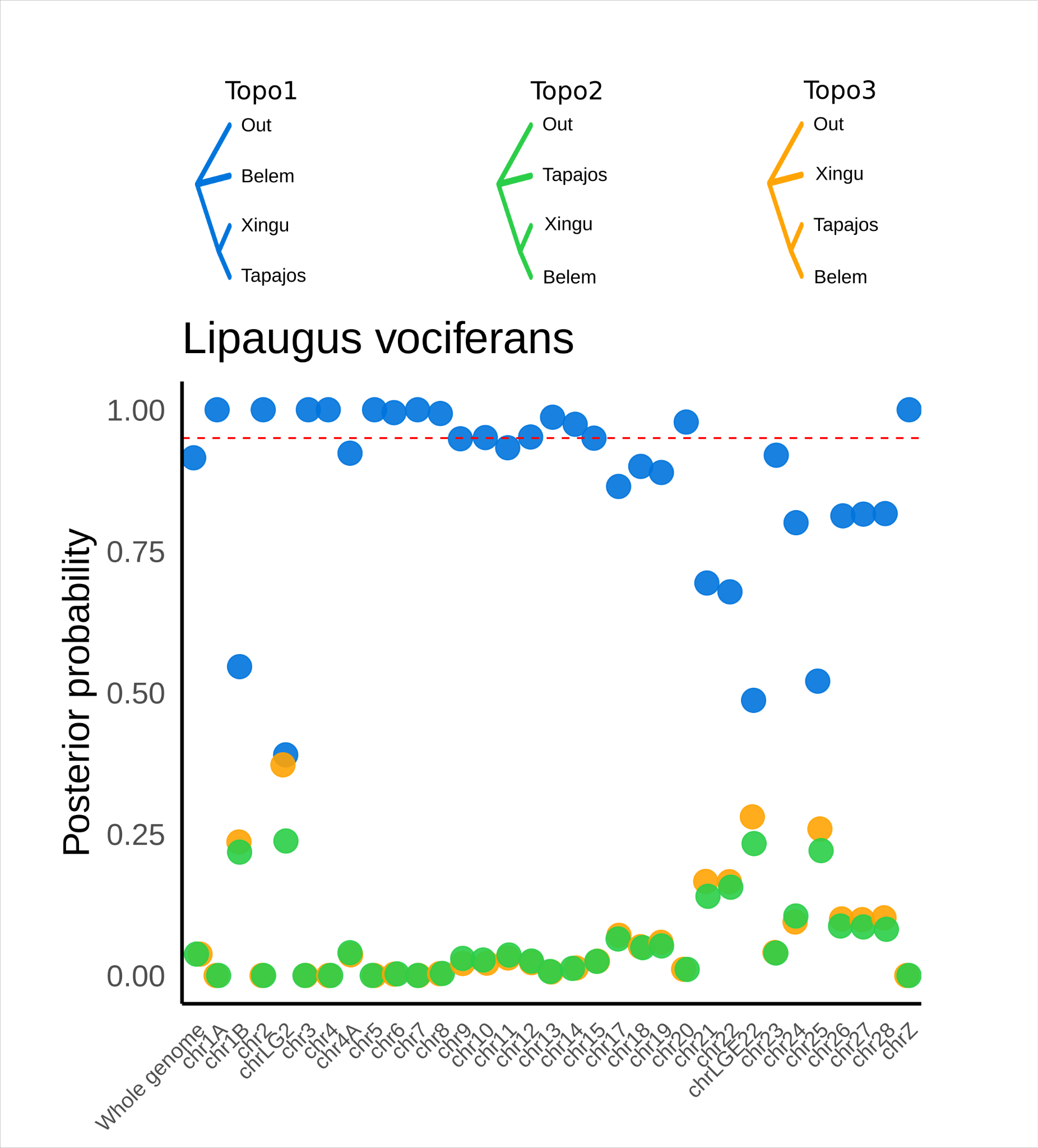


Figure S16: Species tree support across chromosomes of *Lipaugus vociferans.* On top are depicted the three possible unrooted topologies for the relationship between Tapajos, Xingu, and Belem areas of endemism plus an outgroup. Dashed red line on the graph represents the 95% threshold of posterior probability.


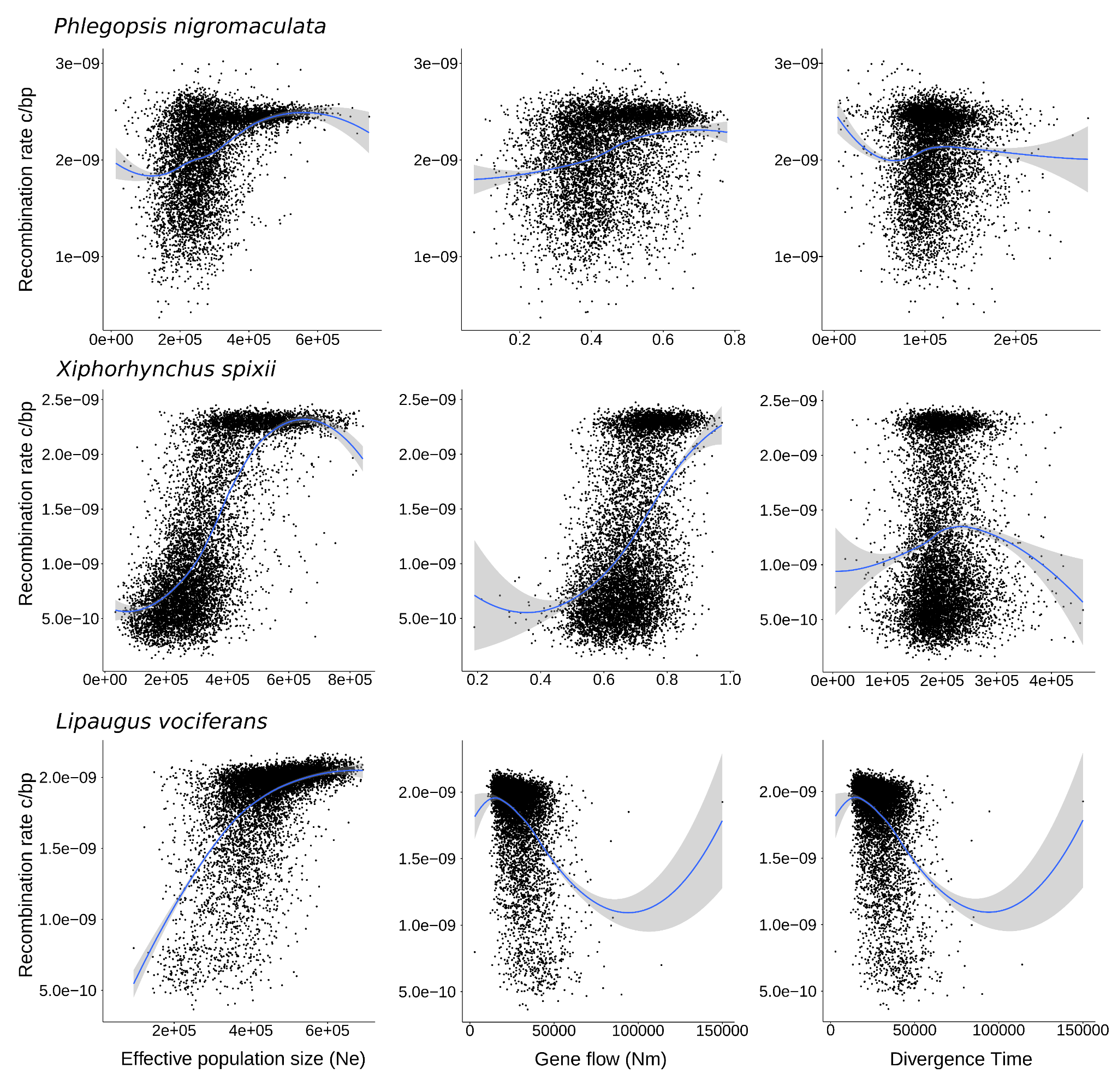
Figure S17: Association between demographic parameters estimated and recombination rate for the three studied species. Ne: Effective population size estimated for the Tapajos population (same population we used to estimate recombination rate). TMRCA: Time to the most recent common ancestor of the most recent divergence event; Gene flow: Gene flow rate between Tapajos and Xingu populations. R^2^, root mean square error (RMSE), and mean absolute error (MAE) for each model are available in table S19.


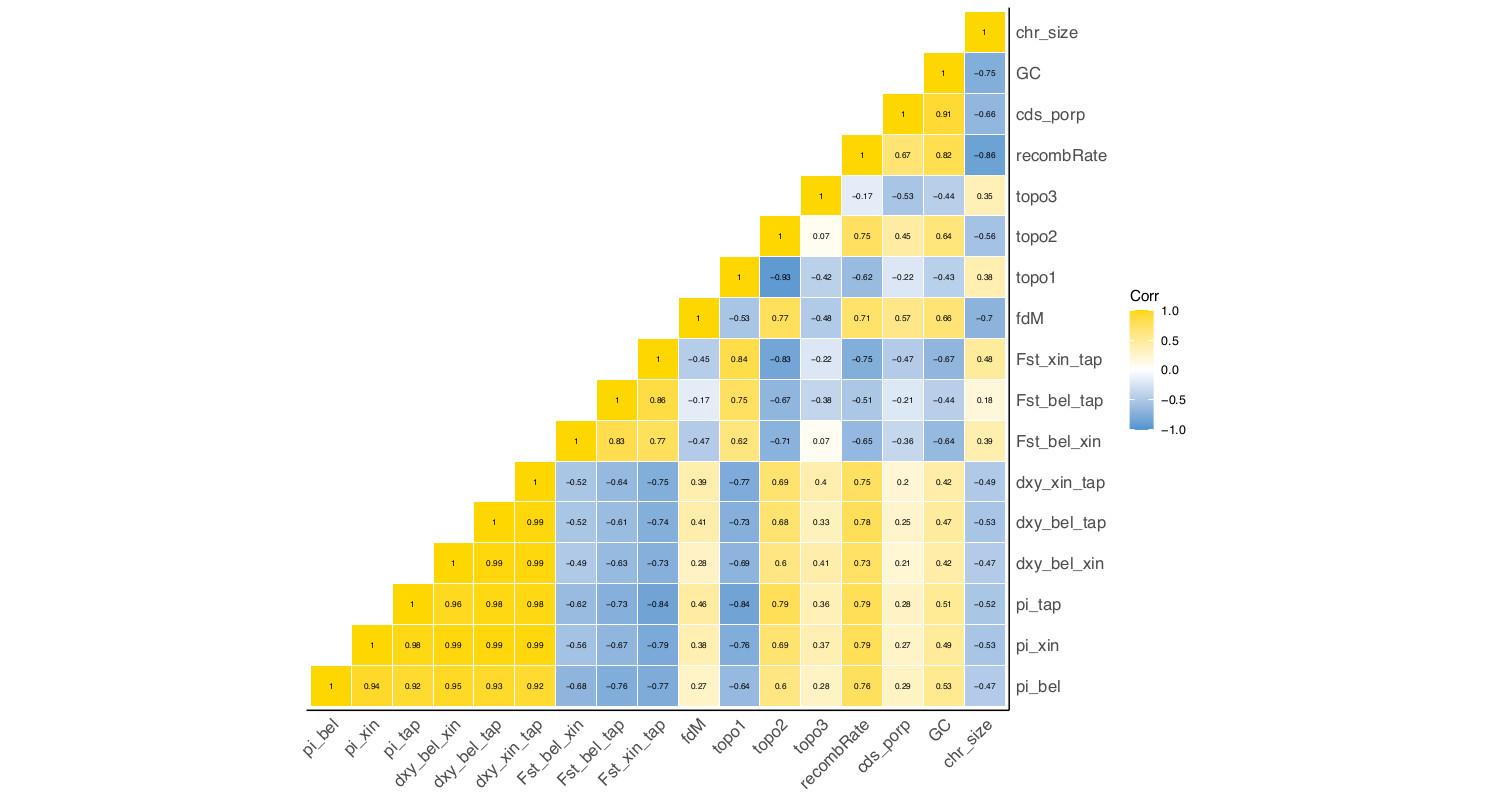


Figure S18: Correlogram (Pearson’s correlation index) between population genetics summary statistics, phylogenetic weights, and genomic architecture estimates for *P. nigromaculata*, based on the average values per chromosome for syntenic regions within homologous chromosomes between the *Rhegmatorhina melanosticta* (pseudochromosomes) and *Chiroxiophia lanceolata* genomes. P-values are available in supplementary table 13.


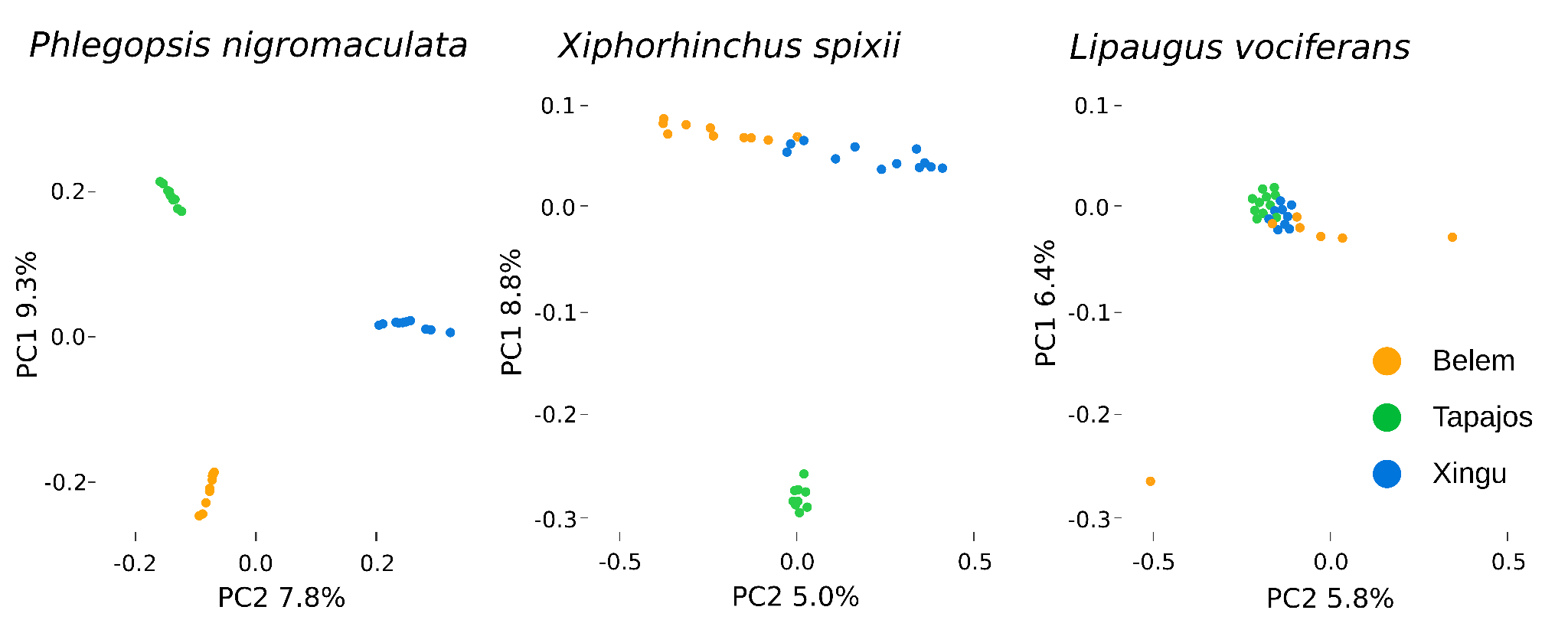


Figure S19: Principal Component Analysis for the three studied species. Colors represent samples collected in different areas of endemism: Tapajos, Xingu, and Belem. Axes are the first and second principal components.


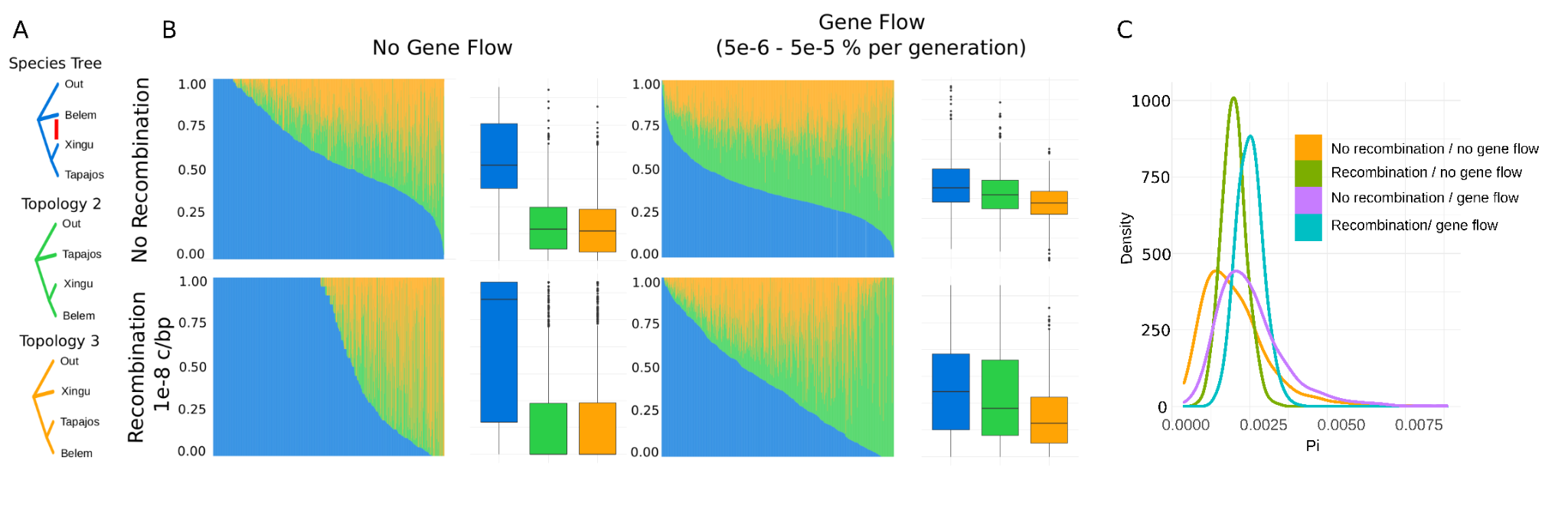


Figure S20: Topology weight and genetic diversity for four simulated models varying the presence of intra-locus recombination and gene flow between non sister lineages. Simulations were conducted assuming the species tree topology **(A)**. **(B)** For each model, the left plot shows the topology weight for the three alternative topologies for 1,000 simulations, and the right plot shows the average weight across simulations. **(C)** Distribution of nucleotide diversity across simulations for models with and without gene flow and intralocus recombination rate.
